# Supplementary material for: The efficacy and safety of prokinetics in critically ill adults receiving gastric feeding tubes: A systematic review and meta-analysis
Source: PLoS One. 2021 Jan 11;16(1):e0245317. doi: 10.1371/journal.pone.0245317 (PMC7799841; doi:10.1371/journal.pone.0245317)
Supplement: S1 Table — (DOCX) [file pone.0245317.s001.docx]

**S1 Table. Search strategy**

Embase search strategy (via OvidSP) From 1974 to November 22, 2019

--------------------------------------------------------------------------------

1. critical illness

2. critical* AND ill*

3. exp *critical illness/

4. exp *intensive care unit/

5. intensive care units

6. intensive And care And unit

7. exp *intensive care/

8. critical care

9. care* AND critical*

10. intensive AND care

11. exp *endotoxemia/

12. endotoxemia

13. endotoxemia*

14. exp *bacteremia/

15. bacteremia

16. bacteremia*

17. exp *sepsis/

18. sepsis

19. septic*

20. pyemia*

21. pyohemia*

22. pyaemia*

23. pyaemia*

24. blood AND poisoning

25. or/1-24

26. exp *enteric feeding/

27. enteral nutrition

28. enteral* and nutrition

29. enteral* AND administration

30. exp *digestive tract intubation/

31. digestive tract intubation

32. enteric feeding

33. intubation, gastrointestinal

34. feed*

35. fed

36. diet

37. food

38. nutrition

39. nutrition*

40. exp *diet/

41. exp *nutrition/

42. 34 or 35 or 36 or 37 or 38 or 39 or 40 or 41

43. enter*

44. enteric

45. enteral

46. force

47. tube

48. intubat*

49. tubal

50. oral*

51. sip

52. alimentary canal

53. gastric

54. intragastric

55. intestinal

56. intraintestinal

57. gastrointestinal

58. nasogastric

59. exp *gastrostomy/

60. gastrostomy

61. gastro*

62. exp *jejunostomy/ or exp *jejunostomy tube/

63. jejunostomy

64. jejunostom*

65. orogastric

66. nasoenteric

67. nasojejunal

68. post-pylor*

69. postpylor*

70. post pylor*

71. stomach

72. nasogastr*

73. naso-gasstric

74. nasal

75. nose

76. duoden*

77. nasoduoden*

78. esophagus

79. fine bore

80. Ryles

81. “PEJ”

82. “PEG”

83. bowel*

84. intestine*

85. intestinal

86. transpylor*

87. trans-pylor*

88. gavage

89. g-tube*

90. ng-tube*

91. j-tube*

92. nj-tube*

93. sump tube

94. percutaneous endoscopic gastrostomy

95. percutaneous radiologic gastrostomy

96. surgical gastrostomy

97. or/43-96

98. 42 and 97

99. 26 or 27 or 28 or 29 or 30 or 31 or 32 or 33 or 98

100. exp *erythromycin/

101. erythromycin

102. T-Stat

103. T Stat

104. TStat

105. erymax

106. erycette

107. ilotycin

108. 100 or 101 or 102 or 103 or 104 or 105 or 106 or 107

109. exp *metoclopramide/

110. metoclopramide

111. 4-Amino-5-chloro-N-(2-(diethylamino)ethyl)-2-methoxybenzamide

112. metaclopramide

113. maxolon

114. rimetin

115. primperan

116. reglan

117. cerucal

118. 109 or 110 or 111 or 112 or 113 or 114 or 115 or 116 or 117

119. exp *domperidone/

120. domperidone

121. domperidon-TEVA

122. domperidon

123. domidon

124. domperidona

125. gastrocure

126. motilium

127. nauzelin

128. novo-Domperidone

129. nu-Domperidone

130. PMS-Domperidone

131. Péridys

132. R-33,812

133. R33,812

134. R-33812

135. R33812

136. ratio-Domperidone

137. apo-Domperidone

138. or/119-137

139. exp *itopride/

140. itopride

141. N-(p-(2-(dimethylamino)ethoxy)benzyl)veratramide hydrochloride

142. 139 or 140 or 141

143. exp *cisapride/

144. cisapride

145. R-51619

146. R 51619

147. R51619

148. propulsid

149. 143 or 144 or 145 or 146 or 147 or 148

150. exp *tegaserod/

151. tegaserod

152. tegaserode

153. 5-methoxyindol-3-carboxaldehyde amino(pentylamino) methylenehydrazone hydrogen maleate

154. zelnorm

155. zelmac

156. SDZ HTF 919

157. HTF 919

158. SDZ HTF-919

159. 150 or 151 or 152 or 153 or 154 or 155 or 156 or 157 or 158

160. exp *mosapride/

161. mosapride

162. 4-amino-5-chloro-2-ethoxy-N-((4-(4-fluorobenzyl)-2-morpholinyl)methyl)benzamide

163. AS 4370

164. AS-4370

165. 160 or 161 or 162 or 163 or 164

166. exp *renzapride/

167. renzapride

168. 4-amino-5-chloro-2-methoxy-N-(1-azabicyclo-(3.3.1)-non-4-yl)benzamide

169. BRL 24924

170. BRL-24924

171. 166 or 167 or 168 or 169 or 170

172. exp *trimebutine/

173. trimebutine

174. TM-906

175. TM 906

176. TM906

177. transacalm

178. polibutin

179. modulon

180. debridat

181. 172 or 173 or 174 or 175 or 176 or 177 or 178 or 179 or 180

182. exp *antiemetic agent/

183. antiemetics

184. antiemetic agent

185. antiemetic

186. anti-emetics

187. anti emetics

188. anti-Emetic

189. anti Emetic

190. 182 or 183 or 184 or 185 or 186 or 187 or 188 or 189

191. exp *prokinetic agent/

192. prokinetic agent

193. prokinetic

194. exp *motilin/

195. motilin

196. motility agent*

197. exp *gastrointestinal motility/

198. gastrointestinal motility

199. gastric motility

200. gastric emptying

201. or/191-200

202. 108 or 118 or 138 or 142 or 149 or 159 or 165 or 171 or 181 or 190 or 201

203. 25 and 99 and 202

204. exp *randomized controlled trial/

205. exp *controlled clinical trial/

206. randomi?ed.ab,ti.

207. placebo.ab.

208. *Clinical Trial/

209. randomly.ab.

210. trial.ti.

211. or/204-210

212. exp *human/

213. exp *animal/

214. 212 and 213

215. 213 not 214

216. 211 not 215

217. 216 and 203

MEDLINE search strategy (via OvidSP) From 1946 to November 22, 2019

--------------------------------------------------------------------------------

1. critical illness

2. critical* AND ill*

3. exp *critical illness/

4. exp *intensive care unit/

5. intensive care units

6. intensive And care And unit

7. exp *intensive care/

8. critical care

9. care* AND critical*

10. intensive AND care

11. exp *endotoxemia/

12. endotoxemia

13. endotoxemia*

14. exp *bacteremia/

15. bacteremia

16. bacteremia*

17. exp *sepsis/

18. sepsis

19. septic*

20. pyemia*

21. pyohemia*

22. pyaemia*

23. pyaemia*

24. blood AND poisoning

25. or/1-24

26. exp *enteric feeding/

27. enteral nutrition

28. enteral* and nutrition

29. enteral* AND administration

30. exp *digestive tract intubation/

31. digestive tract intubation

32. enteric feeding

33. intubation, gastrointestinal

34. feed*

35. fed

36. diet

37. food

38. nutrition

39. nutrition*

40. exp *diet/

41. exp *nutrition/

42. 34 or 35 or 36 or 37 or 38 or 39 or 40 or 41

43. enter*

44. enteric

45. enteral

46. force

47. tube

48. intubat*

49. tubal

50. oral*

51. sip

52. alimentary canal

53. gastric

54. intragastric

55. intestinal

56. intraintestinal

57. gastrointestinal

58. nasogastric

59. exp *gastrostomy/

60. gastrostomy

61. gastro*

62. exp *jejunostomy/ or exp *jejunostomy tube/

63. jejunostomy

64. jejunostom*

65. orogastric

66. nasoenteric

67. nasojejunal

68. post-pylor*

69. postpylor*

70. post pylor*

71. stomach

72. nasogastr*

73. naso-gasstric

74. nasal

75. nose

76. duoden*

77. nasoduoden*

78. esophagus

79. fine bore

80. Ryles

81. “PEJ”

82. “PEG”

83. bowel*

84. intestine*

85. intestinal

86. transpylor*

87. trans-pylor*

88. gavage

89. g-tube*

90. ng-tube*

91. j-tube*

92. nj-tube*

93. sump tube

94. percutaneous endoscopic gastrostomy

95. percutaneous radiologic gastrostomy

96. surgical gastrostomy

97. or/43-96

98. 42 and 97

99. 26 or 27 or 28 or 29 or 30 or 31 or 32 or 33 or 98

100. exp *erythromycin/

101. erythromycin

102. T-Stat

103. T Stat

104. TStat

105. erymax

106. erycette

107. ilotycin

108. 100 or 101 or 102 or 103 or 104 or 105 or 106 or 107

109. exp *metoclopramide/

110. metoclopramide

111. 4-Amino-5-chloro-N-(2-(diethylamino)ethyl)-2-methoxybenzamide

112. metaclopramide

113. maxolon

114. rimetin

115. primperan

116. reglan

117. cerucal

118. 109 or 110 or 111 or 112 or 113 or 114 or 115 or 116 or 117

119. exp *domperidone/

120. domperidone

121. domperidon-TEVA

122. domperidon

123. domidon

124. domperidona

125. gastrocure

126. motilium

127. nauzelin

128. novo-Domperidone

129. nu-Domperidone

130. PMS-Domperidone

131. Péridys

132. R-33,812

133. R33,812

134. R-33812

135. R33812

136. ratio-Domperidone

137. apo-Domperidone

138. or/119-137

139. exp *itopride/

140. itopride

141. N-(p-(2-(dimethylamino)ethoxy)benzyl)veratramide hydrochloride

142. 139 or 140 or 141

143. exp *cisapride/

144. cisapride

145. R-51619

146. R 51619

147. R51619

148. propulsid

149. 143 or 144 or 145 or 146 or 147 or 148

150. exp *tegaserod/

151. tegaserod

152. tegaserode

153. 5-methoxyindol-3-carboxaldehyde amino(pentylamino)methylenehydrazone hydrogen maleate

154. zelnorm

155. zelmac

156. SDZ HTF 919

157. HTF 919

158. SDZ HTF-919

159. 150 or 151 or 152 or 153 or 154 or 155 or 156 or 157 or 158

160. exp *mosapride/

161. mosapride

162. 4-amino-5-chloro-2-ethoxy-N-((4-(4-fluorobenzyl)-2-morpholinyl)methyl)benzamide

163. AS 4370

164. AS-4370

165. 160 or 161 or 162 or 163 or 164

166. exp *renzapride/

167. renzapride

168. 4-amino-5-chloro-2-methoxy-N-(1-azabicyclo-(3.3.1)-non-4-yl)benzamide

169. BRL 24924

170. BRL-24924

171. 166 or 167 or 168 or 169 or 170

172. exp *trimebutine/

173. trimebutine

174. TM-906

175. TM 906

176. TM906

177. transacalm

178. polibutin

179. modulon

180. debridat

181. 172 or 173 or 174 or 175 or 176 or 177 or 178 or 179 or 180

182. exp *antiemetic agent/

183. antiemetics

184. antiemetic agent

185. antiemetic

186. anti-emetics

187. anti emetics

188. anti-Emetic

189. anti Emetic

190. 182 or 183 or 184 or 185 or 186 or 187 or 188 or 189

191. exp *prokinetic agent/

192. prokinetic agent

193. prokinetic

194. exp *motilin/

195. motilin

196. motility agent*

197. exp *gastrointestinal motility/

198. gastrointestinal motility

199. gastric motility

200. gastric emptying

201. or/191-200

202. 108 or 118 or 138 or 142 or 149 or 159 or 165 or 171 or 181 or 190 or 201

203. 25 and 99 and 202

204. exp *randomized controlled trial/

205. exp *controlled clinical trial/

206. randomi?ed.ab,ti.

207. placebo.ab.

208. *Clinical Trial/

209. randomly.ab.

210. trial.ti.

211. or/204-210

212. exp *human/

213. exp *animal/

214. 212 and 213

215. 213 not 214

216. 211 not 215

217. 216 and 203

218. exp *Enteral Nutrition/

219. digestive tract intubation

220. enteric feeding

221. nutrition

222. 34 or 35 or 36 or 37 or 38 or 39 or 40 or 221

223. 222 and 97

224. 218 or 219 or 220 or 223 or 27 or 28 or 29 or 31 or 32 or 33

225. 25 and 202 and 217 and 224

CENTRAL search strategy (via OvidSP) From the inception dates to October 2019

--------------------------------------------------------------------------------

1. critical illness

2. critical* AND ill*

3. exp *critical illness/

4. exp *intensive care unit/

5. intensive care units

6. intensive And care And unit

7. exp *intensive care/

8. critical care

9. care* AND critical*

10. intensive AND care

11. exp *endotoxemia/

12. endotoxemia

13. endotoxemia*

14. exp *bacteremia/

15. bacteremia

16. bacteremia*

17. exp *sepsis/

18. sepsis

19. septic*

20. pyemia*

21. pyohemia*

22. pyaemia*

23. pyaemia*

24. blood AND poisoning

25. or/1-24

26. exp *enteric feeding/

27. enteral nutrition

28. enteral* and nutrition

29. enteral* AND administration

30. exp *digestive tract intubation/

31. digestive tract intubation

32. enteric feeding

33. intubation, gastrointestinal

34. feed*

35. fed

36. diet

37. food

38. nutrition

39. nutrition*

40. exp *diet/

41. exp *nutrition/

42. 34 or 35 or 36 or 37 or 38 or 39 or 40 or 41

43. enter*

44. enteric

45. enteral

46. force

47. tube

48. intubat*

49. tubal

50. oral*

51. sip

52. alimentary canal

53. gastric

54. intragastric

55. intestinal

56. intraintestinal

57. gastrointestinal

58. nasogastric

59. exp *gastrostomy/

60. gastrostomy

61. gastro*

62. exp *jejunostomy/ or exp *jejunostomy tube/

63. jejunostomy

64. jejunostom*

65. orogastric

66. nasoenteric

67. nasojejunal

68. post-pylor*

69. postpylor*

70. post pylor*

71. stomach

72. nasogastr*

73. naso-gasstric

74. nasal

75. nose

76. duoden*

77. nasoduoden*

78. esophagus

79. fine bore

80. Ryles

81. “PEJ”

82. “PEG”

83. bowel*

84. intestine*

85. intestinal

86. transpylor*

87. trans-pylor*

88. gavage

89. g-tube*

90. ng-tube*

91. j-tube*

92. nj-tube*

93. sump tube

94. percutaneous endoscopic gastrostomy

95. percutaneous radiologic gastrostomy

96. surgical gastrostomy

97. or/43-96

98. 42 and 97

99. 26 or 27 or 28 or 29 or 30 or 31 or 32 or 33 or 98

100. exp *erythromycin/

101. erythromycin

102. T-Stat

03. T Stat

104. TStat

105. erymax

106. erycette

107. ilotycin

108. 100 or 101 or 102 or 103 or 104 or 105 or 106 or 107

109. exp *metoclopramide/

110. metoclopramide

111. 4-Amino-5-chloro-N-(2-(diethylamino)ethyl)-2-methoxybenzamide

112. metaclopramide

113. maxolon

114. rimetin

115. primperan

116. reglan

117. cerucal

118. 109 or 110 or 111 or 112 or 113 or 114 or 115 or 116 or 117

119. exp *domperidone/

120. domperidone

121. domperidon-TEVA

122. domperidon

123. domidon

124. domperidona

125. gastrocure

126. motilium

127. nauzelin

128. novo-Domperidone

129. nu-Domperidone

130. PMS-Domperidone

131. Péridys

132. R-33,812

133. R33,812

134. R-33812

135. R33812

136. ratio-Domperidone

137. apo-Domperidone

138. or/119-137

139. exp *itopride/

140. itopride

141. N-(p-(2-(dimethylamino)ethoxy)benzyl)veratramide hydrochloride

142. 139 or 140 or 141

143. exp *cisapride/

144. cisapride

145. R-51619

146. R 51619

147. R51619

148. propulsid

149. 143 or 144 or 145 or 146 or 147 or 148

150. exp *tegaserod/

151. tegaserod

152. tegaserode

153. 5-methoxyindol-3-carboxaldehyde amino(pentylamino)methylenehydrazone hydrogen maleate

154. zelnorm

155. zelmac

156. SDZ HTF 919

157. HTF 919

158. SDZ HTF-919

159. 150 or 151 or 152 or 153 or 154 or 155 or 156 or 157 or 158

160. exp *mosapride/

161. mosapride

162. 4-amino-5-chloro-2-ethoxy-N-((4-(4-fluorobenzyl)-2-morpholinyl)methyl)benzamide

163. AS 4370

164. AS-4370

165. 160 or 161 or 162 or 163 or 164

166. exp *renzapride/

167. renzapride

168. 4-amino-5-chloro-2-methoxy-N-(1-azabicyclo-(3.3.1)-non-4-yl)benzamide

169. BRL 24924

170. BRL-24924

171. 166 or 167 or 168 or 169 or 170

172. exp *trimebutine/

173. trimebutine

174. TM-906

175. TM 906

176. TM906

177. transacalm

178. polibutin

179. modulon

180. debridat

181. 172 or 173 or 174 or 175 or 176 or 177 or 178 or 179 or 180

182. exp *antiemetic agent/

183. antiemetics

184. antiemetic agent

185. antiemetic

186. anti-emetics

187. anti emetics

188. anti-Emetic

189. anti Emetic

190. 182 or 183 or 184 or 185 or 186 or 187 or 188 or 189

191. exp *prokinetic agent/

192. prokinetic agent

193. prokinetic

194. exp *motilin/

195. motilin

196. motility agent*

197. exp *gastrointestinal motility/

198. gastrointestinal motility

199. gastric motility

200. gastric emptying

201. or/191-200

202. 108 or 118 or 138 or 142 or 149 or 159 or 165 or 171 or 181 or 190 or 201

203. 25 and 99 and 202

204. exp *Intensive Care Units/

205. 25 or 204

206. exp *Gastrointestinal Tract/

207. 99 or 206

208. Gastrointestinal Motility/ or Gastric Emptying/

209. or/100-200

210. 208 or 209

211. 205 and 207 and 210
