# Supplementary material for: The efficacy and safety of prokinetics in critically ill adults receiving gastric feeding tubes: A systematic review and meta-analysis
Source: PLoS One. 2021 Jan 11;16(1):e0245317. doi: 10.1371/journal.pone.0245317 (PMC7799841; doi:10.1371/journal.pone.0245317)
Supplement: S2 Table — (DOCX) [file pone.0245317.s002.docx]

**S2 Table. Excluded studies**

| **Article Number** | **Full reference** | **Reason for Exclusion** |
| --- | --- | --- |
| 1 | Taylor SJ, Allan K, McWilliam H, Manara A, Brown J, Greenwood R, Toher D: A randomised controlled feasibility and proof-of-concept trial in delayed gastric emptying when metoclopramide fails: we should revisit nasointestinal feeding versus dual prokinetic treatment: achieving goal nutrition in critical illness and delayed gastric emptying: trial of nasointestinal feeding versus nasogastric feeding plus prokinetics. Clinical nutrition ESPEN 2016, 14:1-8. | **Different trial design:**  Gastric feeding was discontinued or interrupted prematurely, “The first GRV ≥250 mL was discarded and EN was continued at the same rate but a second consecutive 4 hourly GRV≥ 250 mL was discarded and the feed rate was reduced 50%” in this study. |
| 2 | Pinilla JC, Samphire J, Arnold C, Liu L, Thiessen B: Comparison of gastrointestinal tolerance to two enteral feeding protocols in critically ill patients: a prospective, randomized controlled trial. Journal of Parenteral and Enteral Nutrition 2001, 25(2):81-6. | **Different trial design:**  Gastric feeding was discontinued or interrupted prematurely: if vomiting or a high gastric RV (≥150 mL in group I or 250ml in group II) was detected, the feedings were held for 4 hours, restarted at 25 mL/h, and increased by 25 mL/h withheld for q2 hours until the goal rate was reached. After a second intolerance episode, the feedings were 6 hours, restarted at 50% of the goal rate, and increased by 25 mL/h q4 hours until the goal rate was achieved in this study. |
| 3 | Chapman MJ, Fraser RJ, Kluger MT, Buist MD, De Nichilo DJ: Erythromycin improves gastric emptying in critically ill patients intolerant of nasogastric feeding. Critical care medicine 2000, 28:2334-2337. | **Different trial design:**  Gastric feeding was discontinued or interrupted prematurely, successful enteral feeding was defined as a gastric volume <250 mL, and feeding was continued. Failure was defined as a gastric aspirate ≥250 mL at least 6 hrs after commencing feed of ≥40 mL/hr. |
| 4 | Reignier J, Bensaid S, Perrin-Gachadoat D, Burdin M, Boiteau R, Tenaillon A: Erythromycin and early enteral nutrition in mechanically ventilated patients. Critical care medicine 2002, 30:1237-1241. | **Different trial design:**  Gastric feeding was discontinued or interrupted prematurely, enteral nutrition was discontinued if residual gastric volume exceeded 250 mL or the patient vomited. |
| 5 | Deane AM, Lamontagne F, Dukes GE, Neil D, Vasist L, Barton ME, Hacquoil K, Ou X, Richards D, Stelfox HT, et al: Nutrition Adequacy Therapeutic Enhancement in the Critically Ill: a Randomized Double-Blind, Placebo-Controlled Trial of the Motilin Receptor Agonist Camicinal (GSK962040): the NUTRIATE Study. Journal of parenteral and enteral nutrition 2018, 42:949-959. | **Different trial design:**  Gastric feeding was discontinued or interrupted prematurely, withdrawal and stopping criteria were predefined and included established feed intolerance (two consecutive GRV measurements>250 mL), transition to small-bowel feeding, EN discontinued, initiation of renal replacement therapy or estimated glomerular filtration rate<20 mL/min, impaired liver chemistry, or QT prolongation. |
| 6 | Nguyen N, Chapman M, Fraser R, Sharley V, Kong S, Bryant L, et al.: Erythromycin or metoclopramide for feed intolerance in the critically ill. Critical care 2006, 10. | **Different control:**  Without placebo or no treatment group |
| 7 | MacLaren R, Patrick WD, Hall RI, Rocker GM, Whelan GJ, Lima JJ: Comparison of cisapride and metoclopramide for facilitating gastric emptying and improving tolerance to intragastric enteral nutrition in critically III, mechanically ventilated adults. Clinical therapeutics 2001, 23:1855-1866. | **Different control:**  Without placebo or no treatment group |
| 8 | Heyland DK, van Zanten ARH, Grau-Carmona T, Evans D, Beishuizen A, Schouten J, Hoiting O, Bordeje ML, Krell K, Klein DJ, et al: A multicenter, randomized, double-blind study of ulimorelin and metoclopramide in the treatment of critically ill patients with enteral feeding intolerance: PROMOTE trial. Intensive care medicine 2019, 45:647-656. | **Different control:**  Without placebo or no treatment group |
| 9 | Boivin MA, Levy H: Gastric feeding with erythromycin is equivalent to transpyloric feeding in the critically ill. Critical care medicine 2001, 29:1916-1919. | **Different control:**  Feeding through a transpylorically placed feeding tube |
| 10 | Taylor S, Manara A, Brown J: Treating delayed gastric emptying in critical illness: metoclopramide, erythromycin and bedside (Cortrak[TM]) nasointestinal tube placement. Journal of the intensive care society 2011, 12:75-. | **Different control:**  without placebo or no treatment group, the control group by feeding through electromagnetically-guided nasointestinal tube |
| 11 | Irct201112014578N: The effect of acupuncture and traditional pharmacologic therapy on Delayed Gastric Emptying. http://wwwwhoint/trialsearch/Trial2aspx?TrialID=IRCT201112014578N4 2012. | **Different control:**  without placebo or no treatment group |
| 12 | Nct: A Randomized, Double-Blind Study to Evaluate the Safety, Tolerability, and Pharmacodynamics of a Single Dose of Intravenous TD-8954 Compared With Metoclopramide in Critically Ill Patients With Enteral Feeding Intolerance. https://clinicaltrialsgov/show/NCT01953081 2013. | **Different control:**  without placebo or no treatment group |
| 13 | Yanagida Y, Hayakawa M, Yamamoto H, Wada T, Sugano M, Sawamura A, Gando S: Effects of rikkunshito (traditional Japanese medicine Kampo) on enteral feeding and plasma ghrelin concentration in critically ill patients: a double-blind, randomized, controlled trial. Intensive care medicine 2013, 39:S242-S243. | **Different control:**  without placebo or no treatment group |
| 14 | Hayakawa M, Ono Y, Wada T, Yanagida Y, Sawamura A, Takeda H, Gando S: Effects of rikkunshito (traditional Japanese medicine) on enteral feeding and the plasma ghrelin level in critically ill patients: a pilot study. Journal of intensive care 2014, 2. | **Different control:**  without placebo or no treatment group |
| 15 | Irct201412044365N: Comparison of the effect of Neostigmine and Metoclopramide on gastric residual volume in mechanically ventilated ICU patients. http://wwwwhoint/trialsearch/Trial2aspx?TrialID=IRCT201412044365N18 2014. | **Different control:**  without placebo or no treatment group |
| 16 | Irct201408104365N: Combination of Neostigmine and Metoclopramide to reduce gastric residual volume in mechanically ventilated ICU patients. http://wwwwhoint/trialsearch/Trial2aspx?TrialID=IRCT201408104365N16 2015. | **Different control:**  without placebo or no treatment group |
| 17 | Euctr ES: A Phase 2, Multicenter, Randomized, Double-Blind, Comparator-Controlled Study of the Efficacy, Safety, and Pharmacokinetics of Intravenous Ulimorelin (LP101) in Patients with Enteral Feeding Intolerance. http://wwwwhoint/trialsearch/Trial2aspx?TrialID=EUCTR2016 2016. | **Different control:**  without placebo or no treatment group |
| 18 | Makkar JK, Gauli B, Jain K, Jain D, Batra YK: Comparison of erythromycin versus metoclopramide for gastric feeding intolerance in patients with traumatic brain injury: a randomized double-blind study. Saudi journal of anaesthesia 2016, 10:308-313. | **Different control:**  Without placebo or no treatment group, gastric feeding intolerance was defined as GAV more than 150 ml with abdominal symptoms. Two consecutive high GAV was defined as feeding failure. Feeding failure was treated by increasing the frequency of dose to 6 hourly in metoclopramide and erythromycin group. Combination therapy with both drugs was given as rescue in the placebo group. |
| 19 | Malekolkottab M, Khalili H, Mohammadi M, Ramezani M, Nourian A: Metoclopramide as intermittent and continuous infusions in critically ill patients: a pilot randomized clinical trial. Journal of comparative effectiveness research 2017, 6:127-136. | **Different control:**  without placebo or no treatment group |
| 20 | Nct: Itopride in Feeding Intolerance of Critically-ill Patients Receiving Enteral Nutrition. https://clinicaltrialsgov/show/NCT03698292. 2018. | **Different control:**  without placebo or no treatment group |
| 21 | Meissner W, Dohrn B, Reinhart K: Enteral naloxone reduces gastric tube reflux and frequency of pneumonia in critical care patients during opioid analgesia. Critical care medicine 2003, 31:776-780. | **Different intervention:**  Naloxone |
| 22 | Berne JD, Norwood SH, McAuley CE, Vallina VL, Villareal D, Weston J, McClarty J: Erythromycin reduces delayed gastric emptying in critically ill trauma patients: a randomized, controlled trial. Journal of trauma 2002, 53:422-425. | **Different intervention:**  "Success" of therapy was defined as "no gastric residual greater than 150 mL" during the first 48 hours after therapy for DGE. Patients with continued intolerance for 48 hours after randomization were considered failures of therapy and changed to metoclopramide. |
| 23 | **S**hariatpanahi ZV, Taleban FA, Mokhtari M, Shahbazi S: Ginger extract reduces delayed gastric emptying and nosocomial pneumonia in adult respiratory distress syndrome patients hospitalized in an intensive care unit. Journal of critical care 2010, 25:647-650. | **Different intervention:**  If there were no gastric residual volume greater than 150 mL during this period on ginger extracts supplemental therapy, it was defined as success of therapy; but continued feed intolerance after this period was considered failure of therapy. Patients in this group were then given erythromycin. |
| 24 | Tamion F, Hamelin K, Duflo A, Girault C, Richard JC, Bonmarchand G: Gastric emptying in mechanically ventilated critically ill patients: effect of neuromuscular blocking agent. Intensive care medicine 2003, 29:1717-1722. | **Different intervention:**  Opiate sedation (fentanyl/midazolam) and neuromuscular blocking agent (cisatracurium) |
| 25 | Goldhill DR, Toner CC, Tarling MM, Baxter K, Withington PS, Whelpton R: Double-blind, randomized study of the effect of cisapride on gastric emptying in critically ill patients. Critical care medicine 1997, 25:447-451. | **Different population:**  The patients did not receive enteral nutrition |
| 26 | Chen JH, Hsieh CB, Chao PC, Liu HD, Chen CJ, Liu YC, Yu JC: Effect of water-soluble contrast in colorectal surgery: a prospective randomized trial. World journal of gastroenterology 2005, 11:2802-2805 | **Different population:**  The patients were non-critically ill |
| 27 | Warusevitane A, Karunatilake D, Sim J, Lally F, Roffe C: Safety and effect of metoclopramide to prevent pneumonia in patients with stroke fed via nasogastric tubes trial. Stroke 2015, 46:454-460. | **Different population:**  The patients were non-critically ill |
| 28 | 2015/08/006111 C: Clinical trial to compare the efficacy of two oral prokinetics metoclopramide and erythromycin in non acceptance of feed in head injury patient. http://wwwwhoint/trialsearch/Trial2aspx?TrialID=CTRI 2015, 08. | **Outcomes not reported** |
| 29 | Tctr: Efficacy and safety of oral Erythromycin estolate in combination with Metoclopramide versus Metoclopramide mOnotherapy in mechanically ventilated patients who developed enteral feeding intolerance: a randomized double-blind controlled study. http://wwwwhoint/trialsearch/Trial2aspx?TrialID=TCTR20171004004 2017. | **Outcomes not reported** |
| 30 | IRCT201610297494N24: The prophylactic effect of cuminum cyminum extract on gastric residual volume in traumatic patients under ventilator hospitalized in intensive care unit. http://wwwwhoint/trialsearch/Trial2aspx?TrialID=IRCT201610297494N24 2017. | **Outcomes not reported** |
| 31 | NCT02379624: Pectin Start Early Enteral Nutritional Support in Critically Ill Patients. https://clinicaltrialsgov/show/NCT02379624 2014. | **Outcomes not reported** |
| 32 | NCT02528760: To Determine the Role of Prokinetics in Feed Intolerance in Critically Ill Cirrhosis. https://clinicaltrialsgov/show/NCT02528760 2015. | **Outcomes not reported** |
| 33 | IRCT201009094722N2: The effect of Ginger extract on gastric residual volume in patients with mechanical ventilation hospitalized in intensive care unit. http://wwwwhoint/trialsearch/Trial2aspx?TrialID=IRCT201009094722N2 2011. | **Outcomes not reported** |
