# Supplementary material for: The efficacy and safety of prokinetics in critically ill adults receiving gastric feeding tubes: A systematic review and meta-analysis
Source: PLoS One. 2021 Jan 11;16(1):e0245317. doi: 10.1371/journal.pone.0245317 (PMC7799841; doi:10.1371/journal.pone.0245317)
Supplement: S3 Table — (DOCX) [file pone.0245317.s003.docx]

**S3 Table. Separate effects of different prokinetic agents on hospital or ICU length of stay**

| **Study** | **Population (Sample size)** | **Intervention** | **ICU length of stay and** **hospital length of stay** | ***P* Value** | **Conclusions** |
| --- | --- | --- | --- | --- | --- |
| Acosta-Escribano et al. 2014 (Spain) | Adult neuro-critical patients, Glasgow Comma Score14 <to 9 points (109) | 1) Metoclopramide 10 mg i.v.;  2) Placebo: physiological saline | ICU length of stay (days); hospital length of stay (days)  14±8; 36±29  15±9; 31±21 | NS; NS | There was no difference between Metoclopramide group and placebo group in ICU length of stay and hospital length of stay. |
| Kooshki et al. 2018 (Iran) | Mixed intensive care unit patients (60) | 1) Fenugreek seed powder 3g q12h NG;  2) Routine care | ICU length of stay (days); hospital length of stay (days)  14.2±4.7; 24.1±5.6  17.6±6.5; 27.4±6.6 | 0.028; 0.041 | ICU length of stay and hospital length of stay were shorter in group of fenugreek seed powder. |
| Doi et al. 2019 (Japan) | Mixed intensive care unit patients | **1) Rikkunshito 5 g** q8h × 5 days;  2) Rikkunshito 2.5 g q8h× 5 days;  3) No rikkunshito (control). | ICU length of stay (days); hospital length of stay (days) †  5(4-14); 42(26-48)  6(3-8); 35(30-40)  7(4-9); 56(35-82) | NS; NS | ICU length of stay and hospital length of stay were similar in the three groups. |
| Nursal et al. 2007 (Turkey) | Traumatic brain injury patients with Glasgow Coma Scale scores of 3–11 (19) | 1) Metoclopramide 10 mg i.v. q8h×5 days  2) Control group: saline | Hospital length of stay (days)  15.6±11.1  16.8±8.5 | NS | There was no significant difference in hospital length of stay between these two groups. |
| Guo JH, et al. 2012 (China) | Mixed intensive care unit patients (80) | 1) **Traditional Chinese medicine group:** **Chenxia Sijunzi decoction**  2) Western medicine group: mosapride dispersible tablets 5mg and multienzyme tablets NG  3) Control group: routine symptomatically treated without any medicines for promoting gastrointestinal power function | Hospital length of stay (days)  5.1±1.7  5.0±1.5  8.9±1.4 | ^a^*P*<0.01 | Compared with the control group, Chenxia Sijunzi decoction can shorten hospital length of stay in critical ill patients. But there were not any significant differences in hospital length of stay between traditional Chinese medicine and western medicine group. |
