# Supplementary figures and images for: The efficacy and safety of prokinetics in critically ill adults receiving gastric feeding tubes: A systematic review and meta-analysis
Source: PLoS One. 2021 Jan 11;16(1):e0245317. doi: 10.1371/journal.pone.0245317 (PMC7799841; doi:10.1371/journal.pone.0245317)

**S1 Fig. Risk of bias**


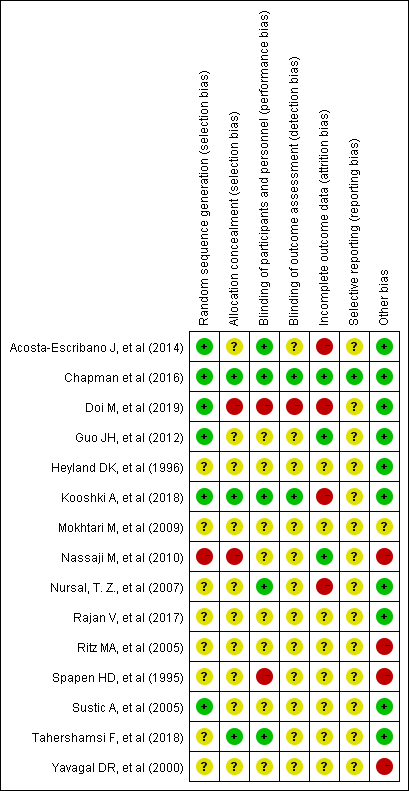

Supplement: S1 Fig — (DOCX) [file pone.0245317.s005.docx]

**S2 Fig. Reported adverse event outcomes**

**
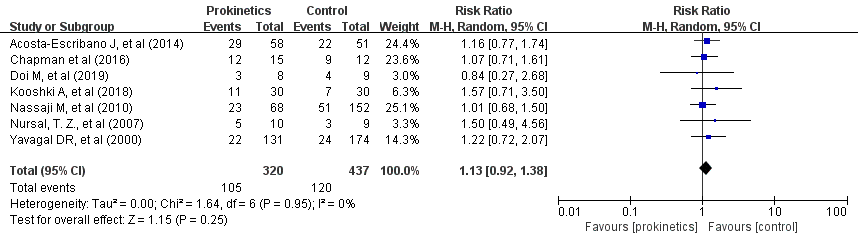
**

Supplement: S2 Fig — (DOCX) [file pone.0245317.s006.docx]

**S3 Fig. All-cause mortality outcomes**

**
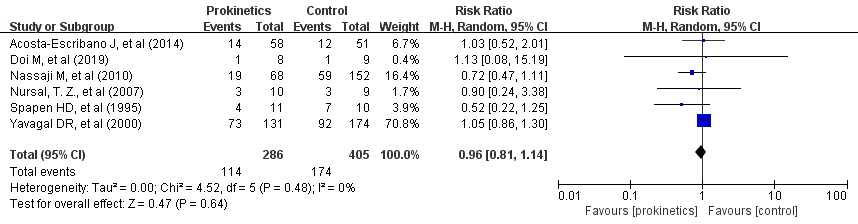
**

Supplement: S3 Fig — (DOCX) [file pone.0245317.s007.docx]

**S4 Fig. Subgroup analysis by the type of prokinetic agents for hospital length of stay outcomes**

**
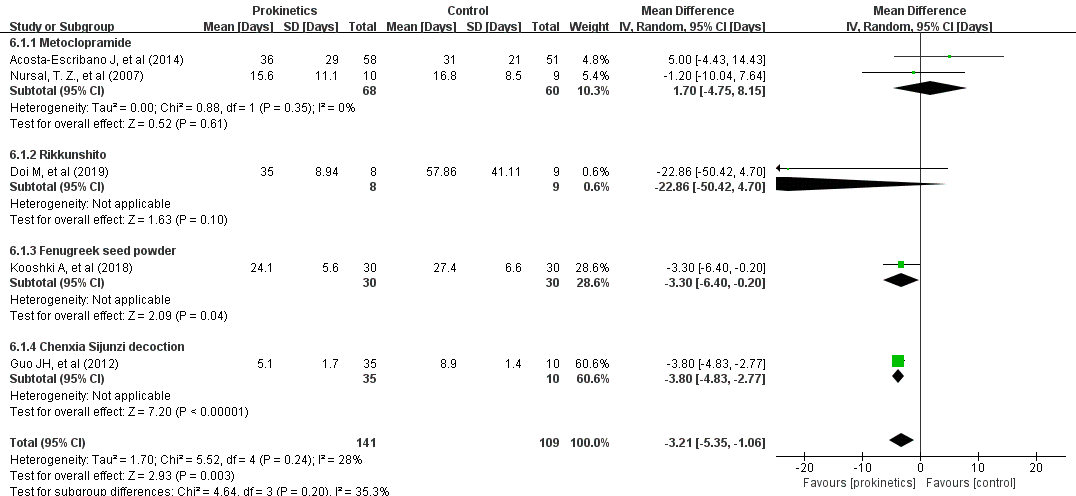
**

Supplement: S4 Fig — (DOCX) [file pone.0245317.s008.docx]

**S5 Fig.** **Subgroup analysis by the type of prokinetic agents for ICU length of stay outcomes**

**
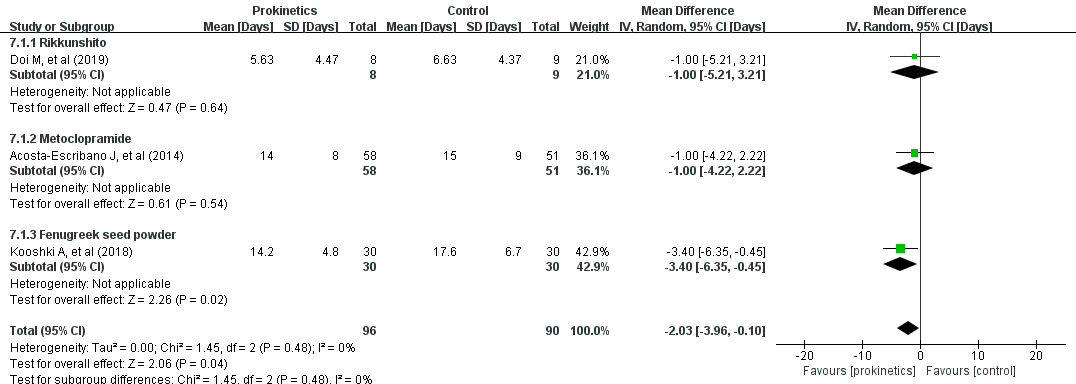
**

Supplement: S5 Fig — (DOCX) [file pone.0245317.s009.docx]

**S6 Fig. Subgroup analysis by the type of prokinetic agents for reported adverse event outcomes**

**
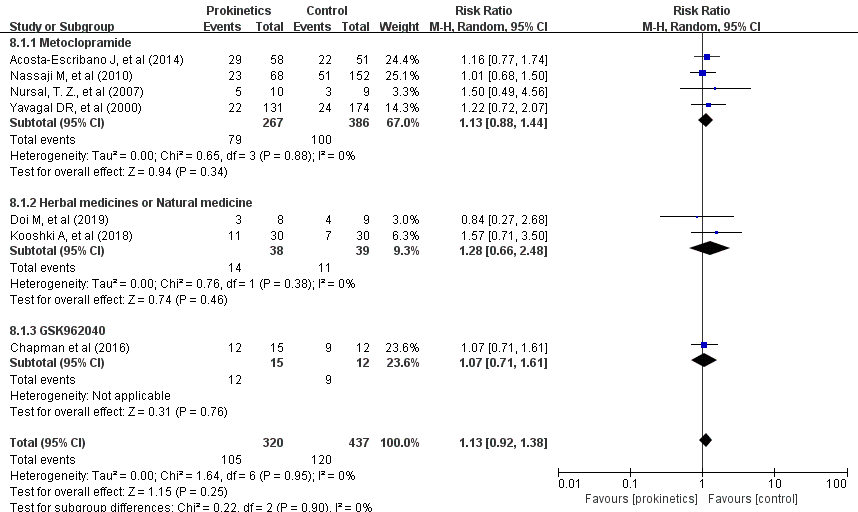
**

Supplement: S6 Fig — (DOCX) [file pone.0245317.s010.docx]

**S7 Fig. Subgroup analysis by the type of prokinetic agents for all-cause mortality outcomes**

**
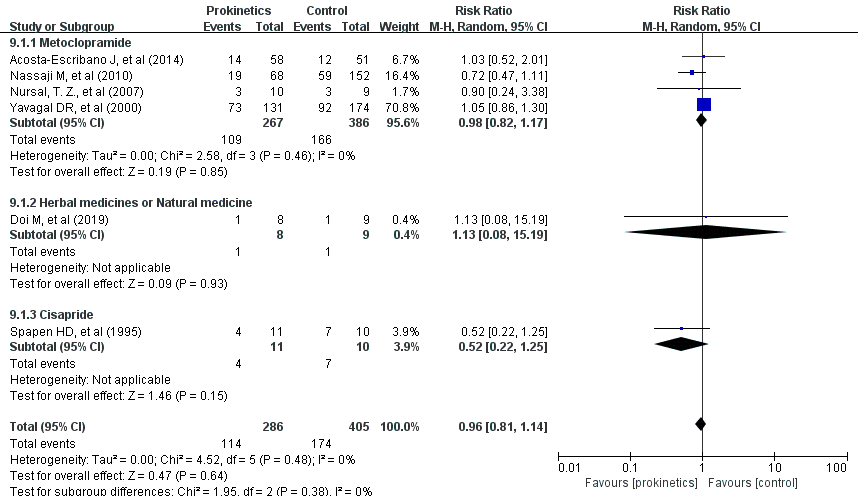
**

Supplement: S7 Fig — (DOCX) [file pone.0245317.s011.docx]

**S8 Fig. Sensitivity analysis of hospital length of stay outcomes**

**
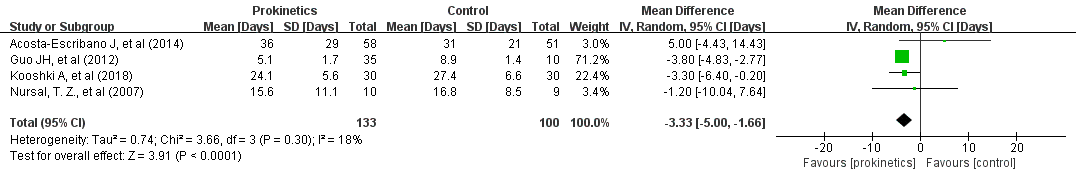
**

Supplement: S8 Fig — (DOCX) [file pone.0245317.s012.docx]

**S9 Fig. Sensitivity analysis of ICU length of stay outcomes**

**
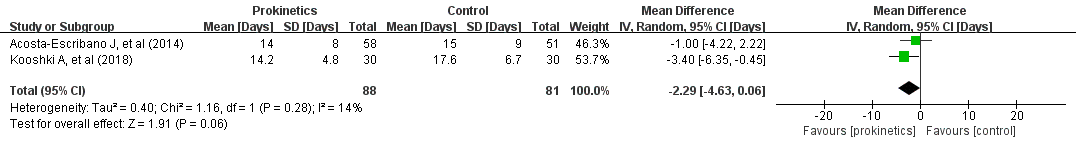
**

Supplement: S9 Fig — (DOCX) [file pone.0245317.s013.docx]

**S10 Fig. Sensitivity analysis of reported adverse event outcomes**

**
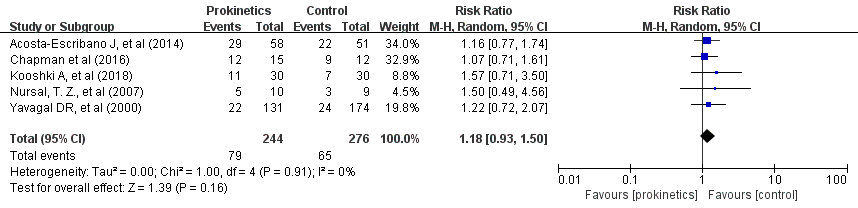
**

Supplement: S10 Fig — (DOCX) [file pone.0245317.s014.docx]

**S11 Fig. Sensitivity analysis of all-cause mortality outcomes**

**
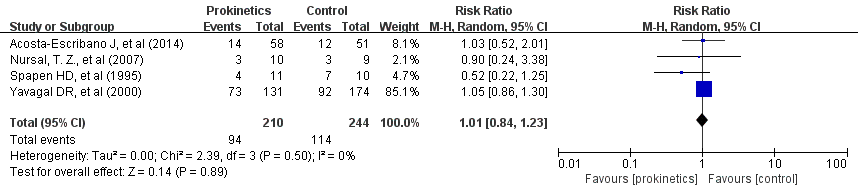
**

Supplement: S11 Fig — (DOCX) [file pone.0245317.s015.docx]
