# Supplementary material for: The efficacy and safety of prokinetics in critically ill adults receiving gastric feeding tubes: A systematic review and meta-analysis
Source: PLoS One. 2021 Jan 11;16(1):e0245317. doi: 10.1371/journal.pone.0245317 (PMC7799841; doi:10.1371/journal.pone.0245317)
Supplement: Attachment — Submitted filename: PONE-D-20-01167_R2_reviewer(1).pdf [file pone.0245317.s018.pdf]

PLOS ONE

The efficacy and safety of prokinetics in critically ill adults receiving gastric feeding tubes: A systematic review and meta-analysis

--Manuscript Draft--

**Manuscript Number:**	PONE-D-20-01167R2
**Article Type:**	Research Article
**Full Title:**	The efficacy and safety of prokinetics in critically ill adults receiving gastric feeding tubes: A systematic review and meta-analysis
**Short Title:**	Prokinetics for critically ill adults receiving gastric feeding tubes
**Corresponding Author:**	Lingli Zhang<br>Sichuan University<br>chengdu, Sichuan CHINA
**Keywords:**	Prokinetics; critical illness; Gastroparesis; enteral nutrition; systematic review
**Abstract:**	Background: Intolerance to gastric feeding tubes is common among critically ill adults and may increase morbidity. Administration of prokinetics in the ICU is common. However, the efficacy and safety of prokinetics are unclear in critically ill adults with gastric feeding tubes. We conducted a systematic review to determine the efficacy and safety of prokinetics for improving gastric feeding tube tolerance in critically ill adults.<br><br>Methods: Randomized controlled trials (RCTs) were identified by systematically searching the Medline, Cochrane and Embase databases. Two independent reviewers extracted relevant data and assessed the quality of the studies. We calculated pooled relative risks (RRs) for dichotomous outcomes and the mean differences (MDs) for continuous outcomes with the corresponding 95% confidence intervals (CIs). We assessed the risk of bias using the Cochrane risk-of-bias tool and used the Grading of Recommendations Assessment, Development, and Evaluation (GRADE) methodology to rate the quality of the evidence.<br><br>Results: Fifteen RCTs met the inclusion criteria. A total of 10 RCTs involving 846 participants were eligible for the quantitative analysis. Most studies (10 of 13, 76.92%) found that prokinetics showed beneficial effects on feeding intolerance in critically ill adults. In critically ill adults receiving gastric feeding, prokinetic agents may reduce the ICU length of stay (MD -2.03, 95% CI -3.96, -0.10; P = 0.04; low certainty) and the hospital length of stay (MD -3.21, 95%CI -5.35, -1.06; P = 0.003; low certainty). However, prokinetics failed to improve the outcomes of reported adverse events and all-cause mortality.<br><br>Conclusion: As a class of drugs, prokinetics may improve tolerance to gastric feeding to some extent in critically ill adults. However, the certainty of the evidence suggesting that prokinetics reduce the ICU or hospital length of stay is low. Prokinetics did not significantly decrease the risks of reported adverse events or all-cause mortality among critically ill adults.
**Order of Authors:**	Rong Peng
	Hailong Li
	Lijun Yang
	Linan Zeng
	Qiusha Yi
	Peipei Xu
	Xiangcheng Pan
	Lingli Zhang
**Response to Reviewers:**	Dear Editors and Reviewers,

Powered by Editorial Manager® and ProduXion Manager® from Aries Systems Corporation

Thank you for your letter and the comments concerning our manuscript (Manuscript Number: PONE-D-20-01167R1). We appreciate your positive comments regarding our manuscript. Your suggestions and ideas have been carefully considered. Revised portions are marked with changes in colored fonts in the paper. We hope that the revised manuscript will meet with your approval. The main corrections in the paper and our responses to the reviewers' comments are as follows:

Editors' comments:
The review is greatly improved in clarity and transparency, but requires some minor changes. Please address the comments of the reviewers and in addition:

1.For Line 288-289 where you state that hospital LOS was not significant I suggest you revise to use the same language as in 292-3 for ICU LOS, in that there appears to be a positive effect, unless you wish to clarify in what way the hospital LOS was not significant (clinically?)
Answer: We sincerely appreciate your thoughtful advice. This mistake was due to our carelessness in writing, and all authors sincerely apologize for this mistake. We have corrected this error. In the revised paper, the text is as follows: “These five studies, which enrolled a total of 250 patients, demonstrated a significant difference in the hospital length of stay between the prokinetic agent-treated group and the control group (MD -3.21, 95% CI -5.35, -1.06; P = 0.003; I2 = 28%) (Fig 2).”

2.For the outcomes of gastrointestinal symptoms and feeding tolerance, you should mention that if outcomes could not be combined by meta-analysis you summarized them narratively. You only discuss in the methods how you will use meta-analysis and then you do not meta-analyse the symptoms and tolerance outcomes, I assume because they are not appropriate to meta-analyze.
Answer: Yes. The various outcome definitions, especially for gastric tube tolerance, precluded quantitative synthesis of the data. According to your advice, we have amended this part in the revised manuscript as follows: “Thirteen studies evaluated the effect of prokinetics on gastrointestinal symptoms and/or feeding tolerance in adult critically ill patients receiving gastric feeding [26-30, 32-34, 36-40]. The main results obtained are as follows: gastric emptying, GRV, diarrhea, constipation, feeding complications and feeding intolerance. Gastric emptying was measured by the drug model of acetaminophen absorption or the 13C-octanoic acid breath test with calculation of the gastric emptying time, gastric emptying coefficient or area under the plasma concentration-time curve. The various outcome definitions, especially for gastric tube tolerance, precluded quantitative synthesis of the data.”

3.For Line 301 and the outcome of adverse events it is preferable to use the term 'risk' rather than 'incidence' as incidence implies measurement of time at risk.
Answer: Yes. Following your suggestion, to be more accurate, we have replaced “incidence” with “risk”.

4.For Table 3 please include the units for each outcome, e.g., days and deaths
Answer: Thank you for this comment. We have revised the manuscript to include the unit for each outcome in Table 3. We hope that this change improves the readability of the data.

5.For all forest plots, including Fig 2, Fig 3, S2Fig and S3Fig, please specify the comparison and the outcome (with units) in the header and replace the bracketed experimental and control on the x axis with a legend indicating the comparisons.
Answer: Following your suggestion, we have added “units” and “legend” to each forest plot. These changes have been made to the text to improve the readability and to clarify the interpretation of the data.

Reviewers' comments:

Reviewer #1:
1.I thank the authors for the significant work done to address al comments and i find the reviewed submission substantially improved.
Answer: Thank you for your very considerate advice; your positive comment on our manuscript is sincerely appreciated. We will reply to your comments one by one in detail.

2.the length of stay unit for hospital and ICU is still not clear. I assume it is days. However would be nice to have it clarified. See my original comment #11.
Answer: We apologize for this mistake. The unit for the lengths of hospital stay and ICU stay is “days”. We have added “units” in Table 3 in the revised manuscript. We hope that these changes improve the readability and clarify the interpretation of the data.

3.line 377: "We recommend a more comprehensive search and further original studies on this topic." i recommend the words "more comprehensive search" be deleted as they give the impression the authors did not perform a comprehensive search.
Answer: Thank you for your thoughtful reminder. We agree with your advice; the words "more comprehensive search" have been deleted in the revised manuscript.

Reviewer #3:
Thank you for the opportunity to review the revised manuscript. In this systematic review, authors evaluated the effect of prokinetics in critically ill adults on gastric feeding tube tolerance according to the updated definition. This systematic review implies that prokinetics improves tolerance of enteral feeding, and additionally provides the attractive hypothesis that prokinetics may shorten the length of ICU and hospital stay. Although authors tried to perform meta-analysis about gastric feeding tube tolerance, study diversity (e.g. various interventions and various outcome definitions) did not allow the authors data synthesis. Authors seems to revise their manuscript well according to the previous editor's and reviewers' comments.
Comments to the authors:
1.As authors state in background, the aim of this study is to evaluate the effect of prokinetics on gastric feeding tube tolerance. So, the main results of this study is the description about this effect (L273-283), not about ICU and hospital length of stay. And one of key points of this study, I believe, is the difficulty to compare results across previous studies because of various outcome definitions, and necessity of the valid measure of gastric tube tolerance in future studies. Authors should add more concise description in this paragraph (L273-283) to show the potential benefit on gastric feeding tube tolerance and clarify the abovementioned point.
Answer: Thank you for your thoughtful reminder. These comments are valuable and very helpful for revising and improving our paper and provided important guiding significance for our research. According to your advice, we have amended this part in the revised manuscript as follows:
“Thirteen studies evaluated the effect of prokinetics on gastrointestinal symptoms and/or feeding tolerance in adult critically ill patients receiving gastric feeding [26-30, 32-34, 36-40]. The main results obtained are as follows: gastric emptying, GRV, diarrhea, constipation, feeding complications and feeding intolerance. Gastric emptying was measured by the drug model of acetaminophen absorption or the 13C-octanoic acid breath test with calculation of the gastric emptying time, gastric emptying coefficient or area under the plasma concentration-time curve. The various outcome definitions, especially for gastric tube tolerance, precluded quantitative synthesis of the data.
As a class of drugs, prokinetic agents appear to have positive effects on gastrointestinal function and improving feeding tolerance. Ten of the thirteen studies reported positive effects on improving gastric emptying and/or resolution of feeding intolerance in critically ill patients with the use of prokinetic agents. However, two studies suggested that metoclopramide had no effect on decreasing gastrointestinal complications in adult neurocritical patients or critical traumatic brain injury patients. One study reported that rikkunshito did not improve the achievement of enteral calorie targets in critically ill adults (Table 2).”

2.L288-290: "Those five studies, enrolling a total of 250 patients, demonstrated that there was no significant difference in hospital length of stay ..."
Are there any significant difference between groups about the hospital length of stay? 95%CI of -5.35 to -1.06 is significant, isn't it? Please check.
Answer: I apologize for this mistake. We have corrected this error. In the revised manuscript, the text is as follows: “These five studies, which enrolled a total of 250 patients, demonstrated a significant difference in the hospital length of stay between the prokinetic agent-treated group and the control group (MD -3.21, 95% CI -5.35, -

	1.06; P = 0.003; I2 = 28%) (Fig 2)".<br><br>Thank you again for your attention and thoughtful advice. We hope that the revised manuscript will meet with your approval.
Additional Information:	
**Question**	**Response**
**Financial Disclosure**<br><br>Enter a financial disclosure statement that describes the sources of funding for the work included in this submission. Review the submission guidelines for detailed requirements. View published research articles from *PLOS ONE* for specific examples.<br><br>This statement is required for submission and **will appear in the published article** if the submission is accepted. Please make sure it is accurate.<br><br>**Unfunded studies**<br>Enter: *The author(s) received no specific funding for this work.*<br><br>**Funded studies**<br>Enter a statement with the following details:<br>• Initials of the authors who received each award<br>• Grant numbers awarded to each author<br>• The full name of each funder<br>• URL of each funder website<br>• Did the sponsors or funders play any role in the study design, data collection and analysis, decision to publish, or preparation of the manuscript?<br>• **NO** - Include this sentence at the end of your statement: *The funders had no role in study design, data collection and analysis, decision to publish, or preparation of the manuscript.*<br>• **YES** - Specify the role(s) played.<br><br>* typeset	The study was supported by National Major Science and Technology Projects of China (Award Number:<br>2017ZX09304029, Recipient: Lingli Zhang), Sichuan Province Science and Technology Major Project<br>(Award Number: 2017JY0067, Recipient: Lingli Zhang), the Major Project of Sichuan health<br>committee (Award Number: 18ZD042, Recipient: Lingli Zhang), the Major Project of Sichuan<br>Province Science and Technology in field of social development (Award Number: 20ZDYF3101,<br>Recipient: Lingli Zhang), Applied Basic Research Program of Science and Technology Department of<br>Sichuan province (Award Number: 20YYJC0072, Recipient: Rong Peng), the Project of Education<br>Department of Sichuan Province (Award Number: 18ZB0146, Recipient: Rong Peng). The funders had<br>no role in study design, data collection and analysis, decision to publish, or preparation of the<br>manuscript. We declare that we have no conflicts of interest.
**Competing Interests**<br><br>Use the instructions below to enter a competing interest statement for this submission. On behalf of all authors, disclose any competing interests that could be perceived to bias this work—acknowledging all financial support	The authors have declared that no competing interests exist.

and any other relevant financial or non-financial competing interests.<br><br>This statement **will appear in the published article** if the submission is accepted. Please make sure it is accurate. View published research articles from *PLOS ONE* for specific examples.<br><br>**NO authors have competing interests**<br><br>Enter: *The authors have declared that no competing interests exist.*<br><br>**Authors with competing interests**<br><br>Enter competing interest details beginning with this statement:<br><br>*I have read the journal's policy and the authors of this manuscript have the following competing interests: [insert competing interests here]*<br><br>* typeset	
**Ethics Statement**<br><br>Enter an ethics statement for this submission. This statement is required if the study involved:<br><br>• Human participants<br>• Human specimens or tissue<br>• Vertebrate animals or cephalopods<br>• Vertebrate embryos or tissues<br>• Field research<br><br>Write "N/A" if the submission does not require an ethics statement.<br><br>General guidance is provided below. Consult the submission guidelines for detailed instructions. **Make sure that all information entered here is included in the Methods section of the manuscript.**	N/A

**Format for specific study types**<br><br>**Human Subject Research (involving human participants and/or tissue)**<br>• Give the name of the institutional review board or ethics committee that approved the study<br>• Include the approval number and/or a statement indicating approval of this research<br>• Indicate the form of consent obtained (written/oral) or the reason that consent was not obtained (e.g. the data were analyzed anonymously)<br><br>**Animal Research (involving vertebrate animals, embryos or tissues)**<br>• Provide the name of the Institutional Animal Care and Use Committee (IACUC) or other relevant ethics board that reviewed the study protocol, and indicate whether they approved this research or granted a formal waiver of ethical approval<br>• Include an approval number if one was obtained<br>• If the study involved *non-human primates*, add *additional details* about animal welfare and steps taken to ameliorate suffering<br>• If anesthesia, euthanasia, or any kind of animal sacrifice is part of the study, include briefly which substances and/or methods were applied<br><br>**Field Research**<br><br>Include the following details if this study involves the collection of plant, animal, or other materials from a natural setting:<br>• Field permit number<br>• Name of the institution or relevant body that granted permission	
**Data Availability**<br><br>Authors are required to make all data underlying the findings described fully available, without restriction, and from the time of publication. PLOS allows rare exceptions to address legal and ethical concerns. See the PLOS Data Policy and FAQ for detailed information.	Yes - all data are fully available without restriction

A Data Availability Statement describing where the data can be found is required at submission. Your answers to this question constitute the Data Availability Statement and **will be published in the article**, if accepted.<br><br>**Important:** Stating 'data available on request from the author' is not sufficient. If your data are only available upon request, select 'No' for the first question and explain your exceptional situation in the text box.<br><br>Do the authors confirm that all data underlying the findings described in their manuscript are fully available without restriction?	
**Describe where the data may be found in full sentences. If you are copying our sample text, replace any instances of XXX with the appropriate details.**<br><br>• If the data are **held or will be held in a public repository**, include URLs, accession numbers or DOIs. If this information will only be available after acceptance, indicate this by ticking the box below. For example: *All XXX files are available from the XXX database (accession number(s) XXX, XXX.*).<br>• If the data are all contained **within the manuscript and/or Supporting Information files**, enter the following: *All relevant data are within the manuscript and its Supporting Information files.*<br>• If neither of these applies but you are able to provide **details of access elsewhere**, with or without limitations, please do so. For example:<br><br>*Data cannot be shared publicly because of [XXX]. Data are available from the XXX Institutional Data Access / Ethics Committee (contact via XXX) for researchers who meet the criteria for access to confidential data.*<br><br>*The data underlying the results presented in the study are available from (include the name of the third party*	All data generated or analysed during this study are included in this published article.

and contact information or URL).<br>• This text is appropriate if the data are owned by a third party and authors do not have permission to share the data.<br><br>* typeset	
Additional data availability information:	

Manuscript

Click here to access/download;Manuscript;Manuscript_Final.doc

The efficacy and safety of prokinetics in critically ill adults receiving gastric feeding tubes: A systematic review and meta-analysis

Rong Peng[1,2,3,4], Hailong Li [1,2,3], Lijun Yang [5], Linan Zeng [1,2,3], Qiusha Yi [1,2,3], Peipei Xu [1,2,3], Xiangcheng Pan [1,2,3], Lingli Zhang [1,2,3*]

[1] Department of Pharmacy, West China Second University Hospital, Sichuan University, Chengdu, Sichuan, China

[2] Evidence-Based Pharmacy Center, West China Second University Hospital, Sichuan University, Chengdu, Sichuan, China

[3] Key Laboratory of Birth Defects and Related Diseases of Women and Children (Sichuan University), Ministry of Education, Chengdu, Sichuan, China

[4] Department of Clinical Nutrition, Affiliated Hospital of Chengdu University, Chengdu, Sichuan, China

[5] Department of General Practice Medicine, Affiliated Hospital of Chengdu University, Chengdu, Sichuan, China

*Corresponding Author: Lingli Zhang, Department of Pharmacy, West China Second University Hospital, Sichuan University. Renming Road, Chengdu, Sichuan, 610041, China. Tel: 0862885502378

E-mail: zhanglingli@scu.edu.cn

Abstract

Background: Intolerance to gastric feeding tubes is common among critically ill adults and may increase morbidity. Administration of prokinetics in the ICU is common. However, the efficacy and safety of prokinetics are unclear in critically ill adults with gastric feeding tubes. We conducted a systematic review to determine the efficacy and safety of prokinetics for improving gastric feeding tube tolerance in critically ill adults.

Methods: Randomized controlled trials (RCTs) were identified by systematically searching the Medline, Cochrane and Embase databases. Two independent reviewers extracted relevant data and assessed the quality of the studies. We calculated pooled relative risks (RRs) for dichotomous outcomes and the mean differences (MDs) for continuous outcomes with the corresponding 95% confidence intervals (CIs). We assessed the risk of bias using the Cochrane risk-of-bias tool and used the Grading of Recommendations Assessment, Development, and Evaluation (GRADE) methodology to rate the quality of the evidence.

Results: Fifteen RCTs met the inclusion criteria. A total of 10 RCTs involving 846 participants were eligible for the quantitative analysis. Most studies (10 of 13, 76.92%) found that prokinetics showed beneficial effects on feeding intolerance in critically ill adults. In critically ill adults receiving gastric feeding, prokinetic agents may reduce the ICU length of stay (MD -2.03, 95% CI -3.96, -0.10; P = 0.04; low certainty) and the hospital length of stay (MD -3.21, 95%CI -5.35, -1.06; P = 0.003; low certainty). However, prokinetics failed to improve the outcomes of reported adverse events and all-cause mortality.

Conclusion: As a class of drugs, prokinetics may improve tolerance to gastric feeding to some extent in critically ill adults. However, the certainty of the evidence suggesting that prokinetics reduce the ICU or hospital length of stay is low. Prokinetics did not significantly decrease the risks of reported adverse events or all-cause mortality among critically ill adults.

Keywords: Prokinetics; Critical illness; Gastroparesis; Enteral nutrition; Systematic review.

Introduction

Critical illness is usually associated with catabolic stress and increases the incidence of infection and multiple organ dysfunction, bringing disproportionately high mortality. A systematic review found a strikingly high prevalence of malnutrition in intensive care unit (ICU) patients (ranging from 38% to 78%) [1]. Owing to the benefits of nutrition support in reducing disease severity and favorably impacting patient outcomes, early nutrition support therapy, primarily by the enteral route, is seen as a proactive therapeutic strategy [2]. In addition, if oral intake is not possible, tube feeding through gastric access has been recommended as the standard approach to initiate enteral nutrition in adult critically ill patients [3], as gastric feeding provides greater nutritional and non-nutritional benefits than parenteral nutrition and is more likely to improve the prognosis [4]. Furthermore, gastric feeding is more physiological than postpyloric feeding [illegible] does not require a higher level of technology [3].

However, enteral tube feeding intolerance occurs frequently in critically ill patients. Blaser et al. reported that the pooled proportion of feeding intolerance was 38.3% (95% confidence interval (CI) 30.7–46.2%) [5]. Feeding intolerance was first described by the Working Group on Abdominal Problems of the European Society of Intensive Care Medicine as failure to provide adequate enteral nutrition to critically ill patients for clinical reasons (vomiting, high gastric residual volume (GRV), diarrhea, gastrointestinal bleeding, presence of entero-cutaneous fistulas, etc.); if a feeding rate of at least 20 kcal/kg body weight (BW)/day cannot be reached via the enteral route in 72 h of feeding attempts or if enteral feeding must be stopped for any clinical reason, feeding intolerance should be considered present [6]. However, feeding intolerance is inconsistently defined in different studies, with the definitions falling into three main categories: (1) "large" GRV; (2) presence of gastrointestinal symptoms; or 3) inadequate delivery of enteral nutrition [5]. Feeding intolerance is associated with increasing mortality, and seven-day feeding intolerance is an independent predictor of 60-day mortality [7]. In addition, a meta-analysis by the European Society for Clinical Nutrition and Metabolism showed that gastric feeding intolerance was more prevalent than postpyloric feeding

intolerance (25.7% vs. 3.5%, p=0.0005) [3]. Given the risk associated with gastric feeding intolerance, it should be treated aggressively.

There are three methods for the treatment of gastric feeding intolerance. First, there is the most widely used method, the administration of prokinetics. Among recipients of gastric feeding, 13% had been prescribed prokinetics preemptively before they developed intolerance. Approximately one-third of patients who developed feeding intolerance were treated with a prokinetic agent during their stay in the ICU. Second, after the development of intolerance, 17% of patients received supplemental parenteral nutrition. Third, only 7.5% of patients with gastric feeding intolerance subsequently received enteral nutrition via a postpyloric feeding tube [8]. Although the use of prokinetics in the ICU is common, the recommendations vary from one authority to another. For example, the ESPEN guidelines on clinical nutrition in the ICU [3] suggest that intravenous erythromycin should be used as a first-line prokinetic therapy in critically ill patients with gastric feeding intolerance (grade of recommendation: B – strong consensus, 100% agreement). Alternatively, intravenous metoclopramide or a combination of metoclopramide and erythromycin can be used as a prokinetic therapy (Grade of recommendation: 0 – strong consensus, 100% agreement). However, the ASPEN/SCCM guidelines weakly recommend the use of gastrointestinal motility agents in the case of feeding intolerance (low quality of evidence) [2]. The Canadian Critical Care Clinical Practice guidelines recommend metoclopramide as the first-line prokinetic agent in the ICU [9]. However, in Chinese guidelines, herbal or natural medicines that enhance gastric motility are recommended for patients receiving gastric feeding tubes [10]. There is little agreement on how to use prokinetics for gastric feeding intolerance in critically ill patients. One of the reasons for the different recommendations may be that the definition of feeding intolerance has changed over time, especially regarding the index of high GRV. ~~Some studies have suggested that measurement of GRV provides no benefit and should no longer be recommended. However, GRV is also an indicator of feeding intolerance in many ICUs~~, especially in patients with a high risk of aspiration and aspiration pneumonia. Therefore, the Chinese guidelines call for caution in abandoning monitoring of GRV in some high-risk patients [10]. If GRV

is measured, a volume of less than 500 mL should not result in an interruption of feeding unless there are other signs of intolerance, such as nausea, vomiting, abdominal pain, abdominal distension, or deterioration in hemodynamics or overall status [11]. A GRV of 500 mL is the recommended threshold for a diagnosis of enteral feeding intolerance in US and European critical care and nutrition society guidelines [2, 3, 12]. Although the updated European Society for Clinical Nutrition and Metabolism (ESPEN) guidelines [3], published in 2019, provide the latest information on enteral nutrition (EN) and parenteral nutrition (PN) in critically ill adult patients, we find that some aspects of the efficacy and safety of prokinetics in critically ill patients are still quite unclear [13], and it is necessary to find new evidence to address these uncertainties.

On this topic, a previous meta-analysis by Lewis, K. et al. [14] examined the effects of prokinetics on feeding intolerance or high GRV and clinical outcomes. However, Lewis, K. et al. [14] defined feeding intolerance as GRV ≥150 mL, vomiting, or abdominal distention resulting in feeding interruption. This definition may be considered obsolete [15]. Some new evidence has emerged on this topic; considering recent evidence, we conducted this systematic review to determine the efficacy and safety of prokinetics for intolerance of gastric feeding in critically ill adult patients.

Methods

This systematic review and meta-analysis was conducted according to the Cochrane Handbook for Systematic Reviews of Interventions (version 5.1.0) [16], and the reporting of our study was based on the Preferred Reporting Items for Systematic Reviews and Meta-analyses (PRISMA) statement [17]. The review protocol is available on PROSPERO, registration number CRD42020157446.
Neither patients who received gastric feeding in the ICU nor their families were involved in defining the research question or the outcome measures, but they were intimately involved in the design, giving our team a wealth of good advice regarding design ideas.

Search strategy

We searched the Medline and Embase databases as well as the Cochrane Central Register of Controlled Trials (CENTRAL) from their inception dates to November 22, 2019. We combined Medical Subject Headings (MeSH) and free text terms to identify relevant articles. An information expert (XY) developed our search strategies.

We also searched clinicaltrials.gov (https://clinicaltrials.gov/) and the WHO ICTRP (http://apps.who.int/trialsearch/) for additional information, using the terms "critically ill patients", and limited our search to studies labeled "completed" AND "Interventional studies (clinical trials)" in which summary results were available to identify additional eligible studies. There were no language restrictions. Additionally, we used a manual search strategy to retrieve the relevant articles referred to by the retrieved publications (the search strategies are reported in **S1 Table**).

Inclusion criteria

Trials were selected based on the following inclusion criteria: (1) the study was designed as a randomized controlled trial (RCT) comparing prokinetic treatment with a control group; (2) the population included critically ill adult patients aged ≥18 years who were admitted to the ICU and received gastric feeding tubes regardless of whether they had pre-existing feeding intolerance; (3) the intervention group received metoclopramide, erythromycin, or other prokinetic agents, such as herbal medicine or natural medicines with the function of enhancing gastric motility, regardless of the dose, frequency, duration or combination of prokinetics; (4) the control group received no intervention or a placebo; (5) if the gastric feeding patients with feeding intolerance had a GRV ≥500 mL and/or symptoms of nausea, vomiting, abdominal distention, regurgitation, deterioration in hemodynamics or other symptoms resulting in feeding interruption and failed to respond to interventions, regardless of whether they were in the control group or the prokinetics group, they were switched to postpyloric feeding or had gastric feeding withheld for 4-6 h [2, 3]; and (6) the outcomes included any of the

following: all-cause mortality; Acute Physiology and Chronic Health Evaluation II (APACHE II) or Simplified Acute Physiology Score II; sepsis; use of an artificial airway; pneumonia; hospital or ICU length of stay; patient nutritional status (malnutrition); gastrointestinal symptoms; GRV; feeding intolerance; or side effects of the prokinetics, such as cardiovascular disorders, bronchospasm, extrapyramidal symptoms, abdominal cramps, allergic reactions and pancreas disorders. The exclusion criteria were as follows: (1) the studies had no control group; (2) the studies had no prokinetic treatment group; (3) patients were considered to have feeding intolerance if tube feeding was electively not prescribed or was stopped/interrupted for procedural reasons; (4) the studies discontinued or interrupted the gastric feeding prematurely when the GRV was less than 500 mL or the patients did not have any signs of intolerance, such as nausea, vomiting, abdominal pain, abdominal distension, or deterioration in hemodynamics or overall status.

For our purposes, gastric feeding intolerance was defined as a “large” GRV (≥500 mL), the presence of gastrointestinal symptoms (vomiting, diarrhea, gastrointestinal bleeding, the presence of enterocutaneous fistulas), or inadequate delivery of EN (the energy provided by EN was less than 20 kcal/kg BW/day after 72 h of feeding attempts or less than 60% of the EN target at the fifth day) in critically ill adults receiving gastric feeding tubes. Preventive usage of prokinetics meant that prokinetics were prescribed preemptively on the day EN was initiated and before patients presented a GRV >150 mL or symptoms of feeding intolerance. Preventive usage of prokinetics for risk meant that prokinetics were used in patients with GRVs between 150 and 500 mL but before the development of intolerance. Therapeutic usage of prokinetics meant that the prokinetics were administered in patients who had developed feeding intolerance.

A reported adverse event was defined as any untoward medical occurrence or unfavorable and unintended sign, including an abnormal laboratory finding, symptom, or disease (new or exacerbated), temporally associated with the use of the study medication. The reported adverse events included abnormal laboratory test results (hematology, clinical chemistry, or urinalysis) or other safety assessments (e.g., ECGs, radiological scans, or measurements of vital signs), including those that

worsened from baseline and were deemed clinically significant in the medical and scientific judgment of the investigator; exacerbation of a chronic or intermittent preexisting condition, including an increase in the frequency and/or intensity of the condition; new conditions detected or diagnosed after the administration of study medication even if they may have been present prior to the start of the study; and/or signs, symptoms, or clinical sequelae of a suspected interaction, such as diarrhea, nosocomial pneumonia, severe sepsis, brain herniation, cardiac arrest, or changes in the electrocardiographic QTc interval.

Risk-of-bias assessments

The methodological quality for the included RCTs was assessed independently by 2 researchers (RP, HLL) based on the Cochrane risk-of-bias criteria [16]. The seven items used to evaluate bias in each trial included randomization sequence generation, allocation concealment, blinding of participants and personnel, blinding of outcome assessment, incomplete outcome data, selective reporting, and other bias. We defined other bias as being present in the trials where baseline characteristics were not similar between different intervention groups. The included trials were graded as low quality, high quality, or unclear risk based on the following criteria: (1) trials were considered low quality if either randomization or allocation concealment was assessed as having a high risk of bias, regardless of the risk of other items; (2) trials were considered high quality when both randomization and allocation concealment were assessed as having a low risk of bias and all other items were assessed as having a low or unclear risk of bias; (3) trials were considered to have unclear risk if they did not meet the criteria for high or low risk.

Data extraction

Two researchers (RP, HLL) independently extracted the following information from each eligible RCT: (1) general study characteristics: author name, year of publication, numbers of treatment groups and patients, trial registry number, methods for measuring gastric emptying or GRV, and the definition

of feeding intolerance; (2) patient characteristics: sex, age, baseline patient information (presence or absence of pre-existing feeding intolerance, APACHE II score and nutritional status, if reported); (3) primary diseases (the medical, surgical, or neurosurgical conditions of the critically ill patients); (4) interventions: details of the prokinetic treatment group and control group (e.g., dose, frequency, duration and combination of prokinetics for treatment); and (5) outcomes: gastrointestinal symptoms, feeding tolerance, the number of participants with all-cause death, the ICU length of stay, the hospital length of stay, and the number of reported adverse events.

If the trials had more than 2 groups or used factorial designs and could be analyzed using multiple comparisons, we extracted only the information and data of interest reported in the original articles. If a trial had multiple reports, we collated all data into one study. If a trial had both reports from ClinicalTrials.gov and journal publications, we carefully checked data from these two sources for consistency. If outcome data were reported at multiple follow-up points, we used data from the longest follow-up.

Statistical analysis

The effect of prokinetics on gastrointestinal symptoms and feeding tolerance, main clinical outcomes of all-cause mortality, ICU length of stay, hospital length of stay, and reported adverse events were analyzed. We recorded data on the number of participants with each outcome event by allocated group and recorded the number of participants with compliance and the participant, who was later thought to be eligible or otherwise excluded from treatment or follow-up. Intention-to-treat (ITT) analysis was conducted. ITT analysis is a comparison of the treatment groups that include all patients as originally allocated after randomization regardless of whether treatment was initiated or completed [18]. The CONSORT (Consolidated Standards of Reporting Trials) recommends ITT analysis as standard practice [19].

We performed a meta-analysis to calculate relative risks (RRs) or absolute risk differences (ARDs) in

dichotomous data and mean differences (MDs) in continuous data, 95% CIs using the Mantel-Haenszel method and the inverse variance statistical method, respectively. If sufficient data were not available in the published reports or the abstract of the conference, we contacted the authors of the paper. If the raw data were not the mean and standard deviation, the sample mean and standard deviation were estimated from the sample size, median, range and/or interquartile range [20, 21].

We tested for heterogeneity between trial results using a standard Chi^2 test, and statistical heterogeneity between summary data was evaluated using the I^2 statistic. Sensitivity analysis was performed by excluding low-quality studies, trials recruiting participants with particular conditions, or trials with characteristics different from the others. When an inconsistency was detected between the RR and ARD for the same outcome, we explained the results based on the RR because the RR model is more consistent than ARD, particularly for an intervention aimed at preventing an undesirable event [16, 22].

In our meta-analysis, a random-effects model was used. The defining feature of the random-effects model is that there is a distribution of true effect sizes, and there are two sources of variance, within-study error variance and between-study variance [23]. However, if the number of studies is very small, the statistical power will have poor precision due to the variance between studies. Although the random-effects model is still the appropriate model, the information to apply it correctly is not available. In this case, we will add the separate effects to our manuscript. If heterogeneity was identified (I^2 >40% [16]) and sufficient trials were included in the review, we planned to investigate heterogeneity in the specified subgroups based on types of prokinetics (erythromycin, metoclopramide or other prokinetics), combination of prokinetics (yes or no), and feeding intolerance history (participants with or without pre-existing feeding intolerance before the start of the trial). Analysis was performed to assess whether the difference between the subgroups was statistically significant.

We assessed publication bias by examining funnel plots when the number of trials reporting the primary outcomes was 10 or more. However, if the number of included studies is less than 10 for a

given main outcome, the funnel plot may not reliably detect evidence of departure bias. A prototypical situation that should elicit suspicion of publication bias is when evidence is derived from a small number of studies or small sample sizes and all outcomes favor the intervention [24]. All meta-analyses were performed using RevMan version 5.3 (Cochrane Collaboration). All tests were 2-tailed, and $P < 0.05$ was considered statistically significant.

We used the Grading of Recommendations Assessment, Development, and Evaluation (GRADE) methodology to rate the certainty of evidence as high, moderate, low, or very low. RCTs begin as high-certainty evidence but can downgraded because of risk of bias, imprecision, inconsistency, indirectness, or publication bias. If the limitation of the evidence was considered serious, the evidence was downgraded by one level; if the limitation was considered very serious, the evidence was downgraded by two levels [25].

Results

Our initial search identified a total of 595 citations. After deduplication, 459 publications remained. The titles and abstracts of those records were screened for inclusion, and 48 reports proved potentially eligible. After full-text screening, fifteen trials met the inclusion criteria [26-40]. Five studies did not provide useful data for quantitative synthesis (meta-analysis) [36-40]. Ultimately, 10 trials were included in the quantitative analysis [26-35]. A total of 846 patients were enrolled in 10 RCTs, including a variety of critically ill patients with medical, surgical, and neurosurgical conditions. The details of the eligible trials are presented in **Fig 1**. Studies were excluded if they had a different trial design [41-45], a different intervention or a different control [46-64], or a different population [65-67] or had been registered with the Clinical Trials Registry Platform (clinicaltrials.gov or WHO ICTRP) and had been labeled "completed" but outcomes were not reported [68-73] (**S2 Table**).

Fig 1. Literature Search and Screening Process.

The 15 eligible studies reported 10 prokinetics, including metoclopramide, erythromycin, cisapride, GSK962040, mosapride and herbal medicine or natural medicines with the function of enhancing gastric motility (Chenxia Sijunzi decoction, ginger, fenugreek seed powder, gastrolit (*Zataria multiflora*), rikkunshito), respectively. Based on the outcomes measured, the studies were subdivided into those investigating effects on gastrointestinal symptoms, feeding tolerance studies, and clinical outcome studies: hospital length of stay, ICU length of stay, reported adverse events, and all-cause mortality. The details of the eligible studies are presented in **Table 1**.

Table 1. Characteristics of the included trials and participants

Included Trials	Population	Treatment #	Main outcomes	Definition of feeding intolerance †	Prokinetic initiation timing *
Yavagal et al 2000 (India) [35]	ICU patients required placement of a nasogastric tube for >24 hrs. Mean age: 36.22 years, 61.97% male. Mean APACHE II score: 17.54.	1) Metoclopramide 10 mg NG q8h;<br>2) Placebo.	1) Nosocomial pneumonia;<br>2) Mortality.	NA	Preventive usage
Sustic et al 2005 (Croatia) [39] ¶	Patients treated at a cardiosurgical ICU after CABG surgery, enteral feeding by nasogastric tube. Mean age: 59.5 years, 77.5% male. Mean SAPS II score: 21.	1) Metoclopramide 10 mg i.v.;<br>2) Control group.	1) t_{+15}, t_{+30}, t_{+60}, t_{+120};<br>2) AUC_{120};<br>3) $C_{max.}$	NA	Preventive usage
Nursal et al 2007 (Turkey) [32]	Traumatic brain injury patients with Glasgow Coma Scale scores of 3–11. Enteral feeding by nasogastric tube. Mean age: 43.42 years, 84.2% male Mean APACHE II score: 12.87.	1) Metoclopramide 10 mg i.v. q8h×5 days;<br>2) Control group, saline	1) Feeding intolerance;<br>2) Feeding complications;<br>3) $AUC_{60;}$<br>4) C_{max};<br>5) Length of hospital stay;<br>6) Mortality.	Gastrointestinal symptoms (without GRV)	Preventive usage
Nassaji et al 2010 (Islamic Republic of lran) [31]	Surgical ICU with a nasogastric tube for more than 24 hours. Mean age: 44.88 years, 65.45% male. Mean APACHE II score: not reported.	1) Metoclopramide 10 mg NG q8h;<br>2) Control patients did not receive metoclopramide.	1) Nosocomial pneumonia;<br>2) Mortality.	NA	Preventive usage

Acosta-Escribano et al 2014 (Spain) [26]	Adult neuro-critical patients, Glasgow Coma Scores of 14 to 9 points, with ventilation indications at admission and the need for artificial enteral nutrition. Mean age: 54.53 years, 65.14% male. Mean APACHE II score: 18.53.	1) Metoclopramide 10 mg;<br>2) Placebo.	1) Gastrointestinal complications;<br>2) Gastric residue;<br>3) Mechanical ventilation-associated pneumonia;<br>4) The duration of mechanical ventilation;<br>5) Length of ICU stay;<br>6) Length of hospital stay;<br>7) Mortality.	Large GRV alone (>500 mL in two consecutive episodes)	Preventive usage
Rajan et al 2017 (India) [38] ¶	Critically ill cirrhotic patients in a liver ICU with feeding intolerance.	1) Metoclopramide i.v.,<br>2) Erythromycin i.v.,<br>3) Placebo.	1) Mortality;<br>2) GRV.	Gastrointestinal symptoms including large GRV (500 mL)	Therapeutic usage
Ritz et al 2005 (Australia) [33]	Mixed medical/surgical intensive care unit patients with mechanic ventilation. Mean age: 47.49 years, 60.9% male. Mean APACHE II score: 19.	1) **Erythromycin 70 mg;**<br>2) Erythromycin 200 mg;<br>3) Placebo, saline (0.9%).	1) Gastric emptying coefficient;<br>2) Gastric half-emptying time ($t_{1/2}$).	NA	Preventive usage
Spapen et al 1995 (Belgium) [34]	Adult medical/surgical intensive care unit patients requiring prolonged mechanical ventilation and enteral feeding. Mean age: 71.10 years, 52.38% male. Mean APACHE II score: not reported.	1) Cisapride 10 mg q6h;<br>2) No treatment.	1) Gastric residue;<br>2) The mean time at which 50% of the technetium 99m-labeled test meal was eliminated from the stomach ($T_{1/2}$);<br>3) Mortality.	NA	Preventive usage

Heyland et al 1996 (Canada) [36] ¶	Mechanically ventilated patients in trauma and neurosurgery ICUs. Mean age: 53.9 years, 61% male. Mean SAPS score: 9.5.	1) Cisapride 20 mg, NG;<br>2) An identical placebo.	1) C_{max};<br>2) AUC_{180}.	NA	Preventive usage
Chapman et al, 2016 (Australia) [27]	Patients undergoing invasive mechanical ventilation in the ICU with nasogastric feeding. Mean age: 44.67 years, 83.33% male. Mean APACHE II score: 18.14.	**1) GSK962040 (50 mg);**<br>2) GSK962040 (75 mg);<br>3) Placebo.	1) Breath test gastric time to half emptying (BTt½);<br>2) Gastric emptying coefficient;<br>3) AUC_{240}, AUC_{60};<br>4) C_{max};<br>5) Adverse events.	Large GRV alone (>200 mL) at least 6 hours after commencing liquid nutrition at ≥ 40 kcal/hr	Preventive usage for risk
Mokhtari et al 2009 (Islamic Republic of lran) [37] ¶	Adult respiratory distress syndrome (ARDS) ICU patients.	1) Ginger,<br>2) Placebo.	1) Feeding tolerated;<br>2) Ventilator-associated pneumonia;<br>3) ICU-free days;<br>4) Ventilator-free days;<br>5) Morality.	Delayed gastric emptying is one of the major reasons for enteral feeding intolerance	Preventive usage
Guo JH, et al 2012 (China) [29]	Feeding with enteral nutrition in critically ill patients. Mean age: 59.49 years, 53.33% male. Mean APACHE II score: not reported.	1) **Traditional Chinese medicine group: Chenxia Sijunzi decoction**;<br>2) Western medicine group: mosapride dispersible tablets 5 mg and multienzyme tablets NG;<br>3) Control group: routine symptomatic treatment without any medicines to promote gastrointestinal function.	1) The time of bowel sound recovery;<br>2) Gas passage time by anus;<br>3) The bowel movement time;<br>4) Days in the hospital.	NA	Preventive usage

Kooshki et al 2018 (Iran) [30]	Mechanically ventilated patients, enteral nutrition with nasogastric tube in two intensive care unit centers. Mean age: 56.95 years, 51.67% male. Mean APACHE II score: 23.2.	1) Fenugreek seed powder 3 g q12h NG;<br>2) Routine care.	1) Diarrhea;<br>2) Constipation;<br>3) GRV;<br>4) Respiratory aspiration;<br>5) Duration of mechanical ventilation;<br>6) Length of stay in the hospital;<br>7) Length of stay in the ICU;<br>8) Mortality.	Gastrointestinal symptoms	Preventive usage
Tahershamsi et al 2018 (Iran) [40] ¶	Mechanically ventilated patients hospitalized in ICU. Mean age: 63.06 years, 60.0% male. Mean APACHE II score: No report.	1) Gastrolit (*Zataria multiflora*) (20 drops) q8h× 4 days;<br>2) Placebo = water.	1) GRV.	NA	Preventive usage
Doi et al 2019 (Japan) [28]	Critically ill adult patients requiring enteral nutrition by gastric tube for at least 5 days, and all patients were treated with invasive mechanical ventilation. Mean age: 72.84 years, 77.78% male. Mean APACHE II score: 22.82.	**1) Rikkunshito 5 g** q8h × 5 days;<br>2) Rikkunshito 2.5 g q8h× 5 days;<br>3) No rikkunshito (control).	1) GRV;<br>2) The percentage of the target enteral calorie intake achieved at the fifth day;<br>3) The plasma levels of ghrelin;<br>4) ICU length of stay;<br>5) Hospital length of stay;<br>6) Adverse events;<br>7) Mortality.	Inadequate enteral nutrition/failure to meet the enteral nutrition target at the fifth day (<60%)	Preventive usage

NG: nasogastric tube feeding; i.v.: intravenous injection; NA: not applicable; C_{max}: peak paracetamol plasma levels; AUC: the area under the paracetamol concentration curve; t_{+15}, t_{+30}, t_{+60}, t_{+120}: plasma paracetamol concentrations at 15, 30, 60, and 120 minutes after administration of paracetamol and saline or metoclopramide in patients; SAPS, simplified acute physiology score; GRV, gastric residual volume.

# If the trials had more than 2 groups or factorial designs and permitted multiple comparisons, the subgroup in bold font was extracted in this study.

¶ The study did not provide useful data for meta-analysis.

Risk of bias

There was one trial at low risk of bias [27], and two studies were at high risk of bias [28, 31] due to inappropriate randomization and/or allocation concealment. For the remaining 12 studies, we were unable to comprehensively evaluate the risk of bias due to a lack of information [26, 29, 30, 32-40]. **(S1 Fig)**.

Publication bias

We checked the funnel plots of the main outcomes for asymmetry as planned; however, we included less than 10 RCTs in each main outcome, such that the funnel plots may not reliably detect evidence of departure bias.

Main outcomes

Effect on gastrointestinal symptoms and feeding tolerance

Thirteen studies evaluated the effect of prokinetics on gastrointestinal symptoms and/or feeding tolerance in adult critically ill patients receiving gastric feeding [26-30, 32-34, 36-40].
The main results obtained are as follows: gastric emptying, GRV, diarrhea, constipation, feeding complications and feeding intolerance. Gastric emptying was measured by the drug model of acetaminophen absorption or the 13C-octanoic acid breath test with calculation of the gastric emptying time, gastric emptying coefficient or area under the plasma concentration-time curve. The various outcome definitions, especially for gastric tube tolerance, precluded quantitative synthesis of the data.

As a class of drugs, prokinetic agents appear to have positive effects on gastrointestinal function and improving feeding tolerance. Ten of the thirteen studies reported positive effects on improving gastric emptying and/or resolution of feeding intolerance in critically ill patients with the use of prokinetic agents. However, two studies suggested that metoclopramide had no effect on decreasing

gastrointestinal complications in adult neurocritical patients or critical traumatic brain injury patients. One study reported that rikkunshito did not improve the achievement of enteral calorie targets in critically ill adults **(Table 2)**.

Table 2. Effects on gastrointestinal symptoms and feeding tolerance

Study	Population (sample size)	Intervention	Outcome	*P* Value	Conclusions
Sustic et al 2005 (Croatia) [39]	Cardiosurgical patients after CABG surgery (40)	1) Metoclopramide 10 mg i.v.;<br>2) Control group: saline.	AUC_{120}; $C_{max.}$<br>574±296; 8.51±2.2<br>429±309; 5.15±2.8	0.027; 0.007	In CABG surgery patients with early enteral feeding, a single dose of intravenous metoclopramide effectively improves gastric emptying.
Nursal et al 2007 (Turkey) [32]	Traumatic brain injury patients with Glasgow Coma Scores of 3–11 (19)	1) Metoclopramide 10 mg i.v. q8h×5 days;<br>2) Control group: saline.	FI; feeding complications; AUC_{60} at day 5; C_{max} day 5;<br>4/10 (40%); 5/10 (50%); 589.6±457.8; 15.8±12.9<br>2/9 (22.2%); 3/9 (33.3%); 560±432.9; 12.0±9.9	NS; NS; NS; NS	The results were unable to reveal any advantage of using metoclopramide in TBI patients.
Acosta-Escribano et al 2014 (Spain) [26]	Adult neuro-critical patients, Glasgow Coma Scores of 14 to 9 points (109)	1) Metoclopramide 10 mg i.v.;<br>2) Placebo: saline.	Incidence of gastrointestinal complications; Incidence of GRV>500 mL at day 5;<br>29/58 (50%); 16/58 (28%)<br>22/51 (45%); 11/51 (22%)	NS; NS	Metoclopramide has no effect on decreasing gastrointestinal complications in adult neuro-critical patients

Rajan et al 2017 (India) [38]	Critically ill cirrhotic patients in a liver intensive care unit (72)	1) Metoclopramide i.v.;<br>2) Erythromycin i.v.;<br>3) Placebo.	Resolution of FI; Decrease in GRV beyond 24 hrs; the time to restart enteral nutrition (days)<br>8.7%; no report; 2.61±0.72<br>24%; no report; 2.20±0.91<br>no report; no report; 3.47±1.29	0.026; no report; 0.03	Early detection and the addition of prokinetics facilitate the resolution of FI in critically ill cirrhotic patients. Erythromycin is safe and superior to metoclopramide for early resolution of gut paralysis in critically ill cirrhotic patients.
Ritz et al 2005 (Australia) [33]	Mixed medical/surgical intensive care unit patients (35)	1) **Erythromycin 70 mg i.v.;**<br>2) Erythromycin 200 mg i.v.;<br>3) Placebo, saline (0.9%).	Gastric emptying coefficient; gastric half-emptying time ($t_{1/2}$)<br>3.8 (3.3-4.0); 98 (88-112) min<br>4.0 (3.6-4.2); 86 (75-104) min<br>2.9 (2.5-3.7); 122 (102-190) min	<0.05; <0.05	Treatment with 70 and 200 mg of intravenous erythromycin is equally effective in accelerating gastric emptying in critically ill patients. Doses as low as 70 mg (approx. 1 mg/kg) accelerate gastric emptying in critically ill patients, improving the success of enteral feeding. This effect is observed only in patients with delayed gastric emptying.

Spapen et al 1995 (Belgium) [34]	Adult medical/surgical intensive care unit patients (21)	1) Cisapride 10 mg q6h NG;<br>2) No treatment.	Gastric residue over one-week; gastric emptying time $T_{1/2}$;<br>17.7±8.9 mL; 18±7 min<br>94.5±33.3 mL; 78±40 min	<0.001; <0.005	Gastric emptying in critically ill, sedated, and mechanically ventilated patients can be significantly improved by adding cisapride to a routine enteral feeding protocol.
Heyland et al 1996 (Canada) [36]	Mixed intensive care unit patients (72)	1) Cisapride 20 mg;<br>2) An identical placebo.	Differences (Day 2 - Day 1) in ΔC_{max}; Δt_{max}; ΔAUC_{180}<br>49.1±10.7; -40.8±12.0; 5534±1349<br>12.3±7.0; -4.2±10.4; 2832±769	0.005; 0.02; 0.09	Cisapride enhances gastric emptying in critically ill patients
Chapman et al, 2016 (Australia) [27]	Mixed intensive care unit patients (33)	**1) GSK962040 (50 mg) NG;**<br>2) GSK962040 (75 mg) NG;<br>3) Placebo.	Baseline vs. post gastric emptying time BTt½; AUC_{240}<br>0.65 (0.39,0.1.08); 2.50 (1.68,3.72)<br>1.85 (0.82,4.15); 0.72 (0.39,1.36)<br>1.21 (0.68,2.15); 1.33(0.85,2.06)	No report; no report	A single enteral dose of camicinal (50 mg), but not 75 mg, accelerates gastric emptying and increases glucose absorption in feed-intolerant critically ill patients.
Mokhtari et al 2009 (Islamic Republic of lran) [37]	Critically ill adult respiratory distress syndrome (ARDS) patients (32)	1) Ginger NG,<br>2) Placebo.	Feeding tolerated in the first 48 hrs; feeding tolerated during the entire study period<br>51%; 92%<br>57%; 93%	<0.005; 0.42	Supplementing the diet with ginger extract in ARDS patients reduces the delayed gastric emptying risk.

Guo JH, et al 2012 (China) [29]	Mixed intensive care unit patients (80)	1) **Traditional Chinese medicine group: Chenxia Sijunzi decoction;**<br>2) Western medicine group: mosapride dispersible tablets 5 mg and multienzyme tablets NG;<br>3) Control group: routine symptomatic treatment without any medicines to promote gastrointestinal function.	The time to bowel sound recovery; the time to passage of gas by anus recovery; the time to bowel movement recovery<br>41.02±7.52[a]; 49.90±6.95[a]; 58.22±6.71[a]<br>44.02±6.23[a]; 51.32±5.12[a]; 60.91±3.72[a]<br>54.62±5.51; 64.68±9.47; 78.20±7.11	[a]*P*<0.01	Chenxia Sijunzi decoction can promote severe patients' gastrointestinal function recovery. No significant differences in each testing index were found between the traditional Chinese medicine and Western medicine groups.
Kooshki et al 2018 (Iran) [30]	Mixed intensive care unit patients (60)	1) Fenugreek seed powder 3 g q12h NG;<br>2) Routine care.	GRV at the 5th day; diarrhea; constipation; respiratory aspiration at 5th/6th days<br>28.06±9.23; 1/30 (3.3%); 3/30 (10%); 1/30 (3.3%)<br>38.94±9.54; 6/30 (20%); 21/30 (70%); 10/30 (33.3%)	0.001; 0.04; 0.001; 0.005	Beneficial effects of fenugreek seeds on food intolerance in critically ill patients.
Tahershamsi et al 2018 (Iran) [40]	Mixed intensive care unit patients (50)	1) Gastrolit (Zataria multiflora) (20 drops) q8h× 4 days;<br>2) Placebo = water.	GRV on the second, third, and fourth days<br>The data could not be extracted	All P<0.0001	Gastrolit can decrease GRV in mechanically ventilated patients

Doi et al 2019 (Japan) [28]	Mixed intensive care unit patients	**1) Rikkunshito 5 g** q8h ×5 days;<br>2) Rikkunshito 2.5 g q8h×5 days;<br>3) No rikkunshito (control).	GRV; the percentage of the target energy at the 5th day; the target energy was achieved at the 5th day<br>No report; 62%; 63%<br>No report; 40%; 38%<br>No report; 59%; 56%	NS; NS; NS	Standard- or high-dose rikkunshito did not improve achievement of the enteral calorie target in critically ill adults.

FI, feeding intolerance; NS, not significant; [a]$P<0.01$ compared with the control group.

Effect on hospital or ICU length of stay

The effect of prokinetics on hospital length of stay was examined by five studies [26, 28-30, 32]. These five studies, which enrolled a total of 250 patients, demonstrated a significant difference in the hospital length of stay between the prokinetic agent-treated group and the control group (MD -3.21, 95% CI -5.35, -1.06; P = 0.003; I^2 = 28%) (**Fig 2**). Three studies evaluated the effect of prokinetics on ICU length of stay in the critical care setting [26, 28, 30]. These three studies, enrolling a total of 186 patients, showed that the prokinetic agents appear to have a positive effect on shortening ICU length of stay (MD -2.03, 95% CI -3.96, -0.10; P = 0.04; I^2 = 0%) **(Fig 3)**. Additionally, the separate effects of different prokinetics on the ICU length of stay and hospital length of stay are presented in **S3 Table**.

Fig 2. Forest plot for hospital length of stay outcomes. IV: inverse variance; CI: confidence interval**.**

Fig 3. Forest plot for ICU length of stay outcomes. IV: inverse variance; CI: confidence interval.

Effect on reported adverse events

Seven studies reported events that met the definition of adverse events in 757 critically ill patients [26-28, 30-32, 35]. The meta-analysis showed no significant difference in the risk of reported adverse events between the prokinetic agent group and the control group (RR 1.13, 95% CI 0.92, 1.38; P = 0.25; I^2 = 0%) **(S2 Fig)**.

Effect on all-cause mortality

The effect of prokinetic agents on all-cause mortality has been examined by six studies in 691 critically ill patients [26, 28, 31, 32, 34, 35]. There was no significant difference in all-cause mortality between the prokinetic agent group and the control group (RR 0.96, 95% CI 0.81, 1.14; P = 0.64; I^2 = 0%) **(S3 Fig)**.

Subgroup analysis

Although no significant heterogeneity was found, we performed subgroup analyses to determine whether important subgroup differences existed. In the subgroup analysis stratified by type of prokinetic agents, no significant subgroup differences were detected in the clinical outcomes of hospital length of stay, ICU length of stay, reported adverse events and all-cause mortality (**S4, 5, 6 and 7 Figs**). Furthermore, no study compared the combination of prokinetics to placebo or no treatment. Only one study about the preventive usage of prokinetics for risk patients demonstrated the outcome of reported adverse events [27]. The others were about the preventive usage of prokinetics for all patients. The subgroup analysis result of the preventive usage of prokinetics for all patients did not show important changes in the pooled effects of the reported adverse events.

Sensitivity analysis

The sensitivity analysis, which was performed by excluding the trials with a high risk of bias [28, 31], did not show important changes in the pooled effects of hospital length of stay, ICU length of stay, reported adverse events, or all-cause mortality. (Supplemental materials: **S8, 9, 10 and 11 Figs**).

Certainty of evidence

The certainty of evidence was moderate for the clinical outcome of all-cause mortality. However, the certainty of evidence was low for the clinical outcomes of ICU length of stay, hospital length of stay and reported adverse events. The details of the risk of bias and quality assessment are outlined in **Table 3**.

Table 3. GRADE evidence profile of the efficacy and safety of prokinetics in critically ill adult patients receiving gastric feeding tubes

Certainty assessment							No. of patients		Effect		Certainty	Importance
**No. of studies**	**Study design**	**Risk of bias**	**Inconsistency**	**Indirectness**	**Imprecision**	**Other considerations**	**Prokinetics**	**Placebo**	**Relative (95% CI)**	**Absolute (95% CI)**		
**Effect on ICU length of stay**												
3	randomized trials	serious [1]	not serious [2]	not serious [3]	serious [4]	none [5]	96	90	-	MD **2.03 days lower** (3.96 **days** lower to 0.1 **days** lower)	⊕⊕◯◯ LOW	IMPORTANT
**Effect on hospital length of stay**												
5	randomized trials	serious [6]	not serious [7]	not serious [3]	serious [8]	none [5]	141	109	-	MD **3.21 days lower** (5.35 **days** lower to 1.06 **days** lower)	⊕⊕◯◯ LOW	IMPORTANT
**Effect on reported adverse events**												
7	randomized trials	serious [9]	not serious [10]	not serious [3]	serious [11]	none	105/320 (32.8%)	120/437 (27.5%)	**RR 1.13** (0.92 to 1.38)	**40 more RAE per 1,000 patients** (from 20 fewer RAE to 100 more RAE)	⊕⊕◯◯ LOW	IMPORTANT
**Effect on all-cause mortality**												
6	randomized trials	serious [12]	not serious [13]	not serious [3]	not serious [14]	none	114/286 (39.9%)	174/405 (43.0%)	**RR 0.96** (0.81 to 1.14)	**30 fewer deaths per 1,000 patients** (from 100 fewer deaths to 40 more deaths)	⊕⊕⊕◯ MODERATE	CRITICAL

CI: confidence interval; RR: risk ratio; MD: mean difference; RAE: reported adverse events.
1. We downgraded the quality of evidence for risk of bias by one level. Two of three included studies had a high or unclear risk of bias.
2. We did not downgrade for inconsistency, I^2=0% and Chi^2 = 1.45, P=0.48.
3. Although the studies included any critically ill patient, we did not downgrade for indirectness.
4. We downgraded the quality of evidence for imprecision by one level because the total population size is less than 400. The 95% confidence interval contained a small benefit

that did not meet the clinical decision threshold (min. one day).

5. We did not downgrade for publication bias, although we could not assess this category reliably due to the small number of eligible studies. Not all included studies showed benefits of the studied intervention.

6. We downgraded the quality of evidence for risk of bias by one level. Most studies had an unclear risk of bias. In addition, one study lacked allocation concealment and blinding.

7. We did not downgrade for inconsistency, I^2=28% and Chi^2 = 5.52, P=0.24.

8. We downgraded the quality of evidence by one level for imprecision because the population size is less than 400.

9. We downgraded the quality of evidence for risk of bias by one level. Most studies had an unclear risk of bias. In addition, two studies lacked allocation concealment and/or blinding.

10. We did not downgrade for inconsistency, I^2=0% and Chi^2 = 1.64, P=0.95.

11. We downgraded the quality of evidence for imprecision by one level because the 95% confidence interval around the pooled effect included both no effect and appreciable harm (a relative risk increase greater than 25%).

12. We downgraded the quality of evidence for risk of bias by one level. Most studies had an unclear risk of bias. In addition, two studies lacked allocation concealment, and two studies lacked blinding.

13. We did not downgrade for inconsistency, I^2=0% and Chi^2 = 4.52, P=0.48.

14. We did not downgrade for imprecision because the 95% confidence interval around the pooled effect did not include both no effect and an appreciable benefit (a relative risk reduction greater than 25%) or appreciable harm (a relative risk increase greater than 25%).

Discussion

In this systematic review, we conducted a comprehensive literature search and used objective criteria for study inclusion. Fifteen studies were included in the final analysis. Because of small sample sizes and a relatively small amount of research, the accuracy of the pooled effect is lacking in quantitative analysis. Most studies (10 of 13, 76.92%) found that prokinetic agents showed beneficial effects on feeding tolerance in critically ill adults. The negative studies (3 of 13, 23.08%) were hampered by special populations of neuro-critical patients and critical traumatic brain injury patients taking metoclopramide and by the use of the specific drug rikkunshito. Furthermore, the use of prokinetic agents in critically ill patients receiving gastric feeding may reduce the ICU or hospital length of stay, but the certainty of evidence was low due to risk of bias and imprecision. Prokinetics did not significantly reduce the risks of reported adverse events or all-cause mortality.

In this study, we examined the effect of prokinetic agents on gastrointestinal symptoms, feeding tolerance and clinical outcomes. Compared to the control group, prokinetics did not reduce the risk of mortality; these results were the same as the results of the meta-analysis by Lewis, K. et al. [14], but our methods are different. Lewis, K. et al. [14] defined feeding intolerance as either GRV ≥150 mL, vomiting, or abdominal distention resulting in feeding interruption. This definition may be considered obsolete [15]. We defined gastric feeding intolerance as either GRV ≥500 mL or concomitant symptoms of nausea, vomiting, abdominal distention, regurgitation or other symptoms resulting in feeding interruption in critically ill adult patients receiving gastric feeding tubes. We excluded studies that discontinued or interrupted gastric feeding prematurely following the disappearance of gastric feeding intolerance. Under this latest definition, our meta-analysis found some new studies [26-30, 33, 34], and we identified 5 studies regarding the administration of prokinetics including herbal medicines/natural medicines in critically ill adult patients receiving gastric feeding tubes [28-30, 37, 40].

Additionally, we found that prokinetic agents might reduce the ICU or hospital length of stay for

critically ill patients receiving gastric feeding. However, the number of studies and the sample size were very small, and the certainty of evidence was low. Furthermore, no significant difference was found between prokinetic agent groups and placebo/no treatment in the risks of reported adverse events and all-cause mortality. Therefore, we cannot draw a convincing conclusion that the use of prokinetics can improve clinical outcomes in critically ill adults. We recommend that more research should be conducted in this field.

This study has several limitations. First, RCTs from 21 published original studies or trials registered in the International Clinical Trials Registry Platform (WHO) or clinicaltrials.gov were identified. However, 6 trials were completed, but the results were not available, which might lead to the omission of trials meeting the inclusion criteria and might have publication bias. Second, some included trials did not test the baseline status of feeding intolerance for all participants. The subgroup results might have been different if all individuals were tested. Third, we were unable to comprehensively evaluate the risk of bias in 12 studies with a lack of information. Fourth, in each outcome, the total sample size was relatively small, which likely had inadequate power to detect a difference in treatment effect. We recommend that more original studies about this topic be conducted.

Conclusion

As a class of drugs, prokinetic agents may improve gastric feeding tolerance in critically ill adults. However, the certainty of the evidence suggesting that prokinetic agents are effective for reducing the ICU or hospital length of stay is low. There was also no significant reduction in the risk of reported adverse events and all-cause mortality. Additional RCTs are needed to determine the effect of prokinetics on clinical outcomes in critically ill patients in the future.

Acknowledgments

We are grateful to all the staff in this study for their teamwork and persistent efforts, and we are also thankful to the Group of People with Highest Risk of Drug Exposure of the International Network for

the Rational Use of Drugs, China, and the Evidence-Based Pharmacy Committee of the Chinese Pharmaceutical Association for providing methodologies.

Ethics approval and consent to participate

Not applicable.

Competing interests

We declare that we have no conflicts of interest.

Funding

The funders had no role in the study design, data collection and analysis, the decision to publish, or preparation of the manuscript.

Author Contributions

LLZ conceived the study and designed the study. RP and HLL had full access to all of the data in the study, and take responsibility for the integrity of the data and the accuracy of the data analysis. RP and HLL developed and tested the data collection form. All authors acquired the data. HLL and RP conducted the analysis, interpreted the data, and drafted the manuscript. All authors critically revised the manuscript. LLZ is the guarantor.

Consent for publication

Written informed consent for publication was obtained from all participants.

Availability of data and material

All data generated or analyzed during this study are included in this published article.

References

1. Lew CCH, Yandell R, Fraser RJL, Chua AP, Chong MFF, Miller M. Association Between Malnutrition and Clinical Outcomes in the Intensive Care Unit: A Systematic Review [Formula: see text]. *JPEN J Parenter Enteral Nutr.* 2017;41(5):744-758.

2. McClave SA, Taylor BE, Martindale RG, Warren MM, Johnson DR, Braunschweig C, McCarthy MS, Davanos E, Rice TW, Cresci GA, Gervasio JM, Sacks GS, Roberts PR, Compher C, Society of Critical Care M, American Society for P, Enteral N. Guidelines for the Provision and Assessment of Nutrition Support Therapy in the Adult Critically Ill Patient: Society of Critical Care Medicine (SCCM) and American Society for Parenteral and Enteral Nutrition (A.S.P.E.N.). *JPEN J Parenter Enteral Nutr.* 2016;40(2):159-211.

3. Singer P, Blaser AR, Berger MM, Alhazzani W, Calder PC, Casaer MP, Hiesmayr M, Mayer K, Montejo JC, Pichard C, Preiser JC, van Zanten ARH, Oczkowski S, Szczeklik W, Bischoff SC. ESPEN guideline on clinical nutrition in the intensive care unit. *Clin Nutr.* 2019;38(1):48-79.

4. Elke G, Felbinger TW, Heyland DK. Gastric residual volume in critically ill patients: a dead marker or still alive?. [Review].1.

5. Blaser AR, Starkopf J, Kirsimagi U, Deane AM. Definition, prevalence, and outcome of feeding intolerance in intensive care: a systematic review and meta-analysis. *Acta Anaesthesiol Scand.* 2014;58(8):914-922.

6. Reintam Blaser A, Malbrain ML, Starkopf J, Fruhwald S, Jakob SM, De Waele J, Braun JP, Poeze M, Spies C. Gastrointestinal function in intensive care patients: terminology, definitions and management. Recommendations of the ESICM Working Group on Abdominal Problems. *Intensive Care Med.* 2012;38(3):384-394.

7. Hu B, Sun R, Wu A, Ni Y, Liu J, Guo F, Ying L, Ge G, Ding A, Shi Y, Liu C, Xu L, Jiang R, Lu J, Lin R, Zhu Y, Wu W, Xie B. Prognostic Value of Prolonged Feeding Intolerance in Predicting All-Cause Mortality in Critically Ill Patients: A Multicenter, Prospective, Observational Study. *JPEN J Parenter Enteral Nutr.* 2019.

8. Gungabissoon U, Hacquoil K, Bains C, Irizarry M, Dukes G, Williamson R, Deane AM, Heyland DK. Prevalence, risk factors, clinical consequences, and treatment of enteral feed intolerance during critical illness. *JPEN J Parenter Enteral Nutr.* 2015;39(4):441-448.

9. Heyland DK, Dhaliwal R, Drover JW, Gramlich L, Dodek P, Canadian Critical Care Clinical Practice Guidelines C. Canadian clinical practice guidelines for nutrition support in mechanically ventilated, critically ill adult patients. *JPEN J Parenter Enteral Nutr.* 2003;27(5):355-373.

10. Consensus of early enteral nutrition clinical practice in critically ill patients. *Chinese Critical Care Medicine.* 2018;30(8):715-721.

11. Taylor BE, McClave SA, Martindale RG, Warren MM, Johnson DR, Braunschweig C, McCarthy MS, Davanos E, Rice TW, Cresci GA, Gervasio JM, Sacks GS, Roberts PR, Compher C, Society of Critical Care M, American Society of P, Enteral N. Guidelines for the Provision and Assessment of Nutrition Support Therapy in the Adult Critically Ill Patient: Society of Critical Care Medicine (SCCM) and

American Society for Parenteral and Enteral Nutrition (A.S.P.E.N.). *Crit Care Med.* 2016;44(2):390-438.

12. Reintam Blaser A, Starkopf J, Alhazzani W, Berger MM, Casaer MP, Deane AM, Fruhwald S, Hiesmayr M, Ichai C, Jakob SM, Loudet CI, Malbrain ML, Montejo Gonzalez JC, Paugam-Burtz C, Poeze M, Preiser JC, Singer P, van Zanten AR, De Waele J, Wendon J, Wernerman J, Whitehouse T, Wilmer A, Oudemans-van Straaten HM, Function EWGoG. Early enteral nutrition in critically ill patients: ESICM clinical practice guidelines. *Intensive Care Med.* 2017;43(3):380-398.

13. Peng R. The efficacy and safety of administration of prokinetics improve clinical outcomes in critically ill patients is still quite unclear. *Clinical Nutrition.* 2020;39:307-309.

14. Lewis K, Alqahtani Z, McIntyre L, Almenawer S, Alshamsi F, Rhodes A, Evans L, Angus DC, Alhazzani W. The efficacy and safety of prokinetic agents in critically ill patients receiving enteral nutrition: A systematic review and meta-analysis of randomized trials. *Critical Care.* 2016;20 (1) (no pagination)(259).

15. Dive A. Benefit of prokinetics during enteral nutrition: still searching for a piece of evidence. *Crit Care.* 2016;20(1):341.

16. Higgins JPT. Green S, eds. Cochrane Handbook for Systematic Reviews of Interventions, version 5.1.0 (updated March 2011). Cochrane Collaboration website. http://training.cochrane.org/handbook. 2011. Accessed November 22, 2017.

17. Liberati A, Altman DG, Tetzlaff J, Mulrow C, Gotzsche PC, Ioannidis JP, Clarke M, Devereaux PJ, Kleijnen J, Moher D. The PRISMA statement for reporting systematic reviews and meta-analyses of studies that evaluate health care interventions: explanation and elaboration. *J Clin Epidemiol.* 2009;62(10):e1-34.

18. Gupta SK. Intention-to-treat concept: A review. *Perspect Clin Res.* 2011;2(3):109-112.

19. Schulz KF, Altman DG, Moher D, Group C. CONSORT 2010 statement: updated guidelines for reporting parallel group randomised trials. *PLoS Med.* 2010;7(3):e1000251.

20. Luo D, Wan X, Liu J, Tong T. Optimally estimating the sample mean from the sample size, median, mid-range, and/or mid-quartile range. *Stat Methods Med Res.* 2018;27(6):1785-1805.

21. Wan X, Wang W, Liu J, Tong T. Estimating the sample mean and standard deviation from the sample size, median, range and/or interquartile range. *BMC Med Res Methodol.* 2014;14:135.

22. Deeks JJ. Issues in the selection of a summary statistic for meta-analysis of clinical trials with binary outcomes. *Stat Med.* 2002;21(11):1575-1600.

23. Borenstein M, Hedges LV, Higgins JP, Rothstein HR. A basic introduction to fixed-effect and random-effects models for meta-analysis. *Res Synth Methods.* 2010;1(2):97-111.

24. Furukawa TA, Watanabe N, Omori IM, Montori VM, Guyatt GH. Association between unreported outcomes and effect size estimates in Cochrane meta-analyses. *JAMA.* 2007;297(5):468-470.

25. Guyatt GH, Oxman AD, Vist GE, Kunz R, Falck-Ytter Y, Alonso-Coello P, Schunemann HJ, Group GW. GRADE: an emerging consensus on rating quality of evidence and strength of recommendations. *BMJ.* 2008;336(7650):924-926.

26. Acosta-Escribano J, Almanza Lopez S, Plumed Martin L, Garcia Martinez MA, Tajadura Manjarin N. The metoclopramide effect on enteral nutrition tolerance and mechanical ventilation associated pneumonia in neuro critically ill patients. *Nutricion hospitalaria.* 2014;29(6):1345-1351.

27. Chapman MJ, Deane AM, O'Connor SL, Nguyen NQ, Fraser RJL, Richards DB, Hacquoil KE, Vasist Johnson LS, Barton ME, Dukes GE. The effect of camicinal (GSK962040), a motilin agonist, on gastric emptying and glucose absorption in feed-intolerant critically ill patients: a randomized, blinded, placebo-controlled, clinical trial https://www.ncbi.nlm.nih.gov/pmc/articles/PMC4967996/. *Critical care.* 2016;20(1).

28. Doi M, Miyamoto K, Shimokawa T, Ariyasu H, Kaneko M, Yonemitsu T, Nakashima T, Matsumoto H, Shibata M, Shibata N, Soda M, Kitaichi K, Kato S. The effect of standard and high dose of rikkunshito on achievement of enteral nutrition target in critically ill patients: a pilot randomized controlled trial. *Acute medicine and surgery.* 2019.

29. Guo JH, Chen G, Yang SQ, Wei MH, Chen X. Clinical observation of the role of Chenxia Sijunzi decoction in promoting the recovery of gastrointestinal function in critically ill patients. *Zhongguo wei zhong bing ji jiu yi xue [Chinese critical care medicine].* 2012;24(11):674-676.

30. Kooshki A, Khazaei Z, Zarghi A, Rad M, Mohammadi HG, Tabaraie Y. Effects of fenugreek seed powder on enteral nutrition tolerance and clinical outcomes in critically ill patients: a randomized clinical trial. *Biomedical research and therapy.* 2018;5(7):2528-2537.

31. Nassaji M, Ghorbani R, Frozeshfard M, Mesbahian F. Effect of metoclopramide on nosocomial pneumonia in patients with nasogastric feeding in the intensive care unit. *La revue de sante de la Mediterranee orientale / al-Majallah al-sihhiyah li-sharq al-mutawassit [Eastern Mediterranean health journal].* 2010;16(4):371-374.

32. Nursal TZ, Erdogan B, Noyan T, Cekinmez M, Atalay B, Bilgin N. The effect of metoclopramide on gastric emptying in traumatic brain injury. *J Clin Neurosci.* 2007;14(4):344-348.

33. Ritz MA, Chapman MJ, Fraser RJ, Finnis ME, Butler RN, Cmielewski P, Davidson GP, Rea D. Erythromycin dose of 70 mg accelerates gastric emptying as effectively as 200 mg in the critically ill. *Intensive care medicine.* 2005;31(7):949-954.

34. Spapen HD, Duinslaeger L, Diltoer M, Gillet R, Bossuyt A, Huyghens LP. Gastric emptying in critically ill patients is accelerated by adding cisapride to a standard enteral feeding protocol: results of a prospective, randomized, controlled trial. *Critical care medicine.* 1995;23(3):481-485.

35. Yavagal DR, Karnad DR, Oak JL. Metoclopramide for preventing pneumonia in critically ill patients receiving enteral tube feeding: a randomized controlled trial. *Critical care medicine.* 2000;28(5):1408-1411.

36. Heyland DK, Tougas G, Cook DJ, Guyatt GH. Cisapride improves gastric emptying in mechanically ventilated, critically ill patients. A randomized, double-blind trial. *American journal of respiratory and critical care medicine.* 1996;154(6 Pt 1):1678-1683.

37. Mokhtari M, Shariatpanahi Z. Ginger extract dietary supplementation effects on delayed gastric emptying and ventilator-associated pneumonia in adult respiratory distress syndrome patients. *Critical Care.* 2009;1):S56.

38. Rajan V, Maiwall R, Choudhury AK, Jamwal KD, Benjamin J, Kumar G, Joshi YK, Sarin SK. Early addition of prokinetics reverses gut paralysis and improves survival in critically ill cirrhotics-An open label placebo controlled RCT (Feed Intolerance and Treatment-FIT protocol). NCT02528760. *Hepatology*. 2017;66.

39. Sustic A, Zelic M, Protic A, Zupan Z, Simic O, Desa K. Metoclopramide improves gastric but not gallbladder emptying in cardiac surgery patients with early intragastric enteral feeding: randomized controlled trial. *Croatian medical journal.* 2005;46(2):239-244.

40. Tahershamsi F, Rezaei K, Khosravi S, Kheyri Z, Memarzadeh MR, Rafie F. Effect of oral drop gastrolit (Zataria multiflora) on gastric residual volume in mechanically ventilated patients hospitalized in the intensive care units. *Journal of medicinal plants.* 2018;17(68):66-73.

41. Chapman MJ, Fraser RJ, Kluger MT, Buist MD, De Nichilo DJ. Erythromycin improves gastric emptying in critically ill patients intolerant of nasogastric feeding. *Critical care medicine.* 2000;28(7):2334-2337.

42. Deane AM, Lamontagne F, Dukes GE, Neil D, Vasist L, Barton ME, Hacquoil K, Ou X, Richards D, Stelfox HT, Mehta S, Day AG, Chapman MJ, Heyland DK. Nutrition Adequacy Therapeutic Enhancement in the Critically Ill: a Randomized Double-Blind, Placebo-Controlled Trial of the Motilin Receptor Agonist Camicinal (GSK962040): the NUTRIATE Study. *Journal of parenteral and enteral nutrition.* 2018;42(5):949-959.

43. Pinilla JC, Samphire J, Arnold C, Liu L, Thiessen B. Comparison of gastrointestinal tolerance to two enteral feeding protocols in critically ill patients: a prospective, randomized controlled trial. *Journal of Parenteral and Enteral Nutrition* 2001;25(2):81-6.

44. Reignier J, Bensaid S, Perrin-Gachadoat D, Burdin M, Boiteau R, Tenaillon A. Erythromycin and early enteral nutrition in mechanically ventilated patients. *Critical care medicine.* 2002;30(6):1237-1241.

45. Taylor SJ, Allan K, McWilliam H, Manara A, Brown J, Greenwood R, Toher D. A randomised controlled feasibility and proof-of-concept trial in delayed gastric emptying when metoclopramide fails: we should revisit nasointestinal feeding versus dual prokinetic treatment: achieving goal nutrition in critical illness and delayed gastric emptying: trial of nasointestinal feeding versus nasogastric feeding plus prokinetics. *Clinical nutrition ESPEN.* 2016;14:1-8.

46. Boivin MA, Levy H. Gastric feeding with erythromycin is equivalent to transpyloric feeding in the critically ill. *Critical care medicine.* 2001;29(10):1916-1919.

47. Euctr ES. A Phase 2, Multicenter, Randomized, Double-Blind, Comparator-Controlled Study of the Efficacy, Safety, and Pharmacokinetics of Intravenous Ulimorelin (LP101) in Patients with Enteral Feeding Intolerance. *http://www.who.int/trialsearch/Trial2.aspx?TrialID=EUCTR2016*. 2016.

48. Hayakawa M, Ono Y, Wada T, Yanagida Y, Sawamura A, Takeda H, Gando S. Effects of rikkunshito (traditional Japanese medicine) on enteral feeding and the plasma ghrelin level in critically ill patients: a pilot study. *Journal of intensive care.* 2014;2(1).

49. Heyland DK, van Zanten ARH, Grau-Carmona T, Evans D, Beishuizen A, Schouten J, Hoiting O, Bordeje ML, Krell K, Klein DJ, Gonzalez J, Perez A, Brown R, James J, Harris MS, Heyland DK, Evans DC, Jolley S, Raines R, Krell K, Falls I, Grau-Carmona T, Servia-Goixart L, Perez-Quesada S,

Herrero-Meseguer JI, Calvo-Herranz E, Lorencio C, Peredes A, Yebenes-Reyes JC, Garcia-Martinez MA, Cervera M, Bordeje ML, Fernadez-Ortega JF, Fernandez-Gonzalez I, van Zanten A, Beishuizen A, Schouten J, Hoiting O, Wilhelmina C, Stelfox T, Posadas J. A multicenter, randomized, double-blind study of ulimorelin and metoclopramide in the treatment of critically ill patients with enteral feeding intolerance: PROMOTE trial. *Intensive care medicine.* 2019;45(5):647-656.

50. Irct201112014578N. The effect of acupuncture and traditional pharmacologic therapy on Delayed Gastric Emptying. *http://www.who.int/trialsearch/Trial2.aspx?TrialID=IRCT201112014578N4*. 2012.

51. Irct201408104365N. Combination of Neostigmine and Metoclopramide to reduce gastric residual volume in mechanically ventilated ICU patients. *http://www.who.int/trialsearch/Trial2.aspx?TrialID=IRCT201408104365N16*. 2015.

52. Irct201412044365N. Comparison of the effect of Neostigmine and Metoclopramide on gastric residual volume in mechanically ventilated ICU patients. *http://www.who.int/trialsearch/Trial2.aspx?TrialID=IRCT201412044365N18*. 2014.

53. MacLaren R, Patrick WD, Hall RI, Rocker GM, Whelan GJ, Lima JJ. Comparison of cisapride and metoclopramide for facilitating gastric emptying and improving tolerance to intragastric enteral nutrition in critically III, mechanically ventilated adults. *Clinical therapeutics.* 2001;23(11):1855-1866.

54. Makkar JK, Gauli B, Jain K, Jain D, Batra YK. Comparison of erythromycin versus metoclopramide for gastric feeding intolerance in patients with traumatic brain injury: a randomized double-blind study. *Saudi journal of anaesthesia.* 2016;10(3):308-313.

55. Malekolkottab M, Khalili H, Mohammadi M, Ramezani M, Nourian A. Metoclopramide as intermittent and continuous infusions in critically ill patients: a pilot randomized clinical trial. *Journal of comparative effectiveness research.* 2017;6(2):127-136.

56. Nct. A Randomized, Double-Blind Study to Evaluate the Safety, Tolerability, and Pharmacodynamics of a Single Dose of Intravenous TD-8954 Compared With Metoclopramide in Critically Ill Patients With Enteral Feeding Intolerance. *https://clinicaltrials.gov/show/NCT01953081*. 2013.

57. Nct. Itopride in Feeding Intolerance of Critically-ill Patients Receiving Enteral Nutrition. *https://clinicaltrials.gov/show/NCT03698292*. 2018.

58. Nguyen N, Chapman M, Fraser R, Sharley V, Kong S, Bryant L, et al. Erythromycin or metoclopramide for feed intolerance in the critically ill. *Critical care.* 2006;10(Suppl 1).

59. Taylor S, Manara A, Brown J. Treating delayed gastric emptying in critical illness: metoclopramide, erythromycin and bedside (Cortrak[TM]) nasointestinal tube placement. *Journal of the intensive care society.* 2011;12(1):75-.

60. Yanagida Y, Hayakawa M, Yamamoto H, Wada T, Sugano M, Sawamura A, Gando S. Effects of rikkunshito (traditional Japanese medicine Kampo) on enteral feeding and plasma ghrelin concentration in critically ill patients: a double-blind, randomized, controlled trial. *Intensive care medicine.* 2013;39:S242-S243.

61. Berne JD, Norwood SH, McAuley CE, Vallina VL, Villareal D, Weston J, McClarty J. Erythromycin reduces delayed gastric emptying in critically ill trauma patients: a randomized, controlled trial. *Journal of trauma.* 2002;53(3):422-425.

62. Meissner W, Dohrn B, Reinhart K. Enteral naloxone reduces gastric tube reflux and frequency of pneumonia in critical care patients during opioid analgesia. *Critical care medicine.* 2003;31(3):776-780.

63. Shariatpanahi ZV, Taleban FA, Mokhtari M, Shahbazi S. Ginger extract reduces delayed gastric emptying and nosocomial pneumonia in adult respiratory distress syndrome patients hospitalized in an intensive care unit. *Journal of critical care.* 2010;25(4):647-650.

64. Tamion F, Hamelin K, Duflo A, Girault C, Richard JC, Bonmarchand G. Gastric emptying in mechanically ventilated critically ill patients: effect of neuromuscular blocking agent. *Intensive care medicine.* 2003;29(10):1717-1722.

65. Chen JH, Hsieh CB, Chao PC, Liu HD, Chen CJ, Liu YC, Yu JC. Effect of water-soluble contrast in colorectal surgery: a prospective randomized trial. *World journal of gastroenterology.* 2005;11(18):2802-2805.

66. Goldhill DR, Toner CC, Tarling MM, Baxter K, Withington PS, Whelpton R. Double-blind, randomized study of the effect of cisapride on gastric emptying in critically ill patients. *Critical care medicine.* 1997;25(3):447-451.

67. Warusevitane A, Karunatilake D, Sim J, Lally F, Roffe C. Safety and effect of metoclopramide to prevent pneumonia in patients with stroke fed via nasogastric tubes trial. *Stroke.* 2015;46(2):454-460.

68. Tctr. Efficacy and safety of oral Erythromycin estolate in combination with Metoclopramide versus Metoclopramide mOnotherapy in mechanically ventilated patients who developed enteral feeding intolerance: a randomized double-blind controlled study. *http://www.who.int/trialsearch/Trial2.aspx?TrialID=TCTR20171004004*. 2017.

69. NCT02528760. To Determine the Role of Prokinetics in Feed Intolerance in Critically Ill Cirrhosis. *https://clinicaltrials.gov/show/NCT02528760*. 2015.

70. NCT02379624. Pectin Start Early Enteral Nutritional Support in Critically Ill Patients. *https://clinicaltrials.gov/show/NCT02379624*. 2014.

71. IRCT201610297494N24. The prophylactic effect of cuminum cyminum extract on gastric residual volume in traumatic patients under ventilator hospitalized in intensive care unit. *http://www.who.int/trialsearch/Trial2.aspx?TrialID=IRCT201610297494N24*. 2017.

72. IRCT201009094722N2. The effect of Ginger extract on gastric residual volume in patients with mechanical ventilation hospitalized in intensive care unit. *http://www.who.int/trialsearch/Trial2.aspx?TrialID=IRCT201009094722N2*. 2011.

73. 2015/08/006111 C. Clinical trial to compare the efficacy of two oral prokinetics metoclopramide and erythromycin in non acceptance of feed in head injury patient. *http://www.who.int/trialsearch/Trial2.aspx?TrialID=CTRI*. 2015;08(006111).

Supporting information

S1 Table. Search strategy

S2 Table. Excluded studies

S3 Table. Separate effects of different prokinetic agents on hospital or ICU length of stay

S4 Table. PRISMA checklist

S1 Fig. Risk of bias

S2 Fig. Reported adverse event outcomes

S3 Fig. All-cause mortality outcomes

S4 Fig. Subgroup analysis by the type of prokinetic agents for hospital length of stay outcomes

S5 Fig. Subgroup analysis by the type of prokinetic agents for ICU length of stay outcomes

S6 Fig. Subgroup analysis by the type of prokinetic agents for reported adverse event outcomes

S7 Fig. Subgroup analysis by the type of prokinetic agents for all-cause mortality outcomes

S8 Fig. Sensitivity analysis of hospital length of stay outcomes

S9 Fig. Sensitivity analysis of ICU length of stay outcomes

S10 Fig. Sensitivity analysis of reported adverse event outcomes

S11 Fig. Sensitivity analysis of all-cause mortality outcomes

Fig 1

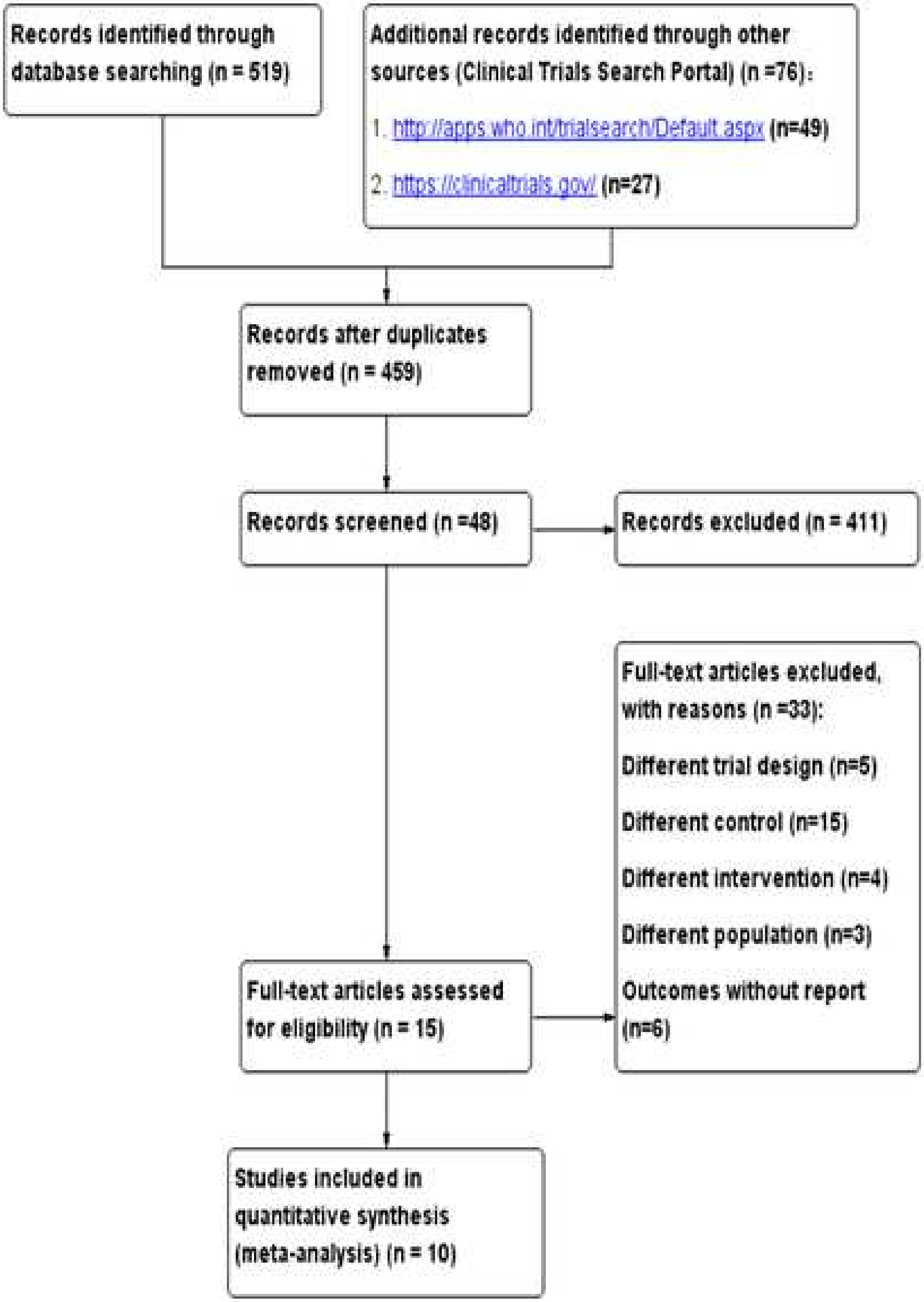
Records identified through database searching (n = 519)
Additional records identified through other sources (Clinical Trials Search Portal) (n =76):
1. http://apps.who.int/trialsearch/Default.aspx (n=49)
2. https://clinicaltrials.gov/ (n=27)
Records after duplicates removed (n = 459)
Records screened (n =48)
Records excluded (n = 411)
Full-text articles excluded, with reasons (n =33):
Different trial design (n=5)
Different control (n=15)
Different intervention (n=4)
Different population (n=3)
Outcomes without report (n=6)
Full-text articles assessed for eligibility (n = 15)
Studies included in quantitative synthesis (meta-analysis) (n = 10)

Figure 2

Click here to access/download;Figure;Fig 2.tif

Study or Subgroup	Prokinetics Mean [Days]	Prokinetics SD [Days]	Prokinetics Total	Control Mean [Days]	Control SD [Days]	Control Total	Weight	Mean Difference IV, Random, 95% CI [Days]
Acosta-Escribano J, et al (2014)	36	29	58	31	21	51	4.8%	5.00 [-4.43, 14.43]
Doi M, et al (2019)	35	8.94	8	57.86	41.11	9	0.6%	-22.86 [-50.42, 4.70]
Guo JH, et al (2012)	5.1	1.7	35	8.9	1.4	10	60.6%	-3.80 [-4.83, -2.77]
Kooshki A, et al (2018)	24.1	5.6	30	27.4	6.6	30	28.6%	-3.30 [-6.40, -0.20]
Nursal, T. Z., et al (2007)	15.6	11.1	10	16.8	8.5	9	5.4%	-1.20 [-10.04, 7.64]
**Total (95% CI)**			**141**			**109**	**100.0%**	**-3.21 [-5.35, -1.06]**

Heterogeneity: $Tau^2 = 1.70$; $Chi^2 = 5.52$, df = 4 (P = 0.24); $I^2 = 28\%$

Test for overall effect: Z = 2.93 (P = 0.003)

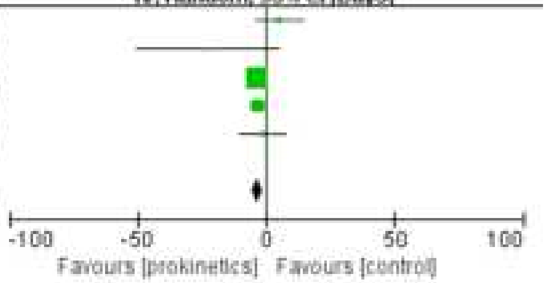

Figure 3

Click here to access/download;Figure;Fig 3.tif

Study or Subgroup	Prokinetics Mean [Days]	Prokinetics SD [Days]	Prokinetics Total	Control Mean [Days]	Control SD [Days]	Control Total	Weight	Mean Difference IV, Random, 95% CI [Days]
Acosta-Escribano J, et al (2014)	14	8	58	15	9	51	36.1%	-1.00 [-4.22, 2.22]
Doi M, et al (2019)	5.63	4.47	8	6.63	4.37	9	21.0%	-1.00 [-5.21, 3.21]
Kooshki A, et al (2018)	14.2	4.8	30	17.6	6.7	30	42.9%	-3.40 [-6.35, -0.45]
**Total (95% CI)**			**96**			**90**	**100.0%**	**-2.03 [-3.96, -0.10]**

Heterogeneity: Tau² = 0.00; Chi² = 1.45, df = 2 (P = 0.48); I² = 0%
Test for overall effect: Z = 2.06 (P = 0.04)

Mean Difference
IV, Random, 95% CI [Days]

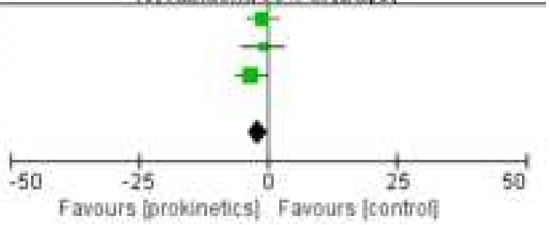

Click here to access/download
Supporting Information
Supplementary materials. 2020.06.13.docx

The efficacy and safety of prokinetics in critically ill adults receiving gastric feeding tubes: ~~a~~ A systematic review and meta-analysis

Rong Peng[1,2,3,4], Hailong Li [1,2,3], Lijun Yang [5], Linan Zeng [1,2,3], Qiusha Yi [1,2,3], Peipei Xu [1,2,3], Xiangcheng Pan [1,2,3], Lingli Zhang [1,2,3*]

[1] Department of Pharmacy, West China Second University Hospital, Sichuan University, Chengdu, Sichuan, China

[2] Evidence-Based Pharmacy Center, West China Second University Hospital, Sichuan University, Chengdu, Sichuan, China

[3] Key Laboratory of Birth Defects and Related Diseases of Women and Children (Sichuan University), Ministry of Education, Chengdu, Sichuan, China

[4] Department of Clinical Nutrition, Affiliated Hospital of Chengdu University, Chengdu, Sichuan, China

[5] Department of General Practice Medicine, Affiliated Hospital of Chengdu University, Chengdu, Sichuan, China

*Corresponding Author: Lingli Zhang, Department of Pharmacy, West China Second University Hospital, Sichuan University. Renming Road, Chengdu, Sichuan, 610041, China. Tel: 0862885502378

E-mail: zhanglingli@scu.edu.cn

Abstract

Background: Intolerance to gastric feeding tubes is common ~~in~~ among critically ill adults and may increase morbidity. Administration of prokinetics in the ICU is common. However, the efficacy and safety of prokinetics are unclear in critically ill adults with gastric feeding tubes. We conducted a systematic review to determine the efficacy and safety of prokinetics for improving gastric feeding tube tolerance in critically ill adults.

Methods: Randomized controlled trials (RCTs) were identified by systematically searching the Medline, Cochrane and Embase databases. Two independent reviewers extracted ~~the~~ relevant data and assessed the quality of the studies. We calculated pooled relative risks (RRs) for dichotomous outcomes and the mean differences (MDs) for continuous outcomes~~,~~ with the corresponding 95% confidence intervals (CIs). We assessed the risk of bias using the Cochrane risk-of-bias tool and used the Grading of Recommendations Assessment, Development, and Evaluation (GRADE) methodology to rate the quality of the evidence.

Results: Fifteen RCTs met the inclusion criteria. A total of 10 RCTs involving 846 participants were eligible for the quantitative analysis. ~~A majority of~~Most studies (10 of 13, 76.92%) found that prokinetics showed beneficial effects on feeding intolerance in critically ill adults. In critically ill adults receiving gastric feeding, prokinetic agents may reduce the ICU length of stay (MD -2.03, 95% CI -3.96, -0.10; P = 0.04; low certainty) and the hospital length of stay (MD -3.21, 95%CI -5.35, -1.06; P = 0.003; low certainty). However, prokinetics failed to improve the outcomes of reported adverse events~~,~~ and all-cause mortality.

Conclusion: As a class of drugs, prokinetics may improve tolerance to gastric feeding to some extent in critically ill adults. However, ~~there is low degree of~~the certainty ~~in~~of the evidence suggesting that prokinetics reduce the ICU or hospital length of stay is low. Prokinetics did not significantly decrease the risks of reported adverse events or all-cause mortality ~~in~~among critically ill adults.

Keywords: Prokinetics; Critical illness; Gastroparesis; Enteral nutrition; Systematic review.

Introduction

Critical illness is usually associated with catabolic stress and increases the incidence of infection and multiple organ dysfunction, bringing disproportionately high mortality. A systematic review found a strikingly high prevalence of malnutrition in intensive care unit (ICU) patients (ranging from 38% to 78%) [1]. Owing to the benefits of nutrition support in reducing disease severity and favorably impacting patient outcomes, early nutrition support therapy, primarily by the enteral route, is seen as a proactive therapeutic strategy [2]. In addition, if oral intake is not possible, tube feeding through gastric access has been recommended as the standard approach to initiate enteral nutrition in adult critically ill patients [3], as gastric feeding provides greater nutritional and non-nutritional benefits than parenteral nutrition and is more likely to improve the prognosis [4]. Furthermore, gastric feeding is more physiological than postpyloric feeding and does not require a higher level of technology [3].

However, enteral tube feeding intolerance occurs frequently in critically ill patients. Blaser et al. reported that the pooled proportion of feeding intolerance was 38.3% (95% confidence interval (CI) 30.7–46.2%) [5]. Feeding intolerance was first described by the Working Group on Abdominal Problems of the European Society of Intensive Care Medicine as failure to provide adequate enteral nutrition to critically ill patients for clinical reasons (vomiting, high gastric residual volume (GRV), diarrhea, gastrointestinal bleeding, presence of entero-cutaneous fistulas, etc.); if a feeding rate of at least 20 kcal/kg body weight (BW)/day cannot be reached via the enteral route in 72 h of feeding attempts or if enteral feeding must be stopped for any clinical reason, feeding intolerance should be considered present [6]. However, feeding intolerance is inconsistently defined in different studies, with the definitions falling into three main categories: (1) “large” GRV; (2) presence of gastrointestinal symptoms; or 3) inadequate delivery of enteral nutrition [5]. Feeding intolerance is associated with increasing mortality, and seven-day feeding intolerance is an independent predictor of 60-day mortality [7]. In addition, a meta-analysis by the European Society for Clinical Nutrition and Metabolism showed that gastric feeding intolerance was more prevalent than postpyloric feeding

intolerance (25.7% vs. 3.5%, p=0.0005) [3]. Given the risk associated with gastric feeding intolerance, it should be treated aggressively.

There are three methods for the treatment of gastric feeding intolerance. First, there is the most widely used method, the administration of prokinetics. Among recipients of gastric feeding, 13% had been prescribed prokinetics preemptively before they developed intolerance. Approximately one-third of patients who developed feeding intolerance were treated with a prokinetic agent during their stay in the ICU. Second, after the development of intolerance, 17% of patients received supplemental parenteral nutrition. Third, only 7.5% of patients with gastric feeding intolerance subsequently received enteral nutrition via a postpyloric feeding tube [8]. Although the use of prokinetics in the ICU is common, the recommendations vary from one authority to another. For example, the ESPEN guidelines on clinical nutrition in the ICU [3] suggest that intravenous erythromycin should be used as a first-line prokinetic therapy in critically ill patients with gastric feeding intolerance (grade of recommendation: B – strong consensus, 100% agreement). Alternatively, intravenous metoclopramide or a combination of metoclopramide and erythromycin can be used as a prokinetic therapy (Grade of recommendation: 0 – strong consensus, 100% agreement). However, the ASPEN/SCCM guidelines weakly recommend the use of gastrointestinal motility agents in the case of feeding intolerance (low quality of evidence) [2]. The Canadian Critical Care Clinical Practice guidelines recommend metoclopramide as the first-line prokinetic agent in the ICU [9]. However, in Chinese guidelines, herbal or natural medicines that enhance gastric motility are recommended for patients receiving gastric feeding tubes [10]. There is little agreement on how to use prokinetics for gastric feeding intolerance in critically ill patients. One of the reasons for the different recommendations may be that the definition of feeding intolerance has changed over time, especially regarding the index of high GRV. Some studies have suggested that measurement of GRV provides no benefit and should no longer be recommended. However, GRV is also an indicator of feeding intolerance in many ICUs, especially in patients with a high risk of aspiration and aspiration pneumonia. Therefore, the Chinese guidelines call for caution in abandoning monitoring of GRV in some high-risk patients [10]. If GRV

is measured, a volume of less than 500 mL should not result in an interruption of feeding unless there are other signs of intolerance, such as nausea, vomiting, abdominal pain, abdominal distension, or deterioration in hemodynamics or overall status [11]. A GRV of 500 ~~ml~~mL is the recommended threshold for a diagnosis of enteral feeding intolerance in US and European critical care and nutrition society guidelines [2, 3, 12]. Although the updated European Society for Clinical Nutrition and Metabolism (ESPEN) guidelines [3], published in 2019, provide the latest information on enteral nutrition (EN) and parenteral nutrition (PN) in critically ill adult patients, we find that some aspects of the efficacy and safety of prokinetics in critically ill patients are still quite unclear [13], and it is necessary to find new evidence to address these uncertainties.

On this topic, a previous meta-analysis by Lewis, K. et al. [14] examined the effects of prokinetics on feeding intolerance or high GRV and clinical outcomes. However, Lewis, K. et al. [14] defined feeding intolerance as GRV ≥150 ~~ml~~mL, vomiting, or abdominal distention resulting in feeding interruption. This definition may be considered obsolete [15]. Some new evidence has ~~come to light~~emerged on this topic; ~~taking~~ considering recent evidence~~ into consideration~~, we conducted this systematic review to determine the efficacy and safety of prokinetics for intolerance of gastric feeding in critically ill adult patients.

Methods

This systematic review and meta-analysis was conducted according to the Cochrane Handbook for Systematic Reviews of Interventions (version 5.1.0) [16], and the reporting of our study was based on the Preferred Reporting Items for Systematic Reviews and Meta-analyses (PRISMA) statement [17]. The review protocol is available on PROSPERO, registration number CRD42020157446. ~~The review protocol has been submitted to PROSPERO (ID 157446)~~, ~~b. But the review is still ongoing.~~ ~~https://www.crd.york.ac.uk/prospero/#recordDetails.~~

Neither patients who received gastric feeding in the ICU nor their families were involved in defining the research question or the outcome measures, but they were intimately involved in the design,

giving our team a wealth of good advice regarding design ideas.

Search strategy~~trials~~

We searched the Medline and Embase databases as well as the Cochrane Central Register of Controlled Trials (CENTRAL) from their inception dates to November 22, 2019. We combined Medical Subject Headings (MeSH) and free text terms to identify relevant articles. An information expert (XY) developed our search strategies.

We also searched clinicaltrials.gov (https://clinicaltrials.gov/) and the WHO ICTRP (http://apps.who.int/trialsearch/) for additional information, using the terms “critically ill patients”, and limited our search to studies labeled “completed” AND “Interventional studies (clinical trials)” in which summary results were available to identify additional eligible studies. There were no language restrictions. Additionally, we used a manual search strategy to retrieve the relevant articles referred to by the retrieved publications (the search strategies are reported in **S1 Table**).

Inclusion criteria

Trials were selected based on the following inclusion criteria: (1) the study was designed as a randomized controlled trial (RCT) comparing prokinetic treatment~~s~~ with ~~the~~ a control group; (2) the population included critically ill adult patients aged ≥18 years who were admitted to the ICU and received gastric feeding tube~~,~~s regardless of whether they had pre-existing feeding intolerance; (3) the intervention group received metoclopramide, erythromycin, or other prokinetic agents, such as herbal medicine or natural medicines with the function of enhancing gastric motility, regardless of the dose, frequency, duration or combination of prokinetics; (4) the control group received no intervention or a placebo; (5) if the gastric feeding patients with feeding intolerance had a GRV ≥500 mL and/or symptoms of nausea, vomiting, abdominal distention, regurgitation, deterioration in hemodynamics or other symptoms resulting in feeding interruption~~,~~ and ~~if they~~ failed to respond to interventions, ~~then,~~

regardless of whether they were in the control group or the prokinetics group, they were switched to postpyloric feeding or had gastric feeding withheld for 4-6 h [2, 3]; and (6) the outcomes included any of the following: all-cause mortality; Acute Physiology and Chronic Health Evaluation II (APACHE II) or Simplified Acute Physiology Score II; sepsis; use of an artificial airway; pneumonia; hospital or ICU length of stay; patient nutritional status (malnutrition); gastrointestinal symptoms; GRV; feeding intolerance; or ~~the~~ side effects of the prokinetics, such as cardiovascular disorders, bronchospasm, extrapyramidal symptoms, abdominal cramps, allergic reactions and pancreas disorders. The exclusion criteria were as follows: (1) the studies had no control group; (2) the studies had no prokinetic treatment group; (3) patients were considered to have feeding intolerance if tube feeding was electively not prescribed or was stopped/interrupted for procedural reasons; (4) the studies discontinued or interrupted the gastric feeding prematurely when the GRV was less than 500 mL or the patients did not have any signs of intolerance, such as nausea, vomiting, abdominal pain, abdominal distension, or deterioration in hemodynamics or overall status.

For our purposes, gastric feeding intolerance was defined as a “large” GRV (≥500 ~~ml~~mL), the presence of gastrointestinal symptoms (vomiting, diarrhea, gastrointestinal bleeding, the presence of enterocutaneous fistulas), or inadequate delivery of EN (the energy provided by EN was less than 20 kcal/kg BW/day after 72 h of feeding attempts or less than 60% of the EN target at the fifth day) in critically ill adults receiving gastric feeding tubes. Preventive usage of prokinetics meant that prokinetics were prescribed preemptively on the day EN was initiated and before patients presented a GRV >150 ~~ml~~mL or symptoms of feeding intolerance. Preventive usage of prokinetics for risk meant that prokinetics were used in patients with GRVs between 150 and 500 mL but before the development of intolerance. Therapeutic usage of prokinetics meant that the prokinetics were administered in patients who had developed feeding intolerance.

A reported adverse event was defined as any untoward medical occurrence or unfavorable and unintended sign, including an abnormal laboratory finding, symptom, or disease (new or exacerbated), temporally associated with the use of the study medication. The reported adverse events included

abnormal laboratory test results (hematology, clinical chemistry, or urinalysis) or other safety assessments (e.g., ECGs, radiological scans, or measurements of vital signs), including those that worsened from baseline and were deemed clinically significant in the medical and scientific judgment of the investigator; exacerbation of a chronic or intermittent preexisting condition, including an increase in the frequency and/or intensity of the condition; new conditions detected or diagnosed after the administration of study medication even if they may have been present prior to the start of the study; and/or signs, symptoms, or clinical sequelae of a suspected interaction, such as diarrhea, nosocomial pneumonia, severe sepsis, brain herniation, cardiac arrest, or changes in the electrocardiographic QTc interval.

Risk-of-bias assessments

The methodological quality for the included RCTs was assessed independently by 2 researchers (RP, HLL) based on the Cochrane risk-of-bias criteria [16]. The seven items used to evaluate bias in each trial included randomization sequence generation, allocation concealment, blinding of participants and personnel, blinding of outcome assessment, incomplete outcome data, selective reporting, and other bias. We defined other bias as being present in the trials where baseline characteristics were not similar between different intervention groups. The included trials were graded as low quality, high quality, or unclear risk based on the following criteria: (1) trials were considered low quality if either randomization or allocation concealment was assessed as having a high risk of bias, regardless of the risk of other items; (2) trials were considered high quality when both randomization and allocation concealment were assessed as having a low risk of bias and all other items were assessed as having a low or unclear risk of bias; (3) trials were considered to have unclear risk if they did not meet the criteria for high or low risk.

Data extraction

Two researchers (RP, HLL) independently extracted the following information from each eligible

RCT: (1) general study characteristics: author name, year of publication, numbers of treatment groups and patients, trial registry number, methods for measuring gastric emptying or GRV, and the definition of feeding intolerance; (2) patient characteristics: sex, age, baseline patient information (presence or absence of pre-existing feeding intolerance, APACHE II score and nutritional status, if reported); (3) primary diseases (the medical, surgical, or neurosurgical conditions of the critically ill patients); (4) interventions: details of the prokinetic treatment group and control group (e.g., dose, frequency, duration and combination of prokinetics for treatment); and (5) the outcomes: gastrointestinal symptoms, and feeding tolerance, the number of participants with all-cause death, the ICU length of stay, the hospital length of stay, and the number of reported adverse events.

If the trials had more than 2 groups or used factorial designs and could be analyzed using multiple comparisons, we extracted only the information and data of interest reported in the original articles. If a trial had multiple reports, we collated all data into one study. If a trial had both reports from ClinicalTrials.gov and journal publications, we carefully checked data from these two sources for consistency. If outcome data were reported at multiple follow-up points, we used data from the longest follow-up.

Statistical analysis

The effect of prokinetics on gastrointestinal symptoms and feeding tolerance, main clinical outcomes of all-cause mortality, ICU length of stay, hospital length of stay, and reported adverse events were analyzed. We recorded data on the number of participants with each outcome event by allocated group and recorded the number of participants with compliance and the participant, who was later thought to be eligible or otherwise excluded from treatment or follow-up. Intention-to-treat (ITT) analysis was conducted. ITT analysis is a comparison of the treatment groups that include all patients as originally allocated after randomization regardless of whether treatment was initiated or completed [18]. The CONSORT (Consolidated Standards of Reporting Trials) recommends ITT analysis as standard practice [19].

We performed a meta-analysis to calculate relative risks (RRs) or absolute risk differences (ARDs) in dichotomous data and mean differences (MDs) in continuous data, 95% CIs using the Mantel-Haenszel method and the inverse variance statistical method, respectively. If sufficient data were not available in the published reports or the abstract of the conference, we contacted the authors of the paper. If the raw data were not the mean and standard deviation, the sample mean and standard deviation were estimated from the sample size, median, range and/or interquartile range [20, 21].

We tested for heterogeneity between trial results using a standard Chi^2 test, and statistical heterogeneity between summary data was evaluated using the I^2 statistic. Sensitivity analysis was performed by excluding low-quality studies, trials recruiting participants with particular conditions, or trials with characteristics different from the others. When an inconsistency was detected between the RR and ARD for the same outcome, we explained the results based on the RR because the RR model is more consistent than ARD, particularly for an intervention aimed at preventing an undesirable event [16, 22].

In our meta-analysis, a random-effects model was used. The defining feature of the random-effects model is that there is a distribution of true effect sizes, and there are two sources of variance, within-study error variance and between-study variance [23]. However, if the number of studies is very small, the statistical power will have poor precision due to the variance between studies. Although the random-effects model is still the appropriate model, the information to apply it correctly is not available. In this case, we will add the separate effects to our manuscript. If heterogeneity was identified (I^2 >40% [16]) and ~~there were~~ sufficient trials were included in the review, we planned to investigate heterogeneity in the specified subgroups based on types of prokinetics (erythromycin, metoclopramide or other prokinetics), combination of prokinetics (yes or no), and feeding intolerance history (participants with or without pre-existing feeding intolerance before the start of the trial). Analysis was performed to assess whether the difference between the subgroups was statistically significant.

We assessed publication bias by examining funnel plots when the number of trials reporting the primary outcomes was 10 or more. However, if the number of included studies is less than 10 for a given main outcome, the funnel plot may not reliably detect evidence of departure bias. A prototypical situation that should elicit suspicion of publication bias ~~occurs~~is when evidence is derived ~~come~~ from a small number of studies or small sample size~~,~~s and all outcomes favor~~ed~~ the intervention [24]. All meta-analyses were performed using RevMan version 5.3 (Cochrane Collaboration). All tests were 2-tailed, and P <0.05 was considered statistically significant.

We used the Grading of Recommendations Assessment, Development, and Evaluation (GRADE) methodology to rate the certainty of evidence as high, moderate, low, or very low. RCTs begin as high-certainty evidence but can downgraded because of risk of bias, imprecision, inconsistency, indirectness, or publication bias. If the limitation of the evidence was considered ~~as~~ serious, the evidence was downgraded by one level; if the limitation was considered very serious, the evidence was downgraded by two levels [25].

Results

Our initial search identified a total of 595 citations. After deduplication, 459 publications remained. The titles and abstracts of those records were screened for inclusion, and 48 reports proved potentially eligible. After full-text screening, fifteen trials met the inclusion criteria [26-40]. ~~There were five~~Five studies ~~that~~ did not provide useful data for quantitative synthesis (meta-analysis) [36-40]. Ultimately, 10 trials were included in the quantitative analysis [26-35]. A total of 846 patients were enrolled in 10 RCTs, ~~that~~ includ~~ed~~ing a variety of critically ill patients with medical, surgical, and neurosurgical conditions. The details of the eligible trials are presented in **Fig 1**. ~~Reasons for excluding S~~Sstudies ~~included that the study~~were excluded if they had a different trial design [41-45], ~~; the study had~~ a different intervention or a different control [46-64], or ~~; the study had~~ a different population [65-67]~~;~~ or ~~the study~~ had been registered with the Clinical Trials Registry Platform (clinicaltrials.gov or WHO

ICTRP) and had been labeled “completed”~~,~~ but outcomes ~~did~~were not reported [68-73] (**S2 Table**).

Fig 1. Literature Search and Screening Process.

The 15 eligible studies reported 10 prokinetics, including metoclopramide, erythromycin, cisapride, GSK962040, mosapride and herbal medicine or natural medicines with the function of enhancing gastric motility (Chenxia Sijunzi decoction, ginger, fenugreek seed powder, gastrolit (*Zataria multiflora*), rikkunshito), respectively. Based on the outcomes measured, the studies were subdivided into those investigating effects on gastrointestinal symptoms, ~~and~~ feeding tolerance studies, and clinical outcome studies: hospital length of stay, ICU length of stay, reported adverse events, and all-cause mortality. The details of the eligible studies are presented in **Table 1**.

Table 1. Characteristics of the iIncluded Ttrials and Pparticipants

Included Trials	Population	Treatment #	Main outcomes	Definition of feeding intolerance †	When begin to use of Pprokinetics initiation timing *
Yavagal et al 2000 (India) [35]	ICU patients required placement of a nasogastric tube for >24 hrs. Mean age: 36.22 years, 61.97-% male. Mean APACHE II score: 17.54.	1) Metoclopramide 10 mg NG q8h; 2) Placebo.	1) Nosocomial pneumonia; 2) Mortality.	NA	Preventive usage
Sustic et al 2005 (Croatia) [39] ¶	Patients treated at a cardiosurgical ICU after CABG surgery, enteral feeding by nasogastric tube. Mean age: 59.5 years, 77.5 %% male. Mean SAPS II score: 21.	1) Metoclopramide 10 mg i.v.,; 2) Control group.	1) t_{+15}, t_{+30}, t_{+60}, t_{+120}; 2) AUC_{120}; 3) C_{max}.	NA	Preventive usage
Nursal et al 2007 (Turkey) [32]	Traumatic brain injury patients with Glasgow Coma Scores of 3–11. eEnteral feeding by nasogastric tube. Mean age: 43.42 years, 84.2 %% male Mean APACHE II score: 12.87.	1) Metoclopramide 10 mg i.v. q8h×5 days; 2) Control group, saline	1) Feeding intolerance; 2) Feeding complications; 3) AUC_{60}; 4) C_{max}; 5) Length of hospital stays; 6) Mortality.	Gastrointestinal symptoms (without GRV)	Preventive usage
Nassaji et al 2010 (Islamic Republic of lran) [31]	Surgical ICU with a nasogastric tube for more than 24 hours. Mean age: 44.88 years, 65.45 %% male. Mean APACHE II score: Nnot reported.	1) Metoclopramide 10 mg NG q8h; 2) Control patients did not receive metoclopramide.	1) Nosocomial pneumonia; 2) Mortality.	NA	Preventive usage

Acosta-Escribano et al 2014 (Spain) [26]	Adult neuro-critical patients, Glasgow Coma Scores of 14 ~~<~~to 9 points, with ventilation indications ~~mechanics~~ at admission and the need for ~~nutrition~~ artificial enteral nutrition. Mean age: 54.53 years, 65.14 ~~%~~% male. Mean APACHE II score: 18.53.	1) Metoclopramide 10 mg;<br>2) Placebo.	1) Gastrointestinal complications;<br>2) Gastric residue;<br>3) Mechanical ventilation-associated pneumonia;<br>4) The duration of mechanical ventilation;<br>5) Length of ICU stay;~~s~~<br>6) Length of hospital stay;~~s~~<br>7) Mortality.	Large GRV alone (>500 ~~ml~~mL in two consecutive episodes)	Preventive usage
Rajan et al 2017 (India) [38] ¶	Critically ill ~~cirrhotics~~cirrhotic patients in ~~L~~a liver ICU with feeding intolerance.	1) Metoclopramide i.v.,<br>2) Erythromycin i.v.,<br>3) Placebo.	1) Mortality;<br>2) GRV.	Gastrointestinal symptoms including large GRV (500 ~~ml~~mL)	Therapeutic usage
Ritz et al 2005 (Australia) [33]	Mixed medical/surgical intensive care unit patients with mechanic~~ally~~ ~~ventilat~~ventilation~~ed~~. Mean age: 47.49 years, 60.9 ~~%~~% male. Mean APACHE II score: 19.	1) **Erythromycin 70 mg;**<br>2) Erythromycin 200 mg;<br>3) Placebo, saline (0.9%).	1) Gastric emptying coefficient;<br>2) Gastric half-emptying time ($t_{1/2}$).	NA	Preventive usage
Spapen et al 1995 (Belgium) [34]	Adult medical/surgical intensive care unit patients~~,~~ requiring prolonged mechanical ventilation and enteral feeding. Mean age: 71.10 years, 52.38 ~~%~~% male. Mean APACHE II score: not reported.	1) Cisapride 10 mg q6h;<br>2) No treatment.	1) Gastric residue;<br>2) The mean time at which 50% of the technetium 99m-labeled test meal was eliminated from the stomach ($T_{1/2}$);<br>3) Mortality.	NA	Preventive usage

Heyland et al 1996 (Canada) [36] ¶	Mechanically ventilated patients in ~~ICU of~~ trauma and neurosurgery ICUs. Mean age: 53.9 years, 61 ~~%~~% male. Mean SAPS score: 9.5.	1) Cisapride 20 mg, NG;<br>2) An identical placebo.	1) C_{max};<br>2) AUC_{180}.	NA	Preventive usage
Chapman et al, 2016 (Australia) [27]	Patients undergoing invasive mechanical ventilation in the ICU with nasogastric feeding. Mean age: 44.67 years, 83.33 ~~%~~% male. Mean APACHE II score: 18.14.	**1) GSK962040 (50 mg);**<br>2) GSK962040 (75 mg);<br>3) Placebo.	1) Breath test gastric time to half emptying (BTt½);<br>2) Gastric emptying coefficient;<br>3) AUC_{240}, AUC_{60};<br>4) C_{max};<br>5) Adverse events.	Large GRV alone (>200 ~~ml~~mL) at least 6 hours after ~~ommencing~~commencing liquid nutrition~~ent~~ at ≥ 40 kcal/hr	Preventive usage for risk
Mokhtari et al 2009 (Islamic Republic of lran) [37] ¶	Adult respiratory distress syndrome (ARDS) ICU patients.	1) Ginger,<br>2) Placebo.	1) Feeding tolerated;<br>2) Ventilator-associated pneumonia;<br>3) ICU-free days;<br>4) Ventilator-free days;<br>5) Morality.	Delayed gastric emptying is one of the major reasons for enteral feeding intolerance	Preventive usage
Guo JH, et al 2012 (China) [29]	Feeding with enteral nutrition in critically ill patients. Mean age: 59.49 years, 53.33 ~~%~~% male. Mean APACHE II score: ~~N~~not reported.	1) **Traditional Chinese medicine group: Chenxia Sijunzi decoction**;~~,~~<br>2) Western medicine group: mosapride dispersible tablets 5 mg and multienzyme tablets NG ~~.~~;<br>3) Control group: routine symptomatic~~ally~~ treatmented without any medicines ~~for~~to promote~~ing~~ gastrointestinal ~~power~~ function.	1) The time of bowel sound recovery;<br>2) Gas passage time by anus;<br>3) The bowel movement time;<br>4) ~~The D~~Days in the hospital.	NA	Preventive usage

Kooshki et al 2018 (Iran) [30]	Mechanically ventilated patients, enteral nutrition with nasogastric tube in two intensive care unit centers. Mean age: 56.95 years, 51.67% male. Mean APACHE II score: 23.2.	1) Fenugreek seed powder 3 g q12h NG;<br>2) Routine care.	1) Diarrhea;<br>2) Constipation;<br>3) GRV;<br>4) Respiratory aspiration;<br>5) Duration of mechanical ventilation;<br>6) Length of stay in the hospital;<br>7) Length of stay in the ICU;<br>8) Mortality.	Gastrointestinal symptoms	Preventive usage
Tahershamsi et al 2018 (Iran) [40] ¶	Mechanically ventilated patients hospitalized in ICU. Mean age: 63.06 years, 60.0 ~~%~~% male. Mean APACHE II score: No report.	1) Gastrolit (*Zataria multiflora*) (20 drops) q8h× 4 days;<br>2) Placebo = water.	1) GRV.	NA	Preventive usage
Doi et al 2019 (Japan) [28]	Critically ill adult patients requiring enteral nutrition by gastric tube for at least 5 days, and all patients were treated with invasive mechanical ventilation. Mean age: 72.84 years, 77.78% male. Mean APACHE II score: 22.82.	**1) Rikkunshito 5 g** q8h × 5 days;<br>2) Rikkunshito 2.5 g q8h× 5 days;<br>3) No rikkunshito (control).	1) GRV;<br>2) The percentage of the target enteral calorie intake achieved at the fifth day;<br>3) The plasma levels of ghrelin;<br>4) ICU length of stay;<br>5) Hospital length of stay;<br>6) Adverse events;<br>7) Mortality.	Inadequate enteral nutrition/failure to meet the ~~of the~~ enteral nutrition target at the fifth day (<60%)~~.~~	Preventive usage

NG: nasogastric tube feeding; i.v.: intravenous injection; NA: not applicable; C_{max}: ~~P~~peak paracetamol plasma levels; AUC: the area under the paracetamol concentration curve; t_{+15}, t_{+30}, t_{+60}, t_{+120}: ~~P~~plasma paracetamol concentrations at 15, 30, 60, and 120 minutes after administration of paracetamol and saline or metoclopramide in patients; SAPS, simplified acute physiology score; GRV, gastric residual volume.~~;~~

# If the trials had more than 2 groups or factorial designs and permitted multiple comparisons, the subgroup ~~with the~~in bold font was extracted in this study.

¶ The study did not provide useful ~~dada~~data for ~~M~~meta-analysis.

Risk of bias

There was one trial at low risk of bias [27], and two studies were at high risk of bias [28, 31] due to inappropriate randomization and/or allocation concealment. For the remaining 12 studies, we were unable to comprehensively evaluate the risk of bias due to a lack of information [26, 29, 30, 32-40]. **(S1 Fig)**.

Publication bias

We checked the funnel plots of the main outcomes for asymmetry as planned; however, we included less than 10 RCTs in each main outcome, such that the funnel plots may not reliably detect evidence of departure bias.

Main outcomes

Effect on gastrointestinal symptoms and feeding tolerance

Thirteen studies ~~have~~ evaluated the effect of prokinetics on gastrointestinal symptoms and/or feeding tolerance in adult critically ill patients receiving gastric feeding [26-30, 32-34, 36-40]. The main results obtained are as follows: gastric emptying, GRV, diarrhea, constipation, feeding complications and feeding intolerance. Gastric emptying was measured by the drug model of acetaminophen absorption or the 13C-octanoic acid breath test with calculation of the~~ng~~ gastric emptying time, gastric emptying coefficient or area under the plasma concentration-time curve. The various outcome definitions, especially for gastric tube tolerance, precluded quantitative synthesis of the data.

As a class of drugs, prokinetic agents appear to have positive effects on gastrointestinal function and improving feeding tolerance. Ten of the thirteen studies reported ~~the~~ positive effects on improving gastric emptying and/or resolution of feeding intolerance in critically ill patients with the use of~~by~~

using prokinetic agents. However, two studies suggested that metoclopramide had no effect on decreasing gastrointestinal complications in adult neurocritical patients or critical traumatic brain injury patients. One study reported that rikkunshito did not improve the achievement of enteral calorie targets in critically ill adults **(Table 2)**.

Table 2. Effects on gastrointestinal symptoms and feeding tolerance

Study	Population (sample size)	Intervention	Outcome	*P* Value	Conclusions
Sustic et al 2005 (Croatia) [39]	Cardiosurgical patients after CABG surgery (40)	1) Metoclopramide 10 mg i.v.; 2) Control group: saline.	AUC_{120}; $C_{max.}$ 574±296; 8.51±2.2 429±309; 5.15±2.8	0.027; 0.007	In CABG surgery patients with early enteral feeding, a single dose of intravenous metoclopramide effectively improves gastric emptying.
Nursal et al 2007 (Turkey) [32]	Traumatic brain injury patients with Glasgow Coma Scale scores of 3–11 (19)	1) Metoclopramide 10 mg i.v. q8h×5 days; 2) Control group: saline.	FI; feeding complications; AUC_{60} at day 5; C_{max} day 5; 4/10 (40%); 5/10 (50%); 589.6±457.8; 15.8±12.9 2/9 (22.2%); 3/9 (33.3%); 560±432.9; 12.0±9.9	NS; NS; NS; NS	The results were unable to documentreveal any advantage toof using metoclopramide in TBI patients.
Acosta-Escribano et al 2014 (Spain) [26]	Adult neuro-critical patients, Glasgow Coma Scores of 14 <to 9 points (109)	1) Metoclopramide 10 mg i.v.; 2) Placebo: saline.	Incidence of gastrointestinal complications; Incidence of GRV>500 mlmL at day 5; 29/58 (50%); 16/58 (28%) 22/51 (45%); 11/51 (22%)	NS; NS	The MMmetoclopramide has no effect on decreasing of the gastrointestinal complications in adult neuro-critical patients

Rajan et al 2017 (India) [38]	Critically ill ~~cirrhotics~~cirrhotic patients in ~~L~~a liver intensive care unit (72)	1) Metoclopramide i.v.;~~,~~<br>2) Erythromycin i.v.~~,~~;<br>3) Placebo.	Resolution of FI; Decrease in GRV beyond 24 hrs; the time to restart enteral nutrition (days)<br>8.7%; no report; 2.61±0.72<br>24%; no report; 2.20±0.91<br>~~N~~no report; no report; 3.47±1.29	0.026; no report; 0.03	Early detection and the addition of prokinetics ~~helps in~~facilitate the resolution of FI in critically ill cirrhotic patients~~s~~. Erythromycin is safe and superior to ~~M~~metoclopramide for early resolution of gut paralysis in critically ill cirrhotic patients.~~s.~~
Ritz et al 2005 (Australia) [33]	Mixed medical/surgical intensive care unit patients (35)	1) **Erythromycin 70 mg i.v.;**<br>2) Erythromycin 200 mg i.v.;<br>3) Placebo, saline (0.9%).	Gastric emptying coefficient; gastric half-emptying time ($t_{1/2}$)<br>3.8 (3.3-4.0); 98 (88-112) min<br>4.0 (3.6-4.2); 86 (75-104) min<br>2.9 (2.5-3.7); 122 (102-190) min	<0.05; <0.05	Treatment with 70 and 200 mg of intravenous erythromycin ~~are~~is equally effective in accelerating gastric emptying in ~~the~~ critically ill patients. Doses as low as 70 mg (approx. 1 mg/kg) accelerate gastric emptying in ~~the~~ critically ill patients, improving the success of enteral feeding. This effect is ~~seen~~observed only in patients with delayed gastric emptying.

Spapen et al 1995 (Belgium) [34]	Adult medical/surgical intensive care unit patients (21)	1) Cisapride 10 mg q6h NG;<br>2) No treatment.	Gastric residue over one-week; gastric emptying time $T_{1/2}$;<br>17.7±8.9 ~~ml~~mL; 18±7 min<br>94.5±33.3 ~~ml~~mL; 78±40 min	<0.001; <0.005	Gastric emptying in critically ill, sedated, and mechanically ventilated patients can be significantly improved by adding cisapride to a routine enteral feeding protocol.
Heyland et al 1996 (Canada) [36]	Mixed intensive care unit patients (72)	1) Cisapride 20 mg;<br>2) An identical placebo.	~~The D~~Ddifferences (Day 2 - Day 1) ~~of~~in ΔC_{max}; Δt_{max}; ΔAUC_{180}<br>49.1±10.7; -40.8±12.0; 5534±1349<br>12.3±7.0; -4.2±10.4; 2832±769	0.005; 0.02; 0.09	Cisapride enhances gastric emptying in critically ill patients
Chapman et al, 2016 (Australia) [27]	Mixed intensive care unit patients (33)	**1) GSK962040 (50 mg) NG;**<br>2) GSK962040 (75 mg) NG;<br>3) Placebo.	Baseline vs. post gastric emptying time BTt½; AUC_{240}<br>0.65 (0.39,0.1.08); 2.50 (1.68,3.72)<br>1.85 (0.82,4.15); 0.72 (0.39,1.36)<br>1.21 (0.68,2.15); 1.33(0.85,2.06)	No report; no report	A single enteral dose of camicinal (50 mg), but not 75 mg, accelerates gastric emptying and increases glucose absorption in feed-intolerant critically ill patients.
Mokhtari et al 2009 (Islamic Republic of Iran) [37]	Critically ill a~~A~~dult respiratory distress syndrome (ARDS) ~~critically ill~~ patients (32)	1) Ginger NG,<br>2) Placebo.	Feeding tolerated in the first 48 hrs; feeding tolerated during the entire study period<br>51%; 92%<br>57%; 93%	<0.005; 0.42	Supplementing the diet with ginger extract in ARDS patients reduces the delayed gastric emptying risk.

Guo JH, et al 2012 (China) [29]	Mixed intensive care unit patients (80)	1) **Traditional Chinese medicine group: Chenxia Sijunzi decoction;**<br>2) Western medicine group: mosapride dispersible tablets 5 mg and multienzyme tablets NG;<br>3) Control group: routine symptomatic~~ally~~ treatment~~ed~~ without any medicines ~~for~~to promot~~inge~~ gastrointestinal ~~power~~ function.	The time ~~of~~to bowel sound recovery; the time ~~of~~to passage of gas by anus recovery; the time to~~of the~~ bowel movement recovery<br>41.02±7.52^a; 49.90±6.95^a; 58.22±6.71^a<br>44.02±6.23^a; 51.32±5.12^a; 60.91±3.72^a<br>54.62±5.51; 64.68±9.47; 78.20±7.11	^aP<0.01	Chenxia Sijunzi decoction can promote severe patient~~`~~s' gastrointestinal function recovery. ~~And there were not~~No ~~any~~ significant differences in each testing index were found between the traditional Chinese medicine and ~~w~~Western medicine groups.
Kooshki et al 2018 (Iran) [30]	Mixed intensive care unit patients (60)	1) Fenugreek seed powder 3 g q12h NG;<br>2) Routine care.	GRV at the 5th day; diarrhea; constipation; respiratory aspiration at 5th/6th days<br>28.06±9.23; 1/30 (3.3%); 3/30 (10%); 1/30 (3.3%)<br>38.94±9.54; 6/30 (20%); 21/30 (70%); 10/30 (33.3%)	0.001; 0.04; 0.001; 0.005	~~The B~~beneficial effects of fenugreek seeds on food intolerance in critically ill patients.

Tahershamsi et al 2018 (Iran) [40]	Mixed intensive care unit patients (50)	1) Gastrolit (Zataria multiflora) (20 drops) q8h× 4 days;<br>2) Placebo = water.	GRV on the second, third, and fourth days<br>The data ~~did~~could not be extracted	All P<0.0001	Gastrolit can decrease GRV in mechanically ventilated patients
Doi et al 2019 (Japan) [28]	Mixed intensive care unit patients	**1) Rikkunshito 5 g** q8h ×5 days;<br>2) Rikkunshito 2.5 g q8h×5 days;<br>3) No rikkunshito (control).	GRV; the percentage of the target energy at the 5th day; ~~achieved~~ the target energy was achieved at the 5th day<br>No report; 62%; 63%<br>No report; 40%; 38%<br>No report; 59%; 56%	NS; NS; NS	Standard- or high-dose rikkunshito did not improve ~~the~~ achievement of the enteral calorie target in critically ill adults.

FI, feeding intolerance; NS, not significant; [a]*P*<0.01~~,~~ compared with the control group.

Effect on hospital or ICU length of stay

The effect of prokinetics on hospital length of stay ~~has been~~was examined by five studies [26, 28-30, 32]. Th~~o~~ese five studies, which enroll~~ing~~ed a total of 250 patients, demonstrated ~~that there was~~ a significant difference in the hospital length of stay between the ~~use of~~ prokinetic agent-treated group~~s~~ and the control group (MD -3.21, 95% CI -5.35, -1.06; P = 0.003; I^2 = 28~~-~~%) (**Fig 2**). Three studies evaluated the effect of prokinetics on ICU length of stay in the critical care setting [26, 28, 30]. These three studies, enrolling a total of 186 patients, showed that the prokinetic agents appear to have a positive effect on shortening ICU length of stay (MD -2.03, 95% CI -3.96, -0.10; P = 0.04; I^2 = 0%) **(Fig 3)**. Additionally, the separate effects of different prokinetics on the ICU length of stay and hospital length of stay are presented in **S3 Table**.

Fig 2. Forest plot for hospital length of stay outcomes. IV: inverse variance; CI: confidence interval.

Fig 3. Forest plot for ICU length of stay outcomes. IV: inverse variance; CI: confidence interval.

Effect on reported adverse events

Seven studies reported events that met the definition of adverse events in 757 critically ill patients [26-28, 30-32, 35]. The meta-analysis showed ~~that there was~~ no significant difference in the risk of reported adverse events between the prokinetic agent group and the control group (RR 1.13, 95% CI 0.92, 1.38; P = 0.25; I^2 = 0%) **(S2 Fig)**.

Effect on all-cause mortality

The effect of prokinetic agents on all-cause mortality has been examined by six studies in 691 critically ill patients [26, 28, 31, 32, 34, 35]. There was no significant difference in all-cause mortality between the prokinetic agent group and the control group (RR 0.96, 95% CI 0.81, 1.14; P = 0.64; I^2 = 0%) **(S3 Fig)**.

Subgroup analysis

Although no significant heterogeneity was found, we performed subgroup analyses to determine ~~if there are~~whether important subgroup differences existed. In the subgroup analysis stratified by type of prokinetic agents, ~~there were~~ no significant subgroup differences were detected in the clinical outcomes of hospital length of stay, ICU length of stay, reported adverse events and all-cause mortality (**S4, 5, 6 and 7 Figs**). Furthermore, ~~there was~~ no study ~~comparing~~ compared the combination of prokinetics to placebo or no treatment. Only one study about the preventive usage of prokinetics for risk patients demonstrated the outcome of reported adverse events [27]. The others were about the preventive usage of prokinetics for all patients. The subgroup analysis result of the preventive usage of prokinetics for all patients did not show important changes in the pooled effects of the reported adverse events.

Sensitivity analysis

The sensitivity analysis, which was performed by excluding the trials with a high risk of bias [28, 31], did not show important changes in the pooled effects of hospital length of stay, ICU length of stay, reported adverse events, or all-cause mortality. (Supplemental materials: **S8, 9, 10 and 11 Figs**).

Certainty of evidence

The certainty of evidence was moderate for the clinical outcome of all-cause mortality. However, the certainty of evidence was low for the clinical outcomes of ICU length of stay, hospital length of stay and reported adverse events. The details of the risk of bias and quality assessment are outlined in **Table 3**.

Table 3. GRADE evidence profile of the efficacy and safety of prokinetics in critically ill adult patients receiving gastric feeding tubes

Certainty assessment							No.~~№~~ of patients		Effect		Certainty	Importance
**No. ~~№~~ of studies**	**Study design**	**Risk of bias**	**Inconsistency**	**Indirectness**	**Imprecision**	**Other considerations**	**Prokinetics**	**Placebo**	**Relative (95% CI)**	**Absolute (95% CI)**		
**Effect on ICU length of stay**												
3	randomi~~s~~ze d trials	serious [1]	not serious [2]	not serious [3]	serious [4]	none [5]	96	90	-	MD **2.03 days lower** (3.96 **days** lower to 0.1 **days** lower)	⊕⊕◯◯ LOW	IMPORTANT
**Effect on hospital length of stay**												
5	randomi~~s~~ze d trials	serious [6]	not serious [7]	not serious [3]	serious [8]	none [5]	141	109	-	MD **3.21 days lower** (5.35 **days** lower to 1.06 **days** lower)	⊕⊕◯◯ LOW	IMPORTANT
**Effect on reported adverse events**												
7	randomi~~s~~ze d trials	serious [9]	not serious [10]	not serious [3]	serious [11]	none	105/320 (32.8%)	120/437 (27.5%)	**RR 1.13** (0.92 to 1.38)	**40 more RAE ~~more~~ per 1,000 patients** (from 20 fewer RAE ~~fewer~~ to 100 more RAE ~~more~~)	⊕⊕◯◯ LOW	IMPORTANT
**Effect on all-cause mortality**												
6	randomi~~s~~ze d trials	serious [12]	not serious [13]	not serious [3]	not serious [14]	none	114/286 (39.9%)	174/405 (43.0%)	**RR 0.96** (0.81 to 1.14)	**30 fewer deaths ~~fewer~~ per 1,000 patients** (from 100 fewer deaths ~~fewer~~ to 40 more deaths ~~more~~)	⊕⊕⊕◯ MODERATE	CRITICAL

CI: ~~C~~confidence interval; RR: ~~R~~risk ratio; MD: ~~M~~mean difference; RAE: ~~r~~reported adverse events.

1. We downgraded the quality of evidence for risk of bias by one level. Two of three included studies had a ~~were at~~ high or unclear risk of bias.
2. We did not downgrade for inconsistency, I^2=0% and Chi^2 = 1.45, P=0.48.

3. Although the studies included any critically ill patient, we did not downgrade for indirectness.

4. We downgraded the quality of -evidence for imprecision by one level~~,~~ because the total population size is less than 400. ~~A~~The~~nd~~ 95% confidence interval contained a small benefit that did not meet the clinical decision threshold (min. one day).

5. We did not downgrade for publication bias, although we could not assess this category reliably due to the small number of eligible studies. ~~Because~~ N~~n~~ot all ~~of~~ included studies ~~were~~ show~~ing~~ed benefits of the studied intervention.

6. We downgraded the quality of evidence for risk of bias by one level. Most studies had an unclear risk of bias. ~~Besides~~In addition, one study lacked ~~of~~ allocation concealment and blinding.

7. We did not downgrade for inconsistency, I^2=28% and Chi^2 = 5.52, P=0.24.

8. We downgraded the quality of evidence by one level for imprecision because~~,~~ the population size is less than 400.

9. We downgraded the quality of evidence for risk of bias by one level. Most studies had an unclear risk of bias. ~~Besides~~In addition, two studies lacked ~~of~~ allocation concealment and/or blinding.

10. We did not downgrade for inconsistency, I^2=0% and Chi^2 = 1.64, P=0.95.

11. We downgraded the quality of evidence for imprecision by one level~~,~~ because the 95% confidence interval around the pooled effect included both no effect and appreciable harm (~~the~~a relative risk increase greater than 25%).

12. We downgraded the quality of evidence for risk of bias by one level. Most studies had an unclear risk of bias. ~~Besides~~In addition, two studies lacked ~~of~~ allocation concealment, and two studies lacked ~~of~~ blinding.

13. We did not downgrade for inconsistency, I^2=0% and Chi^2 = 4.52, P=0.48.

14. We did not downgrade for imprecision~~,~~ because the 95% confidence interval around the pooled effect did not include both no effect and an appreciable benefit (~~the~~a relative risk reduction greater than 25%) or appreciable harm (~~the~~a relative risk increase greater than 25%).

Discussion

In this systematic review, we conducted a comprehensive literature search and used objective criteria for study inclusion. Fifteen studies were included in the final analysis. Because of small sample sizes and a relatively small amount of research, the accuracy of the pooled effect is lacking in quantitative analysis. ~~The majority of~~Most studies (10 of 13, 76.92%) found that prokinetic agents showed beneficial effects on feeding tolerance in critically ill adults. The negative studies (3 of 13, 23.08%) were hampered by special populations of neuro-critical ~~neurocritical~~ patients and critical traumatic brain injury patients ~~with~~ taking metoclopramide and by the use of the~~a~~ specific drug ~~of~~ rikkunshito. Furthermore, the use of prokinetic agents in critically ill patients receiving gastric feeding may reduce the ICU or hospital length of stay, but the certainty of evidence was low due to risk of bias and imprecision. Prokinetics did not significantly reduce the risks of reported adverse events or all-cause mortality.

In this study, we examined the effect of prokinetic agents on gastrointestinal symptoms, feeding tolerance and clinical outcomes. Compared to the control group, prokinetics did not reduce the risk of mortality; these results were the same as the results of the meta-analysis by Lewis, K. et al. [14], but our methods are different. Lewis, K. et al. [14] defined feeding intolerance as either GRV ≥150 ~~ml~~mL, vomiting, or abdominal distention resulting in feeding interruption. This definition may be considered obsolete [15]. We defined gastric feeding intolerance as either GRV ≥500 ~~ml~~mL or concomitant ~~with~~ symptoms of nausea, vomiting, abdominal distention, regurgitation or other symptoms resulting in feeding interruption in critically ill adult patients receiving gastric feeding tubes. We excluded studies that discontinued or interrupted gastric feeding prematurely following the disappearance of gastric feeding intolerance. Under this latest definition, our meta-analysis found some new studies [26-30, 33, 34], ~~besides,~~and we identified 5 studies regarding the administration of prokinetics ~~of~~ including herbal medicines/natural medicines in critically ill adult patients receiving gastric feeding tubes ~~in critically ill patients.adults~~ [28-30, 37, 40].

Additionally, we found that prokinetic agents might reduce the ICU or hospital length of stay ~~in~~ for critically ill patients receiving gastric feeding. However, the number of studies and the sample size were very small, and the certainty of evidence was low. Furthermore, ~~there was~~ no significant difference was found between prokinetic agent groups and placebo/no treatment in the risks of reported adverse events and all-cause mortality. Therefore, we cannot draw a convincing conclusion that the use of prokinetics ~~could~~ can improve clinical outcomes in critically ill adults. ~~We recommend a more comprehensive search and further original studies on this topic.~~ We recommend that more research ~~needs to~~should be conducted in this field.

This study has several limitations. First, RCTs from 21 published original studies or trials registered in the International Clinical Trials Registry Platform (WHO) or clinicaltrials.gov were identified. However, 6 trials were completed, but the results were not available, which might lead to the omission of trials meeting the inclusion criteria and might have publication bias. Second, some included trials did not test the baseline status of feeding intolerance for all participants. The subgroup results might have been different if all individuals were tested. Third, we were unable to comprehensively evaluate the risk of bias in 12 studies with a lack of information. Fourth, in each outcome, the total sample size was relatively small, which likely had inadequate power to detect a difference in treatment effect. We recommend that more original studies about this topic be conducted.

Conclusion

As a class of drugs, prokinetic agents may improve gastric feeding tolerance in critically ill adults. However, the~~re is very low~~ certainty ~~in~~ of the evidence suggesting that prokinetic agents are effective ~~in~~ for reducing the ICU or hospital length of stay is low. There was also no significant reduction in the risk of reported adverse events and all-cause mortality. Additional RCTs are needed to determine the effect of prokinetics on clinical outcomes in critically ill patients in the future.

Acknowledgments

We are grateful to all the staff in this study for their teamwork and persistent efforts, and we are also thankful to the Group of People with Highest Risk of Drug Exposure of the International Network for the Rational Use of Drugs, China, and the Evidence-Based Pharmacy Committee of the Chinese Pharmaceutical Association for providing methodologies.

Ethics approval and consent to participate

Not applicable.

Competing interests

We declare that we have no conflicts of interest.

Funding

The funders had no role in the study design, data collection and analysis, the decision to publish, or preparation of the manuscript.

Author Contributions

LLZ conceived the study and designed the study. RP and HLL had full access to all of the data in the study, and take responsibility for the integrity of the data and the accuracy of the data analysis. RP and HLL developed and tested the data collection form. All authors acquired the data. HLL and RP conducted the analysis, interpreted the data, and drafted the manuscript. All authors critically revised the manuscript. LLZ is the guarantor.

Consent for publication

Written informed consent for publication was obtained from all participants.

Availability of data and material

All data generated or analyzed during this study are included in this published article.

References

1. Lew CCH, Yandell R, Fraser RJL, Chua AP, Chong MFF, Miller M. Association Between Malnutrition and Clinical Outcomes in the Intensive Care Unit: A Systematic Review [Formula: see text]. *JPEN J Parenter Enteral Nutr.* 2017;41(5):744-758.

2. McClave SA, Taylor BE, Martindale RG, Warren MM, Johnson DR, Braunschweig C, McCarthy MS, Davanos E, Rice TW, Cresci GA, Gervasio JM, Sacks GS, Roberts PR, Compher C, Society of Critical Care M, American Society for P, Enteral N. Guidelines for the Provision and Assessment of Nutrition Support Therapy in the Adult Critically Ill Patient: Society of Critical Care Medicine (SCCM) and American Society for Parenteral and Enteral Nutrition (A.S.P.E.N.). *JPEN J Parenter Enteral Nutr.* 2016;40(2):159-211.

3. Singer P, Blaser AR, Berger MM, Alhazzani W, Calder PC, Casaer MP, Hiesmayr M, Mayer K, Montejo JC, Pichard C, Preiser JC, van Zanten ARH, Oczkowski S, Szczeklik W, Bischoff SC. ESPEN guideline on clinical nutrition in the intensive care unit. *Clin Nutr.* 2019;38(1):48-79.

4. Elke G, Felbinger TW, Heyland DK. Gastric residual volume in critically ill patients: a dead marker or still alive?. [Review].1.

5. Blaser AR, Starkopf J, Kirsimagi U, Deane AM. Definition, prevalence, and outcome of feeding intolerance in intensive care: a systematic review and meta-analysis. *Acta Anaesthesiol Scand.* 2014;58(8):914-922.

6. Reintam Blaser A, Malbrain ML, Starkopf J, Fruhwald S, Jakob SM, De Waele J, Braun JP, Poeze M, Spies C. Gastrointestinal function in intensive care patients: terminology, definitions and management. Recommendations of the ESICM Working Group on Abdominal Problems. *Intensive Care Med.* 2012;38(3):384-394.

7. Hu B, Sun R, Wu A, Ni Y, Liu J, Guo F, Ying L, Ge G, Ding A, Shi Y, Liu C, Xu L, Jiang R, Lu J, Lin R, Zhu Y, Wu W, Xie B. Prognostic Value of Prolonged Feeding Intolerance in Predicting All-Cause Mortality in Critically Ill Patients: A Multicenter, Prospective, Observational Study. *JPEN J Parenter Enteral Nutr.* 2019.

8. Gungabissoon U, Hacquoil K, Bains C, Irizarry M, Dukes G, Williamson R, Deane AM, Heyland DK. Prevalence, risk factors, clinical consequences, and treatment of enteral feed intolerance during critical illness. *JPEN J Parenter Enteral Nutr.* 2015;39(4):441-448.

9. Heyland DK, Dhaliwal R, Drover JW, Gramlich L, Dodek P, Canadian Critical Care Clinical Practice Guidelines C. Canadian clinical practice guidelines for nutrition support in mechanically ventilated, critically ill adult patients. *JPEN J Parenter Enteral Nutr.* 2003;27(5):355-373.

10. Consensus of early enteral nutrition clinical practice in critically ill patients. *Chinese Critical Care Medicine.* 2018;30(8):715-721.

11. Taylor BE, McClave SA, Martindale RG, Warren MM, Johnson DR, Braunschweig C, McCarthy MS, Davanos E, Rice TW, Cresci GA, Gervasio JM, Sacks GS, Roberts PR, Compher C, Society of Critical Care M, American Society of P, Enteral N. Guidelines for the Provision and Assessment of Nutrition Support Therapy in the Adult Critically Ill Patient: Society of Critical Care Medicine (SCCM) and American Society for Parenteral and Enteral Nutrition (A.S.P.E.N.). *Crit Care Med.* 2016;44(2):390-438.

12. Reintam Blaser A, Starkopf J, Alhazzani W, Berger MM, Casaer MP, Deane AM, Fruhwald S, Hiesmayr M, Ichai C, Jakob SM, Loudet CI, Malbrain ML, Montejo Gonzalez JC, Paugam-Burtz C, Poeze M, Preiser JC, Singer P, van Zanten AR, De Waele J, Wendon J, Wernerman J, Whitehouse T, Wilmer A, Oudemans-van Straaten HM, Function EWGoG. Early enteral nutrition in critically ill patients: ESICM clinical practice guidelines. *Intensive Care Med.* 2017;43(3):380-398.

13. Peng R. The efficacy and safety of administration of prokinetics improve clinical outcomes in critically ill patients is still quite unclear. *Clinical Nutrition.* 2020;39:307-309.

14. Lewis K, Alqahtani Z, McIntyre L, Almenawer S, Alshamsi F, Rhodes A, Evans L, Angus DC, Alhazzani W. The efficacy and safety of prokinetic agents in critically ill patients receiving enteral nutrition: A systematic review and meta-analysis of randomized trials. *Critical Care.* 2016;20 (1) (no pagination)(259).

15. Dive A. Benefit of prokinetics during enteral nutrition: still searching for a piece of evidence. *Crit Care.* 2016;20(1):341.

16. Higgins JPT. Green S, eds. Cochrane Handbook for Systematic Reviews of Interventions, version 5.1.0 (updated March 2011). Cochrane Collaboration website. http://training.cochrane.org/handbook. 2011. Accessed November 22, 2017.

17. Liberati A, Altman DG, Tetzlaff J, Mulrow C, Gotzsche PC, Ioannidis JP, Clarke M, Devereaux PJ, Kleijnen J, Moher D. The PRISMA statement for reporting systematic reviews and meta-analyses of studies that evaluate health care interventions: explanation and elaboration. *J Clin Epidemiol.* 2009;62(10):e1-34.

18. Gupta SK. Intention-to-treat concept: A review. *Perspect Clin Res.* 2011;2(3):109-112.

19. Schulz KF, Altman DG, Moher D, Group C. CONSORT 2010 statement: updated guidelines for reporting parallel group randomised trials. *PLoS Med.* 2010;7(3):e1000251.

20. Luo D, Wan X, Liu J, Tong T. Optimally estimating the sample mean from the sample size, median, mid-range, and/or mid-quartile range. *Stat Methods Med Res.* 2018;27(6):1785-1805.

21. Wan X, Wang W, Liu J, Tong T. Estimating the sample mean and standard deviation from the sample size, median, range and/or interquartile range. *BMC Med Res Methodol.* 2014;14:135.

22. Deeks JJ. Issues in the selection of a summary statistic for meta-analysis of clinical trials with binary outcomes. *Stat Med.* 2002;21(11):1575-1600.

23. Borenstein M, Hedges LV, Higgins JP, Rothstein HR. A basic introduction to fixed-effect and random-effects models for meta-analysis. *Res Synth Methods.* 2010;1(2):97-111.

24. Furukawa TA, Watanabe N, Omori IM, Montori VM, Guyatt GH. Association between unreported outcomes and effect size estimates in Cochrane meta-analyses. *JAMA.* 2007;297(5):468-470.

25. Guyatt GH, Oxman AD, Vist GE, Kunz R, Falck-Ytter Y, Alonso-Coello P, Schunemann HJ, Group GW. GRADE: an emerging consensus on rating quality of evidence and strength of recommendations. *BMJ.* 2008;336(7650):924-926.

26. Acosta-Escribano J, Almanza Lopez S, Plumed Martin L, Garcia Martinez MA, Tajadura Manjarin N. The metoclopramide effect on enteral nutrition tolerance and mechanical ventilation associated pneumonia in neuro critically ill patients. *Nutricion hospitalaria.* 2014;29(6):1345-1351.

27. Chapman MJ, Deane AM, O'Connor SL, Nguyen NQ, Fraser RJL, Richards DB, Hacquoil KE, Vasist Johnson LS, Barton ME, Dukes GE. The effect of camicinal (GSK962040), a motilin agonist, on gastric emptying and glucose absorption in feed-intolerant critically ill patients: a randomized, blinded, placebo-controlled, clinical trial https://www.ncbi.nlm.nih.gov/pmc/articles/PMC4967996/. *Critical care.* 2016;20(1).

28. Doi M, Miyamoto K, Shimokawa T, Ariyasu H, Kaneko M, Yonemitsu T, Nakashima T, Matsumoto H, Shibata M, Shibata N, Soda M, Kitaichi K, Kato S. The effect of standard and high dose of rikkunshito on achievement of enteral nutrition target in critically ill patients: a pilot randomized controlled trial. *Acute medicine and surgery.* 2019.

29. Guo JH, Chen G, Yang SQ, Wei MH, Chen X. Clinical observation of the role of Chenxia Sijunzi decoction in promoting the recovery of gastrointestinal function in critically ill patients. *Zhongguo wei zhong bing ji jiu yi xue [Chinese critical care medicine].* 2012;24(11):674-676.

30. Kooshki A, Khazaei Z, Zarghi A, Rad M, Mohammadi HG, Tabaraie Y. Effects of fenugreek seed powder on enteral nutrition tolerance and clinical outcomes in critically ill patients: a randomized clinical trial. *Biomedical research and therapy.* 2018;5(7):2528-2537.

31. Nassaji M, Ghorbani R, Frozeshfard M, Mesbahian F. Effect of metoclopramide on nosocomial pneumonia in patients with nasogastric feeding in the intensive care unit. *La revue de sante de la Mediterranee orientale / al-Majallah al-sihhiyah li-sharq al-mutawassit [Eastern Mediterranean health journal].* 2010;16(4):371-374.

32. Nursal TZ, Erdogan B, Noyan T, Cekinmez M, Atalay B, Bilgin N. The effect of metoclopramide on gastric emptying in traumatic brain injury. *J Clin Neurosci.* 2007;14(4):344-348.

33. Ritz MA, Chapman MJ, Fraser RJ, Finnis ME, Butler RN, Cmielewski P, Davidson GP, Rea D. Erythromycin dose of 70 mg accelerates gastric emptying as effectively as 200 mg in the critically ill. *Intensive care medicine.* 2005;31(7):949-954.

34. Spapen HD, Duinslaeger L, Diltoer M, Gillet R, Bossuyt A, Huyghens LP. Gastric emptying in critically ill patients is accelerated by adding cisapride to a standard enteral feeding protocol: results of a prospective, randomized, controlled trial. *Critical care medicine.* 1995;23(3):481-485.

35. Yavagal DR, Karnad DR, Oak JL. Metoclopramide for preventing pneumonia in critically ill patients receiving enteral tube feeding: a randomized controlled trial. *Critical care medicine.* 2000;28(5):1408-1411.

36. Heyland DK, Tougas G, Cook DJ, Guyatt GH. Cisapride improves gastric emptying in mechanically ventilated, critically ill patients. A randomized, double-blind trial. *American journal of respiratory and critical care medicine.* 1996;154(6 Pt 1):1678-1683.

37. Mokhtari M, Shariatpanahi Z. Ginger extract dietary supplementation effects on delayed gastric emptying and ventilator-associated pneumonia in adult respiratory distress syndrome patients. *Critical Care.* 2009;1):S56.

38. Rajan V, Maiwall R, Choudhury AK, Jamwal KD, Benjamin J, Kumar G, Joshi YK, Sarin SK. Early addition of prokinetics reverses gut paralysis and improves survival in critically ill cirrhotics-An open label placebo controlled RCT (Feed Intolerance and Treatment-FIT protocol). NCT02528760. *Hepatology.* 2017;66.

39. Sustic A, Zelic M, Protic A, Zupan Z, Simic O, Desa K. Metoclopramide improves gastric but not gallbladder emptying in cardiac surgery patients with early intragastric enteral feeding: randomized controlled trial. *Croatian medical journal.* 2005;46(2):239-244.

40. Tahershamsi F, Rezaei K, Khosravi S, Kheyri Z, Memarzadeh MR, Rafie F. Effect of oral drop gastrolit (Zataria multiflora) on gastric residual volume in mechanically ventilated patients hospitalized in the intensive care units. *Journal of medicinal plants.* 2018;17(68):66-73.

41. Chapman MJ, Fraser RJ, Kluger MT, Buist MD, De Nichilo DJ. Erythromycin improves gastric emptying in critically ill patients intolerant of nasogastric feeding. *Critical care medicine.* 2000;28(7):2334-2337.

42. Deane AM, Lamontagne F, Dukes GE, Neil D, Vasist L, Barton ME, Hacquoil K, Ou X, Richards D, Stelfox HT, Mehta S, Day AG, Chapman MJ, Heyland DK. Nutrition Adequacy Therapeutic Enhancement in the Critically Ill: a Randomized Double-Blind, Placebo-Controlled Trial of the Motilin Receptor Agonist Camicinal (GSK962040): the NUTRIATE Study. *Journal of parenteral and enteral nutrition.* 2018;42(5):949-959.

43. Pinilla JC, Samphire J, Arnold C, Liu L, Thiessen B. Comparison of gastrointestinal tolerance to two enteral feeding protocols in critically ill patients: a prospective, randomized controlled trial. *Journal of Parenteral and Enteral Nutrition* 2001;25(2):81-6.

44. Reignier J, Bensaid S, Perrin-Gachadoat D, Burdin M, Boiteau R, Tenaillon A. Erythromycin and early enteral nutrition in mechanically ventilated patients. *Critical care medicine.* 2002;30(6):1237-1241.

45. Taylor SJ, Allan K, McWilliam H, Manara A, Brown J, Greenwood R, Toher D. A randomised controlled feasibility and proof-of-concept trial in delayed gastric emptying when metoclopramide fails: we should revisit nasointestinal feeding versus dual prokinetic treatment: achieving goal nutrition in critical illness and delayed gastric emptying: trial of nasointestinal feeding versus nasogastric feeding plus prokinetics. *Clinical nutrition ESPEN.* 2016;14:1-8.

46. Boivin MA, Levy H. Gastric feeding with erythromycin is equivalent to transpyloric feeding in the critically ill. *Critical care medicine.* 2001;29(10):1916-1919.

47. Euctr ES. A Phase 2, Multicenter, Randomized, Double-Blind, Comparator-Controlled Study of the Efficacy, Safety, and Pharmacokinetics of Intravenous Ulimorelin (LP101) in Patients with Enteral Feeding Intolerance. *http://www.who.int/trialsearch/Trial2.aspx?TrialID=EUCTR2016.* 2016.

48. Hayakawa M, Ono Y, Wada T, Yanagida Y, Sawamura A, Takeda H, Gando S. Effects of rikkunshito (traditional Japanese medicine) on enteral feeding and the plasma ghrelin level in critically ill patients: a pilot study. *Journal of intensive care.* 2014;2(1).

49. Heyland DK, van Zanten ARH, Grau-Carmona T, Evans D, Beishuizen A, Schouten J, Hoiting O, Bordeje ML, Krell K, Klein DJ, Gonzalez J, Perez A, Brown R, James J, Harris MS, Heyland DK, Evans DC, Jolley S, Raines R, Krell K, Falls I, Grau-Carmona T, Servia-Goixart L, Perez-Quesada S, Herrero-Meseguer JI, Calvo-Herranz E, Lorencio C, Peredes A, Yebenes-Reyes JC, Garcia-Martinez MA, Cervera M, Bordeje ML, Fernadez-Ortega JF, Fernandez-Gonzalez I, van Zanten A, Beishuizen A, Schouten J, Hoiting O, Wilhelmina C, Stelfox T, Posadas J. A multicenter, randomized, double-blind study of ulimorelin and metoclopramide in the treatment of critically ill patients with enteral feeding intolerance: PROMOTE trial. *Intensive care medicine.* 2019;45(5):647-656.

50. Irct201112014578N. The effect of acupuncture and traditional pharmacologic therapy on Delayed Gastric Emptying. *http://www.who.int/trialsearch/Trial2.aspx?TrialID=IRCT201112014578N4*. 2012.

51. Irct201408104365N. Combination of Neostigmine and Metoclopramide to reduce gastric residual volume in mechanically ventilated ICU patients. *http://www.who.int/trialsearch/Trial2.aspx?TrialID=IRCT201408104365N16*. 2015.

52. Irct201412044365N. Comparison of the effect of Neostigmine and Metoclopramide on gastric residual volume in mechanically ventilated ICU patients. *http://www.who.int/trialsearch/Trial2.aspx?TrialID=IRCT201412044365N18*. 2014.

53. MacLaren R, Patrick WD, Hall RI, Rocker GM, Whelan GJ, Lima JJ. Comparison of cisapride and metoclopramide for facilitating gastric emptying and improving tolerance to intragastric enteral nutrition in critically III, mechanically ventilated adults. *Clinical therapeutics.* 2001;23(11):1855-1866.

54. Makkar JK, Gauli B, Jain K, Jain D, Batra YK. Comparison of erythromycin versus metoclopramide for gastric feeding intolerance in patients with traumatic brain injury: a randomized double-blind study. *Saudi journal of anaesthesia.* 2016;10(3):308-313.

55. Malekolkottab M, Khalili H, Mohammadi M, Ramezani M, Nourian A. Metoclopramide as intermittent and continuous infusions in critically ill patients: a pilot randomized clinical trial. *Journal of comparative effectiveness research.* 2017;6(2):127-136.

56. Nct. A Randomized, Double-Blind Study to Evaluate the Safety, Tolerability, and Pharmacodynamics of a Single Dose of Intravenous TD-8954 Compared With Metoclopramide in Critically Ill Patients With Enteral Feeding Intolerance. *https://clinicaltrials.gov/show/NCT01953081*. 2013.

57. Nct. Itopride in Feeding Intolerance of Critically-ill Patients Receiving Enteral Nutrition. *https://clinicaltrials.gov/show/NCT03698292*. 2018.

58. Nguyen N, Chapman M, Fraser R, Sharley V, Kong S, Bryant L, et al. Erythromycin or metoclopramide for feed intolerance in the critically ill. *Critical care.* 2006;10(Suppl 1).

59. Taylor S, Manara A, Brown J. Treating delayed gastric emptying in critical illness: metoclopramide, erythromycin and bedside (Cortrak[TM]) nasointestinal tube placement. *Journal of the intensive care society.* 2011;12(1):75-.

60. Yanagida Y, Hayakawa M, Yamamoto H, Wada T, Sugano M, Sawamura A, Gando S. Effects of rikkunshito (traditional Japanese medicine Kampo) on enteral feeding and plasma ghrelin concentration in critically ill patients: a double-blind, randomized, controlled trial. *Intensive care medicine.* 2013;39:S242-S243.

61. Berne JD, Norwood SH, McAuley CE, Vallina VL, Villareal D, Weston J, McClarty J. Erythromycin reduces delayed gastric emptying in critically ill trauma patients: a randomized, controlled trial. *Journal of trauma.* 2002;53(3):422-425.

62. Meissner W, Dohrn B, Reinhart K. Enteral naloxone reduces gastric tube reflux and frequency of pneumonia in critical care patients during opioid analgesia. *Critical care medicine.* 2003;31(3):776-780.

63. Shariatpanahi ZV, Taleban FA, Mokhtari M, Shahbazi S. Ginger extract reduces delayed gastric emptying and nosocomial pneumonia in adult respiratory distress syndrome patients hospitalized in an intensive care unit. *Journal of critical care.* 2010;25(4):647-650.

64. Tamion F, Hamelin K, Duflo A, Girault C, Richard JC, Bonmarchand G. Gastric emptying in mechanically ventilated critically ill patients: effect of neuromuscular blocking agent. *Intensive care medicine.* 2003;29(10):1717-1722.

65. Chen JH, Hsieh CB, Chao PC, Liu HD, Chen CJ, Liu YC, Yu JC. Effect of water-soluble contrast in colorectal surgery: a prospective randomized trial. *World journal of gastroenterology.* 2005;11(18):2802-2805.

66. Goldhill DR, Toner CC, Tarling MM, Baxter K, Withington PS, Whelpton R. Double-blind, randomized study of the effect of cisapride on gastric emptying in critically ill patients. *Critical care medicine.* 1997;25(3):447-451.

67. Warusevitane A, Karunatilake D, Sim J, Lally F, Roffe C. Safety and effect of metoclopramide to prevent pneumonia in patients with stroke fed via nasogastric tubes trial. *Stroke.* 2015;46(2):454-460.

68. Tctr. Efficacy and safety of oral Erythromycin estolate in combination with Metoclopramide versus Metoclopramide mOnotherapy in mechanically ventilated patients who developed enteral feeding intolerance: a randomized double-blind controlled study. *http://www.who.int/trialsearch/Trial2.aspx?TrialID=TCTR20171004004*. 2017.

69. NCT02528760. To Determine the Role of Prokinetics in Feed Intolerance in Critically Ill Cirrhosis. *https://clinicaltrials.gov/show/NCT02528760*. 2015.

70. NCT02379624. Pectin Start Early Enteral Nutritional Support in Critically Ill Patients. *https://clinicaltrials.gov/show/NCT02379624*. 2014.

71. IRCT201610297494N24. The prophylactic effect of cuminum cyminum extract on gastric residual volume in traumatic patients under ventilator hospitalized in intensive care unit. *http://www.who.int/trialsearch/Trial2.aspx?TrialID=IRCT201610297494N24*. 2017.

72. IRCT201009094722N2. The effect of Ginger extract on gastric residual volume in patients with mechanical ventilation hospitalized in intensive care unit. *http://www.who.int/trialsearch/Trial2.aspx?TrialID=IRCT201009094722N2*. 2011.

73. 2015/08/006111 C. Clinical trial to compare the efficacy of two oral prokinetics metoclopramide and erythromycin in non acceptance of feed in head injury patient. *http://www.who.int/trialsearch/Trial2.aspx?TrialID=CTRI*. 2015;08(006111).

Supporting information

S1 Table. Search ~~S~~strategy

S2 Table. Excluded ~~S~~studies

S3 Table. Separate effects of different prokinetic agents~~s~~ on hospital or ICU length of stay

S4 Table. PRISMA ~~C~~checklist

S1 Fig. Risk of ~~B~~bias

S2 Fig. Reported adverse event~~s~~ outcomes

S3 Fig. All-cause mortality outcomes

S4 Fig. Subgroup ~~A~~analysis by the type of prokinetic agents ~~in outcome of~~for hospital length of stay outcomes

S5 Fig. Subgroup ~~A~~analysis by the type of prokinetic agents ~~in outcome of~~for ICU length of stay outcomes

S6 Fig. Subgroup ~~A~~analysis by the type of prokinetic agents ~~in outcome of~~for reported adverse event outcomes~~s~~

S7 Fig. Subgroup ~~A~~analysis by the type of prokinetic agents ~~in outcome of~~for all-cause mortality outcomes

S8 Fig. Sensitivity analysis ~~in~~of ~~outcome of~~ hospital length of stay outcomes

S9 Fig. Sensitivity analysis ~~in~~of ~~outcome of~~ ICU length of stay outcomes

S10 Fig. Sensitivity analysis ~~in outcome~~ of reported adverse event~~s~~ outcomes

S11 Fig. Sensitivity analysis ~~in outcome~~ of all-cause mortality outcomes

Dear Editors and Reviewers,

Thank you for your letter and the comments concerning our manuscript (Manuscript Number: PONE-D-20-01167R1). We appreciate your positive comments regarding our manuscript. Your suggestions and ideas have been carefully considered. Revised portions are marked with changes in colored fonts in the paper. We hope that the revised manuscript will meet with your approval. The main corrections in the paper and our responses to the reviewers' comments are as follows:

Editors' comments:

The review is greatly improved in clarity and transparency, but requires some minor changes. Please address the comments of the reviewers and in addition:

1. For Line 288-289 where you state that hospital LOS was not significant I suggest you revise to use the same language as in 292-3 for ICU LOS, in that there appears to be a positive effect, unless you wish to clarify in what way the hospital LOS was not significant (clinically?)

   Answer: We sincerely appreciate your thoughtful advice. This mistake was due to our carelessness in writing, and all authors sincerely apologize for this mistake. We have corrected this error. In the revised paper, the text is as follows: "These five studies, which enrolled a total of 250 patients, demonstrated a significant difference in the hospital length of stay between the prokinetic agent-treated group and the control group (MD -3.21, 95% CI -5.35, -1.06; P = 0.003; I2 = 28%) (Fig 2)."

2. For the outcomes of gastrointestinal symptoms and feeding tolerance, you should mention that if outcomes could not be combined by meta-analysis you summarized them narratively. You only discuss in the methods how you will use meta-analysis and then you do not meta-analyse the symptoms and tolerance

outcomes, I assume because they are not appropriate to meta-analyze.

Answer: Yes. The various outcome definitions, especially for gastric tube tolerance, precluded quantitative synthesis of the data. According to your advice, we have amended this part in the revised manuscript as follows: “Thirteen studies evaluated the effect of prokinetics on gastrointestinal symptoms and/or feeding tolerance in adult critically ill patients receiving gastric feeding [26-30, 32-34, 36-40]. The main results obtained are as follows: gastric emptying, GRV, diarrhea, constipation, feeding complications and feeding intolerance. Gastric emptying was measured by the drug model of acetaminophen absorption or the 13C-octanoic acid breath test with calculation of the gastric emptying time, gastric emptying coefficient or area under the plasma concentration-time curve. The various outcome definitions, especially for gastric tube tolerance, precluded quantitative synthesis of the data.”

3. For Line 301 and the outcome of adverse events it is preferable to use the term 'risk' rather than 'incidence' as incidence implies measurement of time at risk.

   Answer: Yes. Following your suggestion, to be more accurate, we have replaced “incidence” with “risk”.

4. For Table 3 please include the units for each outcome, e.g., days and deaths

   Answer: Thank you for this comment. We have revised the manuscript to include the unit for each outcome in Table 3. We hope that this change improves the readability of the data.

5. For all forest plots, including Fig 2, Fig 3, S2Fig and S3Fig, please specify the comparison and the outcome (with units) in the header and replace the bracketed experimental and control on the x axis with a legend indicating the comparisons.

Answer: Following your suggestion, we have added “units” and “legend” to each forest plot. These changes have been made to the text to improve the readability and to clarify the interpretation of the data.

Reviewers' comments:

Reviewer #1:

1. I thank the authors for the significant work done to address al comments and i find the reviewed submission substantially improved.

   Answer: Thank you for your very considerate advice; your positive comment on our manuscript is sincerely appreciated. We will reply to your comments one by one in detail.

2. the length of stay unit for hospital and ICU is still not clear. I assume it is days. However would be nice to have it clarified. See my original comment #11.

   Answer: We apologize for this mistake. The unit for the lengths of hospital stay and ICU stay is “days”. We have added “units” in Table 3 in the revised manuscript. We hope that these changes improve the readability and clarify the interpretation of the data.

3. line 377: "We recommend a more comprehensive search and further original studies on this topic." i recommend the words "more comprehensive search" be deleted as they give the impression the authors did not perform a comprehensive search.

   Answer: Thank you for your thoughtful reminder. We agree with your advice; the words "more comprehensive search" have been deleted in the revised manuscript.

Reviewer #3:

Thank you for the opportunity to review the revised manuscript. In this systematic review, authors evaluated the effect of prokinetics in critically ill adults on gastric feeding tube tolerance according to the updated definition. This systematic review implies that prokinetics improves tolerance of enteral feeding, and additionally provides the attractive hypothesis that prokinetics may shorten the length of ICU and hospital stay. Although authors tried to perform meta-analysis about gastric feeding tube tolerance, study diversity (e.g. various interventions and various outcome definitions) did not allow the authors data synthesis. Authors seems to revise their manuscript well according to the previous editor's and reviewers' comments.

Comments to the authors:

1. As authors state in background, the aim of this study is to evaluate the effect of prokinetics on gastric feeding tube tolerance. So, the main results of this study is the description about this effect (L273-283), not about ICU and hospital length of stay. And one of key points of this study, I believe, is the difficulty to compare results across previous studies because of various outcome definitions, and necessity of the valid measure of gastric tube tolerance in future studies. Authors should add more concise description in this paragraph (L273-283) to show the potential benefit on gastric feeding tube tolerance and clarify the abovementioned point.

   Answer: Thank you for your thoughtful reminder. These comments are valuable and very helpful for revising and improving our paper and provided important guiding significance for our research. According to your advice, we have amended this part in the revised manuscript as follows:

   "Thirteen studies evaluated the effect of prokinetics on gastrointestinal symptoms and/or feeding tolerance in adult critically ill patients receiving gastric feeding [26-30, 32-34, 36-40]. The main results obtained are as follows: gastric emptying,

GRV, diarrhea, constipation, feeding complications and feeding intolerance. Gastric emptying was measured by the drug model of acetaminophen absorption or the 13C-octanoic acid breath test with calculation of the gastric emptying time, gastric emptying coefficient or area under the plasma concentration-time curve. The various outcome definitions, especially for gastric tube tolerance, precluded quantitative synthesis of the data.

As a class of drugs, prokinetic agents appear to have positive effects on gastrointestinal function and improving feeding tolerance. Ten of the thirteen studies reported positive effects on improving gastric emptying and/or resolution of feeding intolerance in critically ill patients with the use of prokinetic agents. However, two studies suggested that metoclopramide had no effect on decreasing gastrointestinal complications in adult neurocritical patients or critical traumatic brain injury patients. One study reported that rikkunshito did not improve the achievement of enteral calorie targets in critically ill adults (Table 2)."

2. L288-290: "Those five studies, enrolling a total of 250 patients, demonstrated that there was no significant difference in hospital length of stay ..."

   Are there any significant difference between groups about the hospital length of stay? 95%CI of -5.35 to -1.06 is significant, isn't it? Please check.

   Answer: I apologize for this mistake. We have corrected this error. In the revised manuscript, the text is as follows: "These five studies, which enrolled a total of 250 patients, demonstrated a significant difference in the hospital length of stay between the prokinetic agent-treated group and the control group (MD -3.21, 95% CI -5.35, -1.06; P = 0.003; I2 = 28%) (Fig 2)".

Thank you again for your attention and thoughtful advice. We hope that the revised manuscript will meet with your approval.
