# Supplementary material for: The efficacy and safety of prokinetics in critically ill adults receiving gastric feeding tubes: A systematic review and meta-analysis
Source: PLoS One. 2021 Jan 11;16(1):e0245317. doi: 10.1371/journal.pone.0245317 (PMC7799841; doi:10.1371/journal.pone.0245317)
Supplement: Attachment — Submitted filename: renamed_7d017.docx [file pone.0245317.s016.docx]

**Dear Editors and Reviewers,**

Thank you for your letter and the comments concerning our manuscript entitled “The efficacy and safety of prokinetics in adult critically ill patients receiving gastric enteral nutrition: a systematic review and meta-analysis” (Manuscript Number: PONE-D-20-01167). Those comments are all valuable and very helpful for revising and improving our paper, as well as the important guiding to our researches. We have studied comments carefully and have made correction which we hope meet with approval. Revised portion are marked with the changes highlighted in yellow in the paper. The main corrections in the paper and the responds to the reviewer’s comments are as flowing:

**Editors' comments:**

We appreciate your generous help and your patience. We take all the comments into consideration, and revise our manuscript accordingly. We hope the revised manuscript can meet with your approval. The answers to your comments are as flowing:

1. Although the updated definition of feeding intolerance provides a rationale for a new examination of the question, it appears from the reviewer comments that studies without a clear definition were included (eg, Tahershamsi 2018). Please clarify why these were selected for inclusion.

**Answer:** In our study, except for feeding tolerance, the main outcomes included gastrointestinal symptoms and clinical outcomes (hospital length of stay, ICU length of stay, reported adverse events, all-cause mortality). The included studies were not based on whether the study with a clear definition of feeding intolerance. But we excluded these studies that discontinued or interrupted the gastric feeding prematurely when the GRV was less than 500 mL or the patients did not have any signs of intolerance.

2. It also appears from the reviewer comments that several relevant studies may have been missed. This issue could be clarified by specifying the references and exclusion criteria for the 27 studies excluded at full text. This could be done in a separate online table if necessary.

**Answer:** all the literatures had been checked again. The inclusion and exclusion studies all met the predefined criteria. And according to your advice, we added a table to explain the exclusion reason for the 27 studies. (**Supp. Table 2**).

3. The reviewer who noted the inclusion of hospital length of stay in the Results but not in the Methods is correct. Please clarify this.

**Answer:** we are very appreciated of your kindly advise. Because of our carelessness, “hospital length of stay” did not present in the Inclusion Criteria of Methods section. But the outcome of “hospital length of stay” have already been included in our plan. In the Methods section of the Data Extraction and Statistical Analysis, the outcome of “hospital length of stay” had been included. So, we amended the Methods section in manuscript. we added the outcome of “hospital length of stay” in the Inclusion Criteria of Methods section.

4. Pay attention to reviewer comments highlighting the problem with combining different interventions in the meta-analysis. For example, in the herbal group of studies, why does it make sense to combine rikkunshito and fenugreek, and why does it make sense to produce a summary estimate across all the different types of interventions? Please clarify or revise.

**Answer:** According to your advice, we have amended the relevant part in manuscript. In the revised manuscript, we removed the results of the combining herbal group in the meta-analysis. However, as a class of drugs, the results of the combining different types of prokinetics in the meta-analysis were reserved. Although no significant heterogeneity was found, we performed subgroup analyses to determine if there are important subgroup differences.

5. The reviewer comments about GRADE evaluations should be responded to. For example, imprecision is present in several analyses based on rule-of-thumb criteria for sample size (eg, at least 400 participants or 300 events in an analysis), as well as potentially based on width of the confidence interval including both benefit and lack of effect. The criteria for decisions about GRADE elements should be specified in the Methods section, and the rationale for decisions should be made clear through footnotes to Table 2.

**Answer:** we felt sorry for these mistakes. We seriously conducted the GRADE evaluation again. In the revised manuscript, the details of the risk of bias and quality assessment were outlined in Table 3, the rationale for decisions had been made clear through footnotes to Table 3.

6. Regarding publication bias and GRADE, it is appropriate that funnel plots were not created however the rationale for strongly suspecting publication bias in all estimates should be clarified.

**Answer:** In view of the fact that publication bias arises when investigators fail to report studies they have undertaken (typically those that show no effect). A prototypical situation that should elicit suspicion of publication bias occurs when published evidence is limited to a small number of trials, all of which are showing benefits of the studied intervention.

In the revised manuscript, the meta-analysis showed that the use of prokinetic agents in critically ill patients receiving gastric feeding may reduce ICU or hospital length of stay, there was no significant difference in the incidence of reported adverse events and all-cause mortality between prokinetic agent group and control group. Although we could not assess this category reliably due to small number of eligible studies, we did not downgrade the quality of evidence for publication bias. Because not all of included studies were showing benefits of the studied intervention in clinical outcome of ICU length of stay and hospital length of stay, respectively.

7. Under funding disclosure, it is unclear whether the review was funded, and if so by whom. This needs to be specified.

**Answer:** according to your advice, we have amended the relevant part in manuscript.

8. The Plos ONE guidelines for systematic reviews state that: ‘Authors must also state in their “Methods” section whether a protocol exists for their systematic review, and if so, provide a copy of the protocol as supporting information and provide the registry number in the “abstract”. Please include this information.

**Answer:** we have registered our protocol in <https://www.crd.york.ac.uk/prospero/>,

but the review is still ongoing (ID 157446).

9. The review Methods should include a description of the procedures for screening of titles/abstracts and full texts.

**Answer:** according to your advice, we have amended the relevant part in manuscript. The details of the eligible trials are presented in Figure 1. Reasons for excluding studies included that the study had a different trial design [41-45]; the study had a different intervention or a different control [46-64]; the study had a different population [65-67]; or the study had been registered with the Clinical Trials Registry Platform (clinicaltrials.gov or WHO ICTRP) and had been labeled “completed”, but outcomes did not report [68-73] (Supp. Table 2).

10. The I-squared is not an evaluation of the statistical heterogeneity between summary data. It is an estimate of the percentage of variability in a meta-analysis that is attributable to study heterogeneity.

The choice of a random vs fixed effects model should not be based on heterogeneity according to the Cochrane Handbook and other sources. For more information on choice of models see page 105 of https://www.meta-analysis.com/downloads/Intro_Models. pdf.

**Answer:** We appreciate your generous help for the references. In the revised manuscript, a random-effects model was used. However, if the number of studies is very small, the statistical power will have poor precision due to the variance between studies. Although the random-effects model is still the appropriate model, the information to apply it correctly is not available. In this case, we added the separate effects to our manuscript. If heterogeneity was identified (I^2^ >40%) and there were sufficient trials included in the review, we planned to investigate heterogeneity in the specified subgroups based on types of prokinetics (erythromycin, metoclopramide or other prokinetics), combination of prokinetics (yes or no), and feeding intolerance history (participants with or without pre-existing feeding intolerance before the start of the trial). Analysis was performed to assess whether the difference between the subgroups was statistically significant.

Thanks again for your help and your advice. With the help of American Journal Experts (AJE), we corrected errors in grammar and spelling in our manuscript. We also hope that the revised manuscript can meet with your approval.

**Reviewers' comments:**

Reviewer #1:

Thanks a lot for your very considerate advice. In the revised manuscript, there were some substantially modified in our results and conclusions sections. we removed the results of the combining herbal group in the meta-analysis. A random-effects model was used. However, if the number of studies is very small, the statistical power will have poor precision due to the variance between studies. Although the random-effects model is still the appropriate model, the information to apply it correctly is not available. In this case, we added the separate effects to our manuscript. In addition, with the help of American Journal Experts (AJE), we corrected errors in grammar and spelling in our manuscript.

we will reply your comments one by one in details.

1. Paragraph 115-118 needs revision. Fragment?

**Answer:** yes, we have amended the relevant part in manuscript.

In the revised essay, it is shown as follows: if the gastric feeding patients with feeding intolerance had a GRV ≥500 mL and/or symptoms of nausea, vomiting, abdominal distention, regurgitation, deterioration in hemodynamics or other symptoms resulting in feeding interruption, and if they failed to respond to intervention, then, regardless of whether they were in the control group or the prokinetics group, they were switched to postpyloric feeding or had gastric feeding withheld for 4-6 h.

2. Line 142: end of sentence “changes of electrocardiogram QTc interval, and so on”. Perhaps removing “and so on” will sound better or can refer to outcomes listed in the paragraph of “inclusion criteria”.

**Answer:** according to your advice, we have listed the outcomes of reported adverse events in the paragraph of “inclusion criteria”. In the revised essay, it is showed as follows: A reported adverse event was defined as any untoward medical occurrence or unfavorable and unintended sign, including an abnormal laboratory finding, symptom, or disease (new or exacerbated), temporally associated with the use of the study medication. The reported adverse events included abnormal laboratory test results (hematology, clinical chemistry, or urinalysis) or other safety assessments (e.g., ECGs, radiological scans, or measurements of vital signs), including those that worsened from baseline, and were deemed clinically significant in the medical and scientific judgment of the investigator; exacerbation of a chronic or intermittent preexisting condition, including an increase in the frequency and/or intensity of the condition; new conditions detected or diagnosed after the administration of study medication even if they may have been present prior to the start of the study; and/or signs, symptoms, or clinical sequelae of a suspected interaction, such as, diarrhea, nosocomial pneumonia, severe sepsis, brain herniation, cardiac arrest, or changes in the electrocardiographic QTc interval.

3. Lines 252-254: for consistency, report results fully with point estimates and 95% CIs.

**Answer:** Yes, in the revised manuscript, all the results were fully with point estimates and 95% CIs.

4. Lines 256-259: what do the authors mean by “the total situation”. “And there was also no significant difference in the risk of the reported adverse events between prokinetics treatment and the control group on the total situation (RR, 1.18, 95%CI, 0.79 to 1.75, P=0.42; I^2^=69%).

**Answer:** in the revised manuscript, this part had a big change in data analysis. In the outcome of reported adverse events, it is showed as follows: “Seven studies reported events that met the definition of adverse events in 757 critically ill patients [26-28, 30-32, 35]. The meta-analysis showed that there was no significant difference in the incidence of reported adverse events between the prokinetic agent group and the control group (RR 1.13, 95% CI 0.92, 1.38; P = 0.25; I2 = 0%) (Supp. Figure 2)”.

5. Lines 264: can the authors explain why they deviated from their data analysis plan of conducting publication bias assessment when 10 or more studies were available (lines 181-183) but lowered the quality of evidence because publication bias was strongly suspected?

**Answer:** In the revised manuscript, the meta-analysis showed that the use of prokinetic agents in critically ill patients receiving gastric feeding may reduce ICU or hospital length of stay, there was no significant difference in the incidence of reported adverse events and all-cause mortality between prokinetic agent group and control group. Although we could not assess this category reliably due to small number of eligible studies, **we did not downgrade the quality of evidence for publication bias.** The reasons are chiefly as follows: in view of the fact that publication bias arises when investigators fail to report studies they have undertaken (typically those that show no effect). A prototypical situation that should elicit suspicion of publication bias occurs when published evidence is limited to a small number of trials, all of which are showing benefits of the studied intervention. However, in our study, not all of included studies were showing benefits of the studied intervention in clinical outcome of ICU length of stay and hospital length of stay, respectively.

6. Line 271-272: “Subgroup analysis by type of prokinetics.” Fragment.

**Answer:** yes, we have amended the relevant part in manuscript.

it is showed as follows: “Although no significant heterogeneity was found, we performed subgroup analyses to determine if there are important subgroup differences. In the subgroup analysis stratified by type of prokinetic agents, there were no significant subgroup difference in the clinical outcomes of hospital length of stay, ICU length of stay, reported adverse events and all-cause mortality (Supp. Figure 4-7)”.

7. Line 275-277: When Metoclopramide did not show significant difference in the hospital length of stay compared with the control group (MD, 1.70, 95%CI, -4.75 to 8.15, P=0.61; I2=0%) (S1 Fig). Check grammar please.

**Answer:** In the revised manuscript, we did not report the outcome of subgroup analysis by specific drug. Because the number of studies is very small, the statistical power will have poor precision due to the variance between studies. We added a table of the separate effects of different prokinetics on the ICU length of stay and hospital length of stay in Supp. Table 3.

8. Lines 283-284: repetition of 254-256.

**Answer:** we have amended the relevant part in manuscript.

9. Lines 285-286: review grammar.

Line 286-287: pooled effects for what outcome(s)?

**Answer:** we have amended the relevant part in manuscript.

10. Line 288: I think the authors mean to have the first part as a subheading “Subgroup analysis by preventive giving prokinetics or therapeutic usage of prokinetics.” Would recommend: preventive use instead of preventive giving. Also would recommend: “pooled effects for primary outcomes” instead of “pooled effects” at the end of sentences.

**Answer:** yes, we accept your opinions, and we have amended the relevant part in manuscript. In the revised manuscript, this part is amended as follows: Furthermore, there was no study comparing the combination of prokinetics to placebo or no treatment. Only one study about the preventive usage of prokinetics for risk patients demonstrated the outcome of reported adverse events [27]. The others were about the preventive usage of prokinetics for all patients. The subgroup analysis result of the preventive usage of prokinetics for all patients did not show important changes in the pooled effects of the reported adverse events.

11. Lines 298-301: the authors deviate from statistical plan for publication bias again by downgrading for string suspicion of publication bias. Revision needed. Also in GRADE evidence profile confidence intervals for all-cause mortality, ICU and hospital length of stay and adverse events all include significant benefits and harms and yet no downgrading for imprecision was performed. Can the authors elaborate on this ? ¬¬¬For length of stay what was the unit of measurement? Days or hours? This would be important for the outcome of hospital length of stay with natural medicines. If the difference was 9 days fewer and between 18.5 and 0.8 days lower, one might consider downgrading for imprecision specially that the number of patients in that analysis is 122 only. I would strongly recommend the authors consult someone with more experience using the GRADE method.

**Answer:** we felt sorry for these mistakes. We seriously conducted the GRADE evaluation again. The result of GRADE is amended as follows: The certainty of evidence was moderate for the clinical outcome of all-cause mortality. However, the certainty of evidence was low for the clinical outcomes of ICU length of stay, hospital length of stay and reported adverse events. The details of the risk of bias and quality assessment are outlined in Table 3. The rationale for decisions had been made clear through footnotes to Table 3.

12. Lines 315-318: And there was a systematic review about the definition, prevalence, and outcome of feeding intolerance in intensive care, and the result showed that there were more than 60% (40/63) of studies defining the large GRVs with the threshold less than or equal to 250 ml and about 41.7% (30/72) of studies with the sole GRVs levels to definite of feeding intolerance. Not clear and needs to be rewritten.

**Answer:** yes, we have amended the relevant part in manuscript. The revised sentences are as follows: There are three methods for the treatment of gastric feeding intolerance. First, there is the most widely used method, the administration of prokinetics. Among recipients of gastric feeding, 13% had been prescribed prokinetics preemptively before they developed intolerance. Approximately one-third of patients who developed feeding intolerance were treated with a prokinetic agent during their stay in the ICU. Second, after the development of intolerance, 17% of patients received supplemental parenteral nutrition. Third, only 7.5% of patients with gastric feeding intolerance subsequently received enteral nutrition via a postpyloric feeding tube [8]. (In the revised essay, the revised sentences are in the part of “**Introduction**”).

13. Lines 325-327: So, we conducted this updated systematic review and meta-analysis, we defined the gastric enteral nutrition feeding intolerance as either GRV ≥500 ml or GRV <500 ml concomitant with symptoms of nausea, vomiting, abdominal distention, regurgitation or other symptoms resulting in feeding interruption. This is not true. Only 3 of the included studies explicitly reported the cutoff for the GRV to be ≥ 500 ml (24, 31, 34) and 1 study reported > 200 ml at any time or > 500 ml/24 hours). I find this misleading.

Line 342: Lewis, K. et al [12] defined feeding intolerance as either GRV ≥150 ml, vomiting, or abdominal distention resulting in feeding interruption. This definition may be considered obsolete [13]. This is very difficult to justify given the point above when the exact amount of GRV was not reported in more than half of the studies included.

**Answer:** yes, we fully understand your purpose. We need to clarify the aims and objectives of our study. In this study, except for feeding tolerance, we also examined the effect of prokinetic agents on gastrointestinal symptoms and clinical outcomes. We defined gastric feeding intolerance as either GRV ≥500 ml or concomitant with symptoms of nausea, vomiting, abdominal distention, regurgitation or other symptoms resulting in feeding interruption in critically ill adult patients receiving gastric feeding tubes. We excluded studies that discontinued or interrupted gastric feeding prematurely following the disappearance of gastric feeding intolerance. Under this latest definition, our meta-analysis found some new studies [26-30, 33, 34], besides, we identified 5 studies regarding the administration of prokinetics of herbal medicines/natural medicines in patients receiving gastric feeding tube in critically ill adults [28-30, 37, 40].

14. Lines 342-347 are exact repetition of lines 324-329. Line 351: “trails” change to “trials”

**Answer:** Thank you for your warm reminder. We improve the quality of the English throughout our manuscript with the help of American Journal Experts (AJE).

15. Lines 352-358: I disagree with the conclusion. The heterogeneity was 95%, the confidence intervals we extremely wide (especially if this was in days) and the total number of patients is 122. This makes it extremely difficult to make any sound conclusions, contrary to the way reported by the authors.

**Answer:** In the revised manuscript, we were careful to generalize the conclusion of this article. we removed the results of the combining herbal group in the meta-analysis. “we found that prokinetic agents might reduce ICU or hospital length of stay in critically ill patients receiving gastric feeding. However, the number of studies and the sample size were very small, and the certainty of evidence was low. Furthermore, there was no significant difference between prokinetic agent groups and placebo/no treatment in the risk of reported adverse events and all-cause mortality. Therefore, we cannot draw a convincing conclusion that the use of prokinetics could improve clinical outcomes in critically ill adults. We recommend a more comprehensive search and further original studies on this topic”.

16. Lines 380: I am concerned that the while the title of the, manuscript is prokinetics, the bulk of discussion section and the conclusion is about herbal medicines as prokinetic agents. As mentioned earlier, the authors claim their cutoff for GRV was 500 ml but they included many studies without a clear cutoff and by doing that they missed many studies in Lewis et al. that included generic medicines. In addition, the authors did not include hospital length of stay as an outcome in their methods section (lines 92-101) and yet they perform this analysis and include the forest plot for it in the main manuscript.

The methods section very well be written. However, the other sections need significant revision of language and grammar.

**Answer:** your comment on the methods section is a very important recognition for our manuscript. The revised portion are marked with the changes highlighted in yellow in the paper. We hope the revised manuscript meet you’re your approval.

**Reviewer #2:**

Thank you for your warm reminder. Your suggestions and ideas have been highly regarded. We will reply your comments with further instructions.

The authors made a new meta-analysis on the efficacy and safety of prokinetic agents administered during enteral feeding in intensive care patients. Previous meta-analysis was already performed on this topic. Originality of this work may come from a revised definition for digestive feeding intolerance, which indeed corresponds to more recent experts’ recommendations (Gastric residual volume (GRV) above 500mls). It has to be noted however, that even the threshold of 500mls GRV for definition of gastric enteral feeding intolerance is challenged, and some authors now recommend against measuring any GRV during enteral nutrition in ICU patients.

I have two major concerns with that study:

1. Subgroup analysis: what is the rationale of pooling studies in which different substances are administered: indeed, natural/herbal substances are not identical in these pooled studies.

**Answer:** first, some studies have suggested that measurement of GRV provides no benefit and should no longer be recommended. However, GRV is also an indicator of feeding intolerance in many ICUs, especially in patients with a high risk of aspiration and aspiration pneumonia. Therefore, the Chinese guidelines call for caution in abandoning monitoring of GRV in some high-risk patients.

Second, after careful consideration, we agree with your advice, the natural/herbal substances are not identical in these pooled studies. In the revised manuscript, we have removed the pooled effect of the natural/herbal substances. Besides, although no significant heterogeneity was found, we performed subgroup analyses to determine if there are important subgroup differences. In the subgroup analysis stratified by type of prokinetic agents, there were no significant subgroup difference in the clinical outcomes of hospital length of stay, ICU length of stay, reported adverse events and all-cause mortality (**Supp. Figure 4-7**).

We found that prokinetic agents might reduce ICU or hospital length of stay in critically ill patients receiving gastric feeding. However, the number of studies and the sample size were very small, and the certainty of evidence was low. Furthermore, there was no significant difference between prokinetic agent groups and placebo/no treatment in the risk of reported adverse events and all-cause mortality. Therefore, we cannot draw a convincing conclusion that the use of prokinetics could improve clinical outcomes in critically ill adults. We recommend a more comprehensive search and further original studies on this topic.

2. To my opinion, the conclusion (administration of herbal medicines/natural medicines may reduce hospital length of stay) is not supported by the (meta)analysis. The purpose of a meta-analysis is to pool pertinent studies in order to answer a well predefined question. The conclusion that is drawn by the authors is not really related to the initial question.

**Answer:** we amended the relevant part in manuscript carefully. Revised portion are marked with the changes highlighted in yellow in the paper. there were some substantially modified in our results and conclusions sections.

In the revised manuscript, the conclusion was showed as follows: As a class of drugs, prokinetic agents may improve gastric feeding tolerance in critically ill adults. However, there is low certainty in the evidence that prokinetic agents are effective in reducing ICU or hospital length of stay. There was also no significant reduction in the risk of reported adverse events and all-cause mortality. Additional RCTs are needed to determine the effect of prokinetics on clinical outcomes in critically ill patients in the future.

Thank you very much for your attention and kindly advise. We hope the revised manuscript meet with your approval.
