# Supplementary material for: The efficacy and safety of prokinetics in critically ill adults receiving gastric feeding tubes: A systematic review and meta-analysis
Source: PLoS One. 2021 Jan 11;16(1):e0245317. doi: 10.1371/journal.pone.0245317 (PMC7799841; doi:10.1371/journal.pone.0245317)
Supplement: Attachment — Submitted filename: Response to Reviewers_Final20200625.docx [file pone.0245317.s017.docx]

**Dear Editors and Reviewers,**

Thank you for your letter and the comments concerning our manuscript (Manuscript Number: PONE-D-20-01167R1). We appreciate your positive comments regarding our manuscript. Your suggestions and ideas have been carefully considered. Revised portions are marked with changes in colored fonts in the paper. We hope that the revised manuscript will meet with your approval. The main corrections in the paper and our responses to the reviewers’ comments are as follows:

**Editors' comments:**

The review is greatly improved in clarity and transparency, but requires some minor changes. Please address the comments of the reviewers and in addition:

1. For Line 288-289 where you state that hospital LOS was not significant I suggest you revise to use the same language as in 292-3 for ICU LOS, in that there appears to be a positive effect, unless you wish to clarify in what way the hospital LOS was not significant (clinically?)

**Answer:** We sincerely appreciate your thoughtful advice. This mistake was due to our carelessness in writing, and all authors sincerely apologize for this mistake. We have corrected this error. In the revised paper, the text is as follows: “These five studies, which enrolled a total of 250 patients, demonstrated a significant difference in the hospital length of stay between the prokinetic agent-treated group and the control group (MD -3.21, 95% CI -5.35, -1.06; P = 0.003; I2 = 28%) (Fig 2).”

2. For the outcomes of gastrointestinal symptoms and feeding tolerance, you should mention that if outcomes could not be combined by meta-analysis you summarized them narratively. You only discuss in the methods how you will use meta-analysis and then you do not meta-analyse the symptoms and tolerance outcomes, I assume because they are not appropriate to meta-analyze.

**Answer:** Yes. The various outcome definitions, especially for gastric tube tolerance, precluded quantitative synthesis of the data. According to your advice, we have amended this part in the revised manuscript as follows: “Thirteen studies evaluated the effect of prokinetics on gastrointestinal symptoms and/or feeding tolerance in adult critically ill patients receiving gastric feeding [26-30, 32-34, 36-40]. The main results obtained are as follows: gastric emptying, GRV, diarrhea, constipation, feeding complications and feeding intolerance. Gastric emptying was measured by the drug model of acetaminophen absorption or the 13C-octanoic acid breath test with calculation of the gastric emptying time, gastric emptying coefficient or area under the plasma concentration-time curve. The various outcome definitions, especially for gastric tube tolerance, precluded quantitative synthesis of the data.”

3. For Line 301 and the outcome of adverse events it is preferable to use the term 'risk' rather than 'incidence' as incidence implies measurement of time at risk.

**Answer:** Yes. Following your suggestion, to be more accurate, we have replaced “incidence” with “risk”.

4. For Table 3 please include the units for each outcome, e.g., days and deaths

**Answer:** Thank you for this comment. We have revised the manuscript to include the unit for each outcome in Table 3. We hope that this change improves the readability of the data.

5. For all forest plots, including Fig 2, Fig 3, S2Fig and S3Fig, please specify the comparison and the outcome (with units) in the header and replace the bracketed experimental and control on the x axis with a legend indicating the comparisons.

**Answer:** Following your suggestion, we have added “units” and “legend” to each forest plot. These changes have been made to the text to improve the readability and to clarify the interpretation of the data.

**Reviewers' comments:**

**Reviewer #1:**

1. I thank the authors for the significant work done to address al comments and i find the reviewed submission substantially improved.

**Answer:** Thank you for your very considerate advice; your positive comment on our manuscript is sincerely appreciated. We will reply to your comments one by one in detail.

2. the length of stay unit for hospital and ICU is still not clear. I assume it is days. However would be nice to have it clarified. See my original comment #11.

**Answer:** We apologize for this mistake. The unit for the lengths of hospital stay and ICU stay is “days”. We have added “units” in Table 3 in the revised manuscript. We hope that these changes improve the readability and clarify the interpretation of the data.

3. line 377: "We recommend a more comprehensive search and further original studies on this topic." i recommend the words "more comprehensive search" be deleted as they give the impression the authors did not perform a comprehensive search.

**Answer:** Thank you for your thoughtful reminder. We agree with your advice; the words "more comprehensive search" have been deleted in the revised manuscript.

**Reviewer #3:**

Thank you for the opportunity to review the revised manuscript. In this systematic review, authors evaluated the effect of prokinetics in critically ill adults on gastric feeding tube tolerance according to the updated definition. This systematic review implies that prokinetics improves tolerance of enteral feeding, and additionally provides the attractive hypothesis that prokinetics may shorten the length of ICU and hospital stay. Although authors tried to perform meta-analysis about gastric feeding tube tolerance, study diversity (e.g. various interventions and various outcome definitions) did not allow the authors data synthesis. Authors seems to revise their manuscript well according to the previous editor's and reviewers' comments.

Comments to the authors:

1. As authors state in background, the aim of this study is to evaluate the effect of prokinetics on gastric feeding tube tolerance. So, the main results of this study is the description about this effect (L273-283), not about ICU and hospital length of stay. And one of key points of this study, I believe, is the difficulty to compare results across previous studies because of various outcome definitions, and necessity of the valid measure of gastric tube tolerance in future studies. Authors should add more concise description in this paragraph (L273-283) to show the potential benefit on gastric feeding tube tolerance and clarify the abovementioned point.

**Answer:** Thank you for your thoughtful reminder. These comments are valuable and very helpful for revising and improving our paper and provided important guiding significance for our research. According to your advice, we have amended this part in the revised manuscript as follows:

“Thirteen studies evaluated the effect of prokinetics on gastrointestinal symptoms and/or feeding tolerance in adult critically ill patients receiving gastric feeding [26-30, 32-34, 36-40]. The main results obtained are as follows: gastric emptying, GRV, diarrhea, constipation, feeding complications and feeding intolerance. Gastric emptying was measured by the drug model of acetaminophen absorption or the 13C-octanoic acid breath test with calculation of the gastric emptying time, gastric emptying coefficient or area under the plasma concentration-time curve. The various outcome definitions, especially for gastric tube tolerance, precluded quantitative synthesis of the data.

As a class of drugs, prokinetic agents appear to have positive effects on gastrointestinal function and improving feeding tolerance. Ten of the thirteen studies reported positive effects on improving gastric emptying and/or resolution of feeding intolerance in critically ill patients with the use of prokinetic agents. However, two studies suggested that metoclopramide had no effect on decreasing gastrointestinal complications in adult neurocritical patients or critical traumatic brain injury patients. One study reported that rikkunshito did not improve the achievement of enteral calorie targets in critically ill adults (Table 2).”

2. L288-290: "Those five studies, enrolling a total of 250 patients, demonstrated that there was no significant difference in hospital length of stay ..."

Are there any significant difference between groups about the hospital length of stay? 95%CI of -5.35 to -1.06 is significant, isn't it? Please check.

**Answer:** I apologize for this mistake. We have corrected this error. In the revised manuscript, the text is as follows: “These five studies, which enrolled a total of 250 patients, demonstrated a significant difference in the hospital length of stay between the prokinetic agent-treated group and the control group (MD -3.21, 95% CI -5.35, -1.06; P = 0.003; I2 = 28%) (Fig 2)”.

Thank you again for your attention and thoughtful advice. We hope that the revised manuscript will meet with your approval.
