# Supplementary material for: The efficacy and safety of prokinetics in critically ill adults receiving gastric feeding tubes: A systematic review and meta-analysis
Source: PLoS One. 2021 Jan 11;16(1):e0245317. doi: 10.1371/journal.pone.0245317 (PMC7799841; doi:10.1371/journal.pone.0245317)
Supplement: Attachment — Submitted filename: REBUTTAL_LETTER_20201201.docx [file pone.0245317.s019.docx]

**Dear Editors and Reviewers,**

Thank you for your letter and the comments concerning our manuscript (Manuscript Number: PONE-D-20-01167R2). We appreciate your positive comments on our manuscript. Your suggestions and ideas have been carefully considered. The revised portions are in red font in the revised version of the paper. We hope the revised manuscript will meet with your approval. The main corrections in the paper and the responses to the reviewers’ comments are as follows:

**Editors' comments:**

Please address the comments from the reviewer on the attachment, and consider shortening the introduction as requested.

**Answer:** We are very appreciative of your advice. We have replied to the reviewers' comments in a point-by-point manner.

**Reviewers' comments:**

**Reviewer #3:**

1. If the authors have adequately addressed your comments raised in a previous round of review and you feel that this manuscript is now acceptable for publication, you may indicate that here to bypass the “Comments to the Author” section, enter your conflict of interest statement in the “Confidential to Editor” section, and submit your "Accept" recommendation.

Reviewer #3: All comments have been addressed

**Answer:** We appreciate your positive comments on our manuscript. Thank you for your hard work reviewing our manuscript.

**Reviewer #4:**

1. If the authors have adequately addressed your comments raised in a previous round of review and you feel that this manuscript is now acceptable for publication, you may indicate that here to bypass the “Comments to the Author” section, enter your conflict of interest statement in the “Confidential to Editor” section, and submit your "Accept" recommendation.

Reviewer #4: All comments have been addressed

2. Is the manuscript technically sound, and do the data support the conclusions?

The manuscript must describe a technically sound piece of scientific research with data that supports the conclusions. Experiments must have been conducted rigorously, with appropriate controls, replication, and sample sizes. The conclusions must be drawn appropriately based on the data presented.

Reviewer #4: Yes

3. Has the statistical analysis been performed appropriately and rigorously?

Reviewer #4: Yes

4. Have the authors made all data underlying the findings in their manuscript fully available?

The [PLOS Data policy](http://www.plosone.org/static/policies.action#sharing) requires authors to make all data underlying the findings described in their manuscript fully available without restriction, with rare exception (please refer to the Data Availability Statement in the manuscript PDF file). The data should be provided as part of the manuscript or its supporting information, or deposited to a public repository. For example, in addition to summary statistics, the data points behind means, medians and variance measures should be available. If there are restrictions on publicly sharing data—e.g. participant privacy or use of data from a third party—those must be specified.

Reviewer #4: Yes

5. Is the manuscript presented in an intelligible fashion and written in standard English?

PLOS ONE does not copyedit accepted manuscripts, so the language in submitted articles must be clear, correct, and unambiguous. Any typographical or grammatical errors should be corrected at revision, so please note any specific errors here.

Reviewer #4: Yes

6. Review Comments to the Author

Please use the space provided to explain your answers to the questions above. You may also include additional comments for the author, including concerns about dual publication, research ethics, or publication ethics. (Please upload your review as an attachment if it exceeds 20,000 characters)

Reviewer #4: Minor clarifications line (see attached):
36
73
355

Also the introduction should be made more concise in this long paper.

**Answer:** We appreciate your positive comments on our manuscript. Your comments were valuable when we revised and improved our paper. According to your advice, we have amended those parts in the revised manuscript. We will reply to your comments one by one in detail.

(1) Line 36: Equivocal: Continuous gastric feeding is not physiological, though possibly safer than bolus feeding (more physiological) regarding potential aspiration. Continuous intestinal feeding may be therefore actually more physiological.

**Answer:** We agree with you. We apologize that we did not express ourselves very clearly. We would like to present our standpoint that feeding administered in the stomach is usually considered more physiological than postpyloric feeding.

(2) Line 73: These sentences: “Some studies have suggested that measurement of GRV provides no benefit and should no longer be recommended. However, GRV is also an indicator of feeding intolerance in many ICUs.” were deleted.

**Answer:** Following your suggestion, we have deleted these sentences.

(3) Line 355: The negative studies (3 of 13, 23.08%) were hampered by special populations of neuro-critical patients and critical traumatic brain injury patients taking metoclopramide and by the use of the specific drug rikkunshito.

**Answer:** I am very sorry that we did not state it clearly. We have amended this part in the revised manuscript. It is shown as follows: “The studies that did not show beneficial effects (3 of 13, 23.08%) investigated special populations of neurocritical patients and critical traumatic brain injury patients taking metoclopramide and rikkunshito.”

(4) Also the introduction should be made more concise in this long paper.

**Answer:** Thank you for your reminder. These comments were very helpful for revising and improving our paper. According to your advice, we have amended this part in the revised manuscript.

Thank you again for your attention and advice. We hope the revised manuscript will meet with your approval.
